# NNKTT120, an anti-iNKT cell monoclonal antibody, produces rapid and sustained iNKT cell depletion in adults with sickle cell disease

**DOI:** 10.1371/journal.pone.0171067

**Published:** 2017-02-02

**Authors:** Joshua J. Field, Elaine Majerus, Kenneth I. Ataga, Elliot P. Vichinsky, Robert Schaub, Robert Mashal, David G. Nathan

**Affiliations:** 1 Medical Sciences Institute, Blood Center of Wisconsin, Milwaukee, Wisconsin, United States of America; 2 Department of Medicine, Medical College of Wisconsin, Milwaukee, Wisconsin, United States of America; 3 Department of Medicine, Washington University in St. Louis, St. Louis, Missouri, United States of America; 4 University of North Carolina, Chapel Hill, North Carolina, United States of America; 5 Oakland Children’s Hospital, Oakland, California, United States of America; 6 NKT Therapeutics, Waltham, Massachusetts, United States of America; 7 Dana-Farber Cancer Institute, Boston, Massachusetts, United States of America; 8 Boston Children’s Hospital, Boston, Massachusetts, United States of America; 9 Harvard Medical School, Boston, Massachusetts, United States of America; Medizinische Universitat Graz, AUSTRIA

## Abstract

Invariant NKT (iNKT) cells can be activated to stimulate a broad inflammatory response. In murine models of sickle cell disease (SCD), interruption of iNKT cell activity prevents tissue injury from vaso-occlusion. NKTT120 is an anti-iNKT cell monoclonal antibody that has the potential to rapidly and specifically deplete iNKT cells and, potentially, prevent vaso-occlusion. We conducted an open-label, multi-center, single-ascending-dose study of NKTT120 to determine its pharmacokinetics, pharmacodynamics and safety in steady-state patients with SCD. Doses were escalated in a 3+3 study design over a range from 0.001 mg/kg to 1.0 mg/kg. Twenty-one adults with SCD were administered NKTT120 as part of 7 dose cohorts. Plasma levels of NKTT120 predictably increased with higher doses. Median half-life of NKTT120 was 263 hours. All subjects in the higher dose cohorts (0.1 mg/kg, 0.3 mg/kg, and 1 mg/kg) demonstrated decreased iNKT cells below the lower limit of quantification within 6 hours after infusion, the earliest time point at which they were measured. In those subjects who received the two highest doses of NKTT120 (0.3, 1 mg/kg), iNKT cells were not detectable in the peripheral blood for a range of 2 to 5 months. There were no serious adverse events in the study deemed to be related to NKTT120. In adults with SCD, NKTT120 produced rapid, specific and sustained iNKT cell depletion without any infusional toxicity or attributed serious adverse events. The next step is a trial to determine NKTT120’s ability to decrease rate of vaso-occlusive pain episodes.

Trial Registration: clinicaltrials.gov NCT01783691.

## Introduction

Vaso-occlusion (VO) of post-capillary venules is the predominant cause of morbidity and mortality for patients with sickle cell disease (SCD) [1]. More than just the occlusion of sickle erythrocytes, VO involves multi-cellular interactions between leukocytes, platelets, endothelial cells, as well as normal and sickle-shaped erythrocytes [2]. Pro-inflammatory cytokines promote these interactions through activation of vascular endothelium and leukocytes, along with the recruitment of additional cells to the site of VO [3]. Invariant NKT (iNKT) cells, a cell type known to produce significant amounts of pro-inflammatory cytokines, may be of particular importance in the pathogenesis of VO [3].

Resident in the peripheral blood as well as many organs (liver, spleen, lymph nodes, omentum, lung, eye and kidney), iNKT cells are a unique subset of lymphocytes with characteristics of innate and adaptive immunity [4]. Similar to cells of the adaptive immune system, such as conventional T cells, iNKT cells are activated by T cell receptor (TCR) engagement of antigens displayed on antigen presenting cells (APCs). Unlike conventional T cells, which express a broad TCR repertoire and recognize specific pathogenic proteins, the TCR of iNKT cells is invariant (Vα24Jα18Vβ11) and recognizes the non-specific pattern of glycolipids presented by CD1d, an MHC class 1-like molecule expressed on antigen presenting cells [5]. Although these glycolipids may be derived from microbes, in the pathogenesis of SCD they are likely endogenous [6]. One mechanism is thought to involve danger-associated molecular patterns (DAMPs), which may be generated during VO and can activate toll-like receptors on APCs to synthesize and present glycolipids to iNKT cells [6]. Another potential mechanism of iNKT cell activation in SCD is through interactions between secretory phospholipase A2 (sPLA2), a lipid elevated in the plasma of patients with SCD, and phosphotidylserine (PS), a lipid abnormally exposed on the outer membrane of sickle erythrocytes [7]. Elevated sPLA2 in the plasma of patients with SCD may localize to PS on sickle erythrocytes and generate iNKT cell-activating phospholipids [8–11]. Regardless of the mechanism, once activated, iNKT cells promptly secrete cytokines (interferon-gamma (IFN-γ), interleukin-4 (IL-4) and others) that can activate downstream effector cells and vascular endothelium as well as proteolytic enzymes, such as perforin and granzymes, which can produce tissue injury [12]. This rapid, non-specific activation, akin to the activation of innate immune cells, enables iNKT cells to instigate and sustain a broad inflammatory response that is characteristic of SCD and critical to pathogenesis of VO.

Evidence for a role of iNKT cells in VO comes from mice and patients with SCD, both of which demonstrate a higher percentage of activated iNKT cells in the tissues or peripheral blood compared to controls [13]. In mice, an accumulation of iNKT cells has also been observed in target organs, particularly the lung, with further increases noted after VO. Interruption of iNKT cell activity in mouse models of SCD with an anti-CD1d antibody, an anti-iNKT cell monoclonal antibody, or an adenosine A_2A_ receptor (A_2A_R) agonist prevents VO-induced lung inflammation and injury [13–16]. Based on these preliminary data, we performed a phase 1 study of the A_2A_R agonist, regadenoson, in 27 adults with SCD. Regadenoson, administered as a 24-hour infusion during VO, was shown to decrease iNKT cell activity by 50% [17]. A phase 2 randomized-controlled trial of regadenoson to determine its efficacy for the treatment of a VO crisis is underway [3]. There are limitations, however, to the regadenoson approach. First, regadenoson’s short half-life necessitates an infusion to treat an acute VO event, which is far less optimal than the prevention of one. Second, regadenoson was only shown to decrease iNKT activity by 50%. The iNKT cell activity that remains may still contribute to VO.

NKTT120 is a humanized IgG1κ monoclonal antibody targeted to the Vα24-Jα18 gene-rearranged invariant TCR that has the potential to rapidly and specifically deplete iNKT cells [18]. Prior to a phase 2/3 clinical trial of this promising agent, we conducted a study to determine its pharmacokinetics, pharmacodynamics and safety. Our objective was to identify an appropriate dose of NKTT120 that safely depletes iNKT cells for 3–6 months. The ultimate goal of iNKT cell depletion would be a reduction in the systemic inflammatory state that is characteristic of SCD in order to prevent or decrease the severity of VO and, ultimately, preserve organ function.

## Materials and methods

This study was approved by the Human Research Protection Program at Medical College of Wisconsin, the Human Research Protection Office at Washington University in St. Louis, the IRB and the Office of Human Research Ethics at the University of North Carolina, and the Institutional Review Board at Oakland Children’s Hospital. Written consent was obtained from all subjects prior to participation and all study activities were performed according to the principles expressed in the Declaration of Helsinki. The study was registered on clinicaltrials.gov (NCT01783691).

### Eligibility

Eligible subjects were adults, aged 18 to 60 years, with HbSS/HbSβ-thalassemia^0^, who were at steady-state in the month prior to enrollment without pain events, transfusions, changes in medications or evidence of active infection. All subjects were required to be up to date with CDC-recommended immunizations for asplenic patients. Key exclusion criteria included chronic transfusion therapy, >10 hospital admissions for pain in the year prior to enrollment, asthma, pregnant or nursing, and a history of stem cell transplant. After enrollment, two iNKT cell measurements were obtained (at -4 and -2 weeks) to ensure that subjects had detectable iNKT cell levels before dosing. In order to be eligible, both measurements were required to be above the lower level of quantification (LLOQ).

### Study design

In this study, steady-state adult subjects with SCD were intravenously dosed with NKTT120 over ten minutes without premedication. Doses were escalated in over a range of 0.001 mg/kg to 1.0 mg/kg (0.001, 0.003, 0.01, 0.03, 0.10, 0.3, 1.0), based on estimates from prior studies of NKTT120 in cynomolgus monkeys, using a 3+3 design [18]. A 3+3 design examines 3 subjects per dose cohort, sequentially. If none experience a dose-limiting toxicity (DLT), the dose is considered safe and dose escalation may occur. If 1/3 subjects experiences DLT, 3 additional subjects are examined, and, if more than 1 subject experiences a DLT, further dose escalation is not pursued. The highest dose with ≤ 1 subject experiencing a DLT was defined as the maximum tolerated dose (MTD). Doses were increased until a MTD, or a dose that depleted iNKT cells for 3 to 6 months, was reached. The latter would then be the recommended dose level (RDL) for the phase 2 trial. Notably, in the initial study design, there were 5 doses (0.001–0.10 mg/kg). However, since the MTD was not achieved at 0.10 mg/kg, and some subjects recovered iNKT cell prior to 30 days after dosing, two additional dose cohorts were added (0.30 and 1.0 mg/kg). Subjects were monitored for 6 hours post-infusion and safety laboratory tests were obtained at 6 hours, day +1, day +2, day +7, day +14 and then monthly. Physical examination was performed on day +7, day +14 and then monthly. All subjects were followed for at least 2 weeks or until iNKT cells were detectable in circulation.

### Laboratory methods

Blood samples were collected for safety labs (CBC, reticulocyte count, chemistries, hepatic function), as well as pharmacokinetic and pharmacodynamic analyses, which, in addition to iNKT cell measurement, included an assessment of B and T cells by FACS. Pharmacokinetic samples were stored as serum and frozen at -70°C prior to analysis.

### Pharmacokinetic assay

Pharmacokinetic analyses were performed with a validated capture assay at MPI Research (Mattawan, MI). A rabbit polyclonal anti-NKTT120 IgG was used as both the capture and detection antibody; the detection antibody was also labelled with biotin. Dilutions were then performed to generate a calibration curve after which optical density was measured at 450 nm. Data was analyzed with SOFTmax Pro GxP Version 5.3 (Molecular Devices, Inc, Sunnyvale, California), Watson LIMS Version 7.4 (Thermo Scientific, Philadelphia, Pennsylvania) and ExyLIMS Version 3.0 (MPI Research, Mattawan, Michigan).

#### FACS analyses

FACS analyses were performed at the BloodCenter of Wisconsin (Milwaukee, WI). Red blood cells were lysed and analyzed as two panels on a FACSCanto^™^, which is capable of analyzing 6 colors. The first panel differentiated NK cells, B cells, and T cells and identified CD4+/CD8+ positive subsets with the antibodies: CD3-PE.Cy7, CD20-APC, CD56-PE, CD4-PerCPCy5.5, and CD8-FITC. The second panel identified iNKT cells and activated subsets with the antibodies: CD3-PE.Cy7, CD20-APC, CD4-PerCPCy5.5, CD69-APC.Cy7, Vα24-PE, and Vβ11-FITC. Controls were also run with the antibodies: CD3-PE.Cy7, CD20-APC, CD4-PerCPCy5.5, IgG-APC.Cy7, Va24-PE, and Vb11-FITC, and were used to determine CD69 cutoff. A minimum of 200,000 CD3+ events were acquired. The limit of sensitivity for iNKT cell detection was 0.01% of CD3+ T cells. See S1 Fig for gating strategy.

### Outcome measures

#### Pharmacokinetics

Pharmacokinetics of NKTT120 was determined by plasma measurements of NKTT120 pre- and post-dose at 15 minutes, 30 minutes, 1 hour, 3 hours, 6 hours, day +1, day +2, day +3, day +7, day +14, and at each monthly visit.

#### Pharmacodynamics

iNKT cell depletion and recovery was assessed after dosing with NKTT120 at 6 hours, day +1, day +2, day +7, day +14, and at each monthly visit. Recovery was defined as return at or above the LLOQ of 0.01%.

#### Safety

Adverse events of interest were defined as: 1) any adverse event that occurred within 2 weeks of dosing with the exception of VO pain episodes, 2) any adverse event grade 3 or higher on NCI CTCAE version 4.03, especially study-specific events: cytopenias, infections and cytokine storm, 3) sickle cell-specific adverse events (hemolysis, VOC within 6 hours or increased pain within 72 hours of dose). All events of interest were considered potential DLTs and were reviewed by a scientific review committee that determined attribution of the event to NKTT120. A DLT was an event of interest attributed to NKTT120.

### Data analysis

#### Sample size

Sample size was determined by the 3+3 study design and the number of dose-level cohorts (up to 5 to 6 cohorts were initially planned; when MTD was not reached, additional cohorts were added). Between 2 and 30 subjects were expected to be dosed in the trial, with 3 to 6 subjects per cohort. In addition, a dose-level cohort that represented the MTD was to be expanded to 6 subjects to confirm MTD.

### Analysis of outcomes

Descriptive statistics of the pharmacokinetic and pharmacodynamic data was provided. Differences between lower and upper dose cohorts of NKTT120 was measured with Chi-Square or Fisher’s exact tests for categorical variables and Student’s t or Mann-Whitney U tests for continuous variables that were normally or non-normally distributed, respectively. When appropriate, non-normally distributed variables were log transformed. Spearman’s correlation was used to determine the relationship between baseline iNKT cell values. Mann-Whitney U test was used to compare iNKT cell number between those with and without hydroxyurea.

## Results

### Subjects

**Fig 1:**
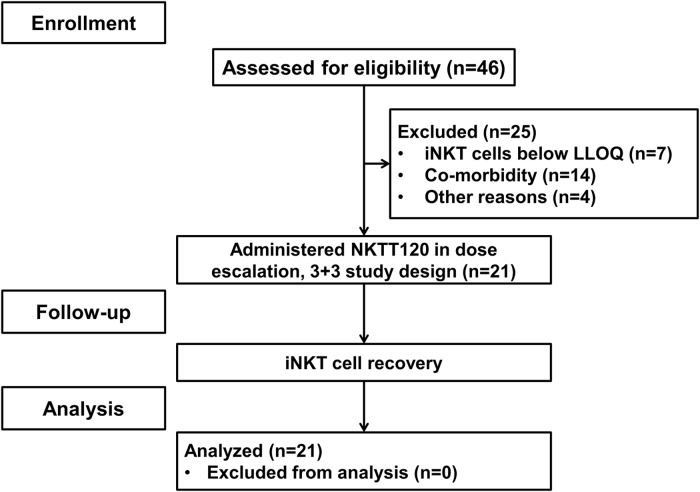
Subject flow through study.

A total of 21 subjects were enrolled into 7 cohorts ranging in dose levels between 0.001 mg/kg and 1.0 mg/kg (Fig 1, Table 1). Approximately half (n = 11) of the subjects had a history of acute chest syndrome and fewer had the co-morbid conditions of stroke, avascular necrosis, gall bladder disease or splenic sequestration. Fourteen of the subjects (67%) were treated with hydroxyurea and 13 subjects (62%) had reported one or more VO pain episode that required contact with a medical facility in the 12 months prior to study start.

**Table 1:** Demographics, sickle cell disease characteristics and morbidities.

	All dose cohorts (n = 21)	Lower dose cohorts (n = 12)	Higher dose cohorts (n = 9)	*P*
**Demographics**				
Age (years), median (IQR)	26 (10)	24 (14)	30 (10)	NS
Gender, % female	38	50	22	NS
**SCD characteristics**				
Hemoglobin (g/dL), mean (SD)	9.0 (1.5)	9.3 (1.4)	8.7 (1.6)	NS
Recticulocyte count (10^6^/μL), mean (SD)	0.26 (0.12)	0.20 (0.07)	0.33 (0.12)	**0.005**
Mean cellular volume (fL), mean (SD)	97.9 (10.5)	98.8	96.7	NS
WBC (k/uL), mean (SD)	8.4 (2.9)	6.8 (2.3)	10.5 (2.3)	**0.002**
LDH (U/L), mean (SD)	533.4 (313.1)	624 (376)	413 (146)	NS
CRP (mg/L), mean (SD)	5.3 (4.8)	5.2 (5.2)	5.4 (4.5)	NS
Hydroxyurea, % yes	67	83	44	NS
**SCD morbidities**				
Pain episode in past year, % yes	62	33	89	NS
# pain episodes in past year, mean (SD)	1.1 (1.2)	0.83 (1.4)	1.56 (1.0)	NS
ACS, % yes	52	42	67	NS
# ACS lifetime, mean (SD)	1.2 (1.6)	1.17 (1.9)	1.33 (1.2)	NS
Stroke, % yes	5	8	0	NS
AVN, % yes	24	8	44	NS
Gallbladder disease, % yes	38	42	33	NS
Splenic sequestration, % yes	19	17	22	NS
# cumulative morbidities*, mean (SD)	0.9 (1.0)	0.6 (0.9)	1.2 (1.0)	NS

Definitions: ACS = acute chest syndrome, AVN = avascular necrosis, CRP = c-reactive protein, IQR = interquartile range, LDH = lactate dehydrogenase, SD = standard deviation, WBC = white blood count.

*cumulative morbidities = ACS + stroke + AVN + gallbladder disease + splenic sequestration.

When the recent histories of those in the lower dose cohorts were compared to those in the higher dose cohorts, there were significantly higher white blood cell and reticulocyte counts in the subjects assigned the higher dose cohort (Table 1). There were also more pain episodes in the past year in the higher dose cohorts compared to the lower dose cohorts, as well as a higher prevalence of avascular necrosis and cumulative number of morbidities (these did not achieve statistical significance). Although hydroxyurea use was not statistically different between those in the lower and higher dose cohorts, its use was not equally distributed across dose cohorts. All subjects in dose cohorts 1, 3, 4, and 6 were prescribed hydroxyurea, as opposed to 1 of 3 subjects in dose cohort 2 and 7, and no subjects in dose cohort 5. The small numbers in each dose cohort make it difficult to determine the effect of hydroxyurea, if any, on iNKT cell recovery after dosing.

### Pharmacokinetics

**Fig 2:**
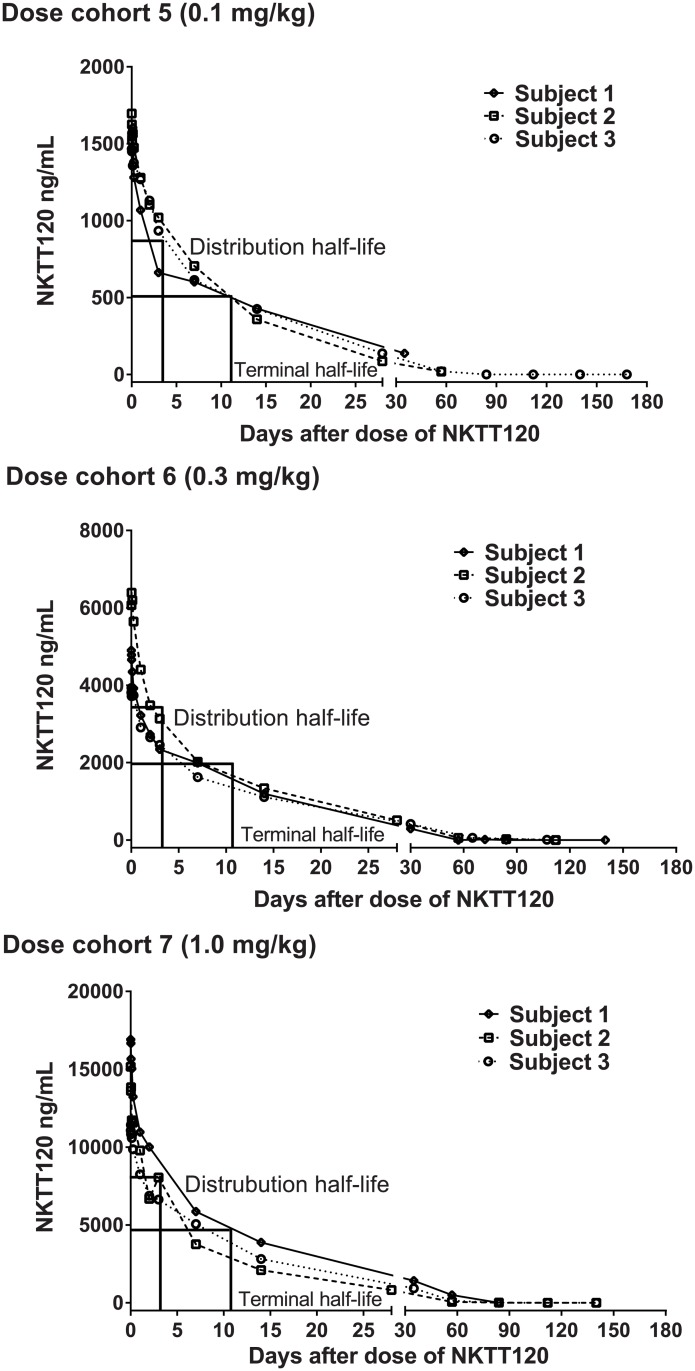
Median NKTT120 serum concentrations over time by dose cohort. Shown are the higher dose cohorts 5 (0.1 mg/kg), 6 (0.3 mg/kg) and 7 (1.0 mg/kg).

After a single intravenous dose of NKTT120, the maximum plasma concentration and area under the curve increased in a dose-proportional manner (Fig 2). NKTT120 was eliminated from serum in a biphasic manner, with a relatively short distribution phase (about 3 days) and median terminal half-lives of 263 hours (range: 98–386 hours).

### Pharmacodynamics

**Fig 3:**
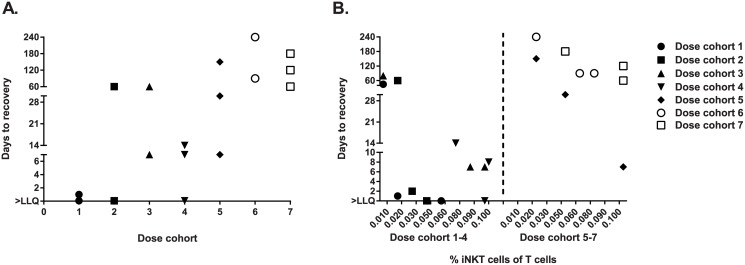
Invariant NKT cell percent of T cells over time. **(A)** After NKTT120, days to iNKT cell recovery above the lower limit of quantification (LLQ) generally increases across 7 dose cohorts, (**B)** Dose cohorts 1–4 and 5–7 by pre-drug iNKT cell levels expressed as percent of T cells. Lower pre-drug iNKT cell level is associated with longer recovery time, especially in dose cohorts 1–4.

In the two pre-drug measures, iNKT cell levels within a subject were highly correlated (r = 0.9, P<0.01). Pre-drug iNKT cell numbers were not affected by hydroxyurea use (median pre-drug iNKT cell number for those who used hydroxurea was 0.05% T cells compared to 0.045% T cells for those who did not, P = 0.3). After a dose of NKTT120, all subjects’ iNKT cell levels decreased, although the degree and duration of the decrease varied with the dose administered (Fig 3A). Subjects with higher baseline iNKT cell levels were less likely to deplete iNKT cells after a given dose, especially in lower dose cohorts, and experienced re-appearance of iNKT cells in the peripheral blood occurred more quickly (Fig 3B).

In each of the 4 lower dose cohorts (0.001 mg/kg to 0.03 mg/kg), 2 of 3 subjects’ iNKT cell values dropped below LLOQ within 6 hours. Of these subjects who dropped below LLOQ, iNKT cell recovery varied from 1 day to 2 months. All subjects in dose cohort 5 (0.1 mg/kg), 6 (0.3 mg/kg) and 7 (1 mg/kg) decreased iNKT cells below LLOQ within 6 hours (Fig 3A). One subject in dose cohort 5, however, rose above the LLOQ by day 7. The longest time to iNKT cell recovery above LLOQ was a subject in dose cohort 5 who required 5 months. In dose cohort 6, 2 subjects rose above the LLOQ by month 2, with the third rising briefly to the minimum detectable level at month 4, but then returned below LLOQ from months 5 through 8. Of note, although iNKT cells returned at the LLOQ in month 4, the investigators elected to continue with monthly iNKT cell measurements in the subject since their values were borderline detectable and their re-appearance was later than others in the study (up to that point). In dose cohort 7, subjects recovered at 2, 4 and 5 months after NKTT120 administration.

After recovery of iNKT cells in the peripheral blood, subjects were followed for 1 additional month. At the end-of-study measurement, it was notable that subjects with high starting levels of iNKT cells (>0.03%) usually did not recover to their baseline levels. This suggests that for patients with higher starting levels of iNKT cells more complete depletion may be achieved with subsequent doses.

No evidence was seen for the theoretical concern that NKTT120 could activate iNKT cells. Post-dose cytokine measurements were generally low and showed no indication of iNKT cell activation. There was also no indication of significantly increased iNKT cell activity after recovery. Of the 7 dose cohorts, only 2 subjects from dose cohort 3 and those in dose cohort 5 showed an increase in the percent of CD69+-activated iNKT cells when end-of-study samples were compared to pre-drug samples. These differences were not statistically significant. There were no changes in T or B cell percentages by FACS analysis after treatment with NKTT120 at any dose (data not shown).

### Safety

Subjects were followed for at least 2 weeks after iNKT cell recovery (minimum 2 weeks, maximum 8 months). No DLTs were experienced in 21 subjects. Seventeen subjects (81%) experienced a total of 69 AEs, of which 57 (83%) were grade 1 or 2 (S1 Table). The most frequently reported was VOC and fatigue. Seven subjects (33%) had treatment-related AEs, but none were grade 3 or higher. An increased occurrence of AEs, including treatment-related AEs, was seen in the higher dose cohorts, although this may be expected since the duration of follow-up was significantly longer. Also, patients in the higher dose cohorts may have had more severe or poorly-controlled disease to begin with. There were more VO events on average in the year before study entry among the higher compared to the lower dose cohort (0.8 VO events/year in cohorts 1–4 versus 1.6 VO events/year in cohorts 5–7) and less hydroxyurea use (44% versus 83%). Importantly, regardless of dose cohort, there were no documented infections observed during the course of the study. Cases occurred in which antibiotics were initiated, but no subject completed a course of antibiotics. In all cases antibiotics were stopped per the discretion of the treating physician, likely because no organisms were isolated on microbiology testing. Finally, the formation of auto-antibodies to NKTT120 or otherwise were a theoretical concern, but there were no anti-NKTT120 antibodies detected and no signs autoimmune disease in any subject.

## Discussion

An intravenous bolus of NKTT120 produced rapid, specific and sustained iNKT cell depletion without any infusional toxicity or attributed SAEs. Depletion and recovery of iNKT cells was related to the pre-drug levels in circulation and the dose of NKTT120. At higher doses (0.1, 0.3, 1 mg/kg), all subjects were depleted of iNKT cells within 6 hours, but the length of time they remained depleted varied between subjects. It could not be determined from this study whether iNKT cell depletion with NKTT120 decreased the rate of VO pain episodes. A randomized trial, in which repeated therapeutic doses of NKTT120 are administered with the aim of iNKT cell depletion for long periods, will be required to test NKTT120’s efficacy.

If NKTT120 is to prevent VO, iNKT cells must be reduced in the tissues as well as in the circulation. Peripheral blood iNKT cells represent only a fraction of the total iNKT cell population in humans; the remainder resides outside the circulation in the tissues where most iNKT cell activity occurs [19,20]. Though VO initiates as a vascular event, iNKT cells amplify the crisis at the tissue level. If NKTT120 does not achieve tissue depletion, the target cells will either re-equilibrate into circulation or promote the process of VO through cytokine production from the tissues. In the lower 4 dose cohorts, re-equilibration from tissues likely occurred as there was rapid recovery of circulating iNKT cells. In contrast, the 3 higher doses of NKTT120 produced sustained iNKT cell depletion that lasted beyond the time when NKTT120 could be detected in the peripheral blood. Here, the target cells were likely depleted in the tissues as well as the circulation, in which case recovery is a function of the drug’s half-life and the rate of regeneration for iNKT cells.

iNKT cells are continuously regenerated in the thymus in a unique developmental process that differs from conventional T cells [21]. Unlike conventional T cells, which mature and acquire a memory phenotype after exposure to foreign antigens, iNKT cells are constantly regenerated in the thymus by recognition of endogenous antigens, without the requirement for prior exposure to a foreign antigen [22]. Thus, after depletion with NKTT120, the population of iNKT cells would be expected to completely reconstitute with the same function as before treatment. In fact, we demonstrated the normal function of iNKT cells upon return after depletion in non-human primate toxicology studies. iNKT cells from 6 animals from the highest NKTT120 dose groups that had recovered iNKT cells (0.1 mg/kg and 0.3 mg/kg, respectively) were compared to iNKT cells in samples from 2 naïve control animals following treatment with an iNKT cell activating glycolipid, α-galactosylcerimide. iNKT cells were activated to the same degree in recovered animals and control animals, as reflected by up-regulation of the early activation marker CD69 (*unpublished observation*).

In contrast, if a clonally-expanded population of memory T cells is pharmacologically depleted, they can only recover when new emigrants from the thymus, with identically rearranged TCRs, are exposed to similar or identical pathologic antigen as the original insult. This clonal loss or diminution of memory T cells, a well-known complication of pan-lymphocyte or T cell-depleting antibodies, can leave holes in immune systems and patients susceptible to infections. NKTT120, on the other hand, could be dosed repeatedly without permanent effects on immunity because no loss of memory phenotype is associated with depletion of iNKT cells. In our study, a dose of 1 mg/kg (dose cohort 7), which depleted all subjects for at least 2 months, would facilitate an every 3 month dose schedule to keep iNKT cells depleted chronically in both the peripheral blood and the tissues. And, because recovery was defined as return of iNKT cells above the LLOQ as opposed to pre-NKTT120 levels, fewer iNKT cells will be present when subjects are re-dosed. Therefore the impact on tissue depletion would be expected to be greater with multiple doses than with a one-time dose.

No SAEs were attributed to NKTT120 as part of this study, but there are theoretical concerns about long-term iNKT cell depletion: cancer, autoimmunity and infection [23–25]. Mice with an absence of iNKT cells have a normal life expectancy and normal fertility. They do not develop cancer, but autoimmune nephritis has been reported [26]. In regards to infection risk, there is no clear evidence to suggest these mice are predisposed to infections. Studies of iNKT cell-deficient mice, either CD1d^-/-^ or Jα18^-/-^, show variable responses when infected with several types of bacteria, as well as fungi, protozoa and viruses [27–30]. Absence of iNKT cells sometimes worsens outcomes and sometimes improves outcomes with no clear relationship to the nature of the pathogen [27–36]. In humans, there are no reports of a patient with a pure deficiency of iNKT cells. The evidence to suggest an increased risk of infections due to iNKT cell deficiency is descriptions of patients with combined deficiencies. One case of disseminated varicella observed after administration of a live vaccine in a patient with an NKT cell deficiency has been reported [37]. In this patient, it is not clear whether the deficiency was restricted to iNKT (Type 1 NKT targeted by NKTT120) cells or included Type 2 NKT (not targeted by NKTT120) cells as well. In addition, patients with a combined defect in NK and NKT cells have been reported to have an increased susceptibility to infections with the human herpes virus family [38]. It is unclear whether the absence of NKT cells contributes to this susceptibility, though, as patients with a pure defect in NK cells are known to be at risk for infections with the human herpes virus family. In patients with cancer and autoimmune disease, lower levels of iNKT cells have been reported, but whether this is a cause or effect of the condition is not known [39–41].

There were limitations to our study. First, NKTT120 was administered to subjects once. Longer term studies will be required to assess the risk of NKTT120 therapy. Two subjects, however, were depleted of iNKT cells for 5 months or greater with no SAEs attributed to NKTT120. Regardless of the study’s duration, the theoretical risks of the drug must be weighed against the real risks of VO: the average life expectancy for an adult with SCD is less than 50 years, largely because of VO [1]. Second, the current study only determined pharmacokinetics, pharmacodynamics and safety. It was not designed to determine the clinical efficacy of NKTT120. There were admissions for VO pain in our study, even among subjects whose iNKT cells were depleted. In fact, subjects in the higher dose cohorts, who were depleted for longer, accounted for the majority of the pain events. However, compared to subjects in the lower dose cohorts, those in the higher dose cohorts were also followed for a significantly longer period of time. Additionally, their disease was likely more poorly-controlled and their phenotype more severe than the lower dose cohorts. Those in higher dose cohorts had less hydroxyurea use, a higher white blood cell and reticulocyte count, a higher historical rate of pain admissions, and a higher prevalence of avascular necrosis and cumulative number of morbidities compared to the those in the lower dose cohorts. Given the differences between subjects and lack of a control group, these events provide little insight into NKTT120’s potential as therapeutic for SCD. NKTT120’s ability to decrease VO and reduce the rate of pain and inflammation will need to be determined in future studies, with appropriate controls, when repeated doses of NKTT120 deplete iNKT cells for a length of time sufficient to capture differences in VO rate. And regardless, no therapy, short of a cure, will likely prevent all episodes of pain. Hydroxyurea, a highly efficacious therapy for SCD, reduces VO pain rate by 50%, a reduction in VO which confers a significant mortality benefit [42,43]. If NKTT120 could demonstrate a reduction in pain events similar to hydroxyurea, patients with SCD would benefit tremendously.

NKTT120 has the potential to impact clinical care for patients with SCD. Through a reduction in inflammation and prevention of VO events, NKTT120 would add to the limited armamentarium of treatments for patients with SCD. Hydroxyurea is currently the only therapy approved to prevent VO, but, unfortunately, it’s ineffective in up to 50% patients, mostly because of the need for daily doses [44]. NKTT120’s dose regimen of every 3 month infusions in the clinic is a major advantage. Providers will be ensured of patient’s receipt of the drug and patients will not burdened by a daily medicine. This study determined that up to a 1 mg/kg dose of NKTT120 could be administered safely with a rapid, yet sustained, effect on iNKT cell depletion. The next step is a randomized, placebo-controlled clinical trial to determine the ability of repeat-dose NKTT120 to decrease the rate of VO pain, as well as determine the risks, if any, to long-term iNKT cell depletion.

## Supporting information

S1 Table Adverse events and attribution to NKTT120 and sickle cell disease. (DOCX)

S1 Fig Gating strategy for identification of invariant NKT cells. Shown are Patient #2 from dose cohort 3 (top panel) and patient #1 from dose cohort 7 (bottom panel). FACS plots for iNKT cell identification are shown pre-drug, at 6 hours post-drug, and at recovery.
(TIF)

S1 Dataset (XLS)

S1 Protocol (PDF)

S1 Trend Checklist (PDF)
