# Supplementary material for: NNKTT120, an anti-iNKT cell monoclonal antibody, produces rapid and sustained iNKT cell depletion in adults with sickle cell disease
Source: PLoS One. 2017 Feb 2;12(2):e0171067. doi: 10.1371/journal.pone.0171067 (PMC5289534; doi:10.1371/journal.pone.0171067)
Supplement: S1 Table — (DOCX) [file pone.0171067.s001.docx]

**Supplemental table. Adverse events and attribution to NKTT120 and sickle cell disease**

|  | **All dose cohorts (n=21)** | **Lower dose cohorts (n=12)** | **Higher dose cohorts (n=9)** |
| --- | --- | --- | --- |
| **Adverse events** |  |  |  |
| # reported | 69 | 24 | 45 |
| # related to NKTT120 | 15 | 11 | 4 |
| fatigue | 5 | 3 | 2 |
| asthenia | 1 | 1 | 0 |
| chest pain | 1 | 1 | 0 |
| insomnia | 1 | 1 | 0 |
| decreased libido | 1 | 1 | 0 |
| neutropenia | 1 | 1 | 0 |
| abdominal pain/diarrhea | 2 | 0 | 2 |
| dizziness | 1 | 1 | 0 |
| throat irritation | 1 | 1 | 0 |
| pruritus | 1 | 1 | 0 |
| # related to SCD | 13 | 5 | 8 |
| pain crisis | 10 | 4 | 6 |
| acute chest syndrome | 2 | 1 | 1 |
| pulmonary embolus | 1 | 0 | 1 |
| # grade 3+ reported | 12 | 4 | 8 |
| # grade 3+ related to NKTT120 | 0 | 0 | 0 |
| # grade 3+ related to SCD | 12 | 4 | 8 |
| **Serious adverse events** |  |  |  |
| # reported | 12 | 4 | 8 |
| # related to NKTT120 | 0 | 0 | 0 |
| # related to SCD | 12 | 4 | 8 |
| # grade 3+ reported | 12 | 4 | 8 |
| # grade 3+ related to NKTT120 | 0 | 0 | 0 |
| # grade 3+ related to SCD | 12 | 4 | 8 |
| pain crisis | 9 | 3 | 6 |
| acute chest syndrome | 2 | 1 | 1 |
| pulmonary embolus | 1 | 0 | 1 |
