# Supplementary figures and images for: NNKTT120, an anti-iNKT cell monoclonal antibody, produces rapid and sustained iNKT cell depletion in adults with sickle cell disease
Source: PLoS One. 2017 Feb 2;12(2):e0171067. doi: 10.1371/journal.pone.0171067 (PMC5289534; doi:10.1371/journal.pone.0171067)

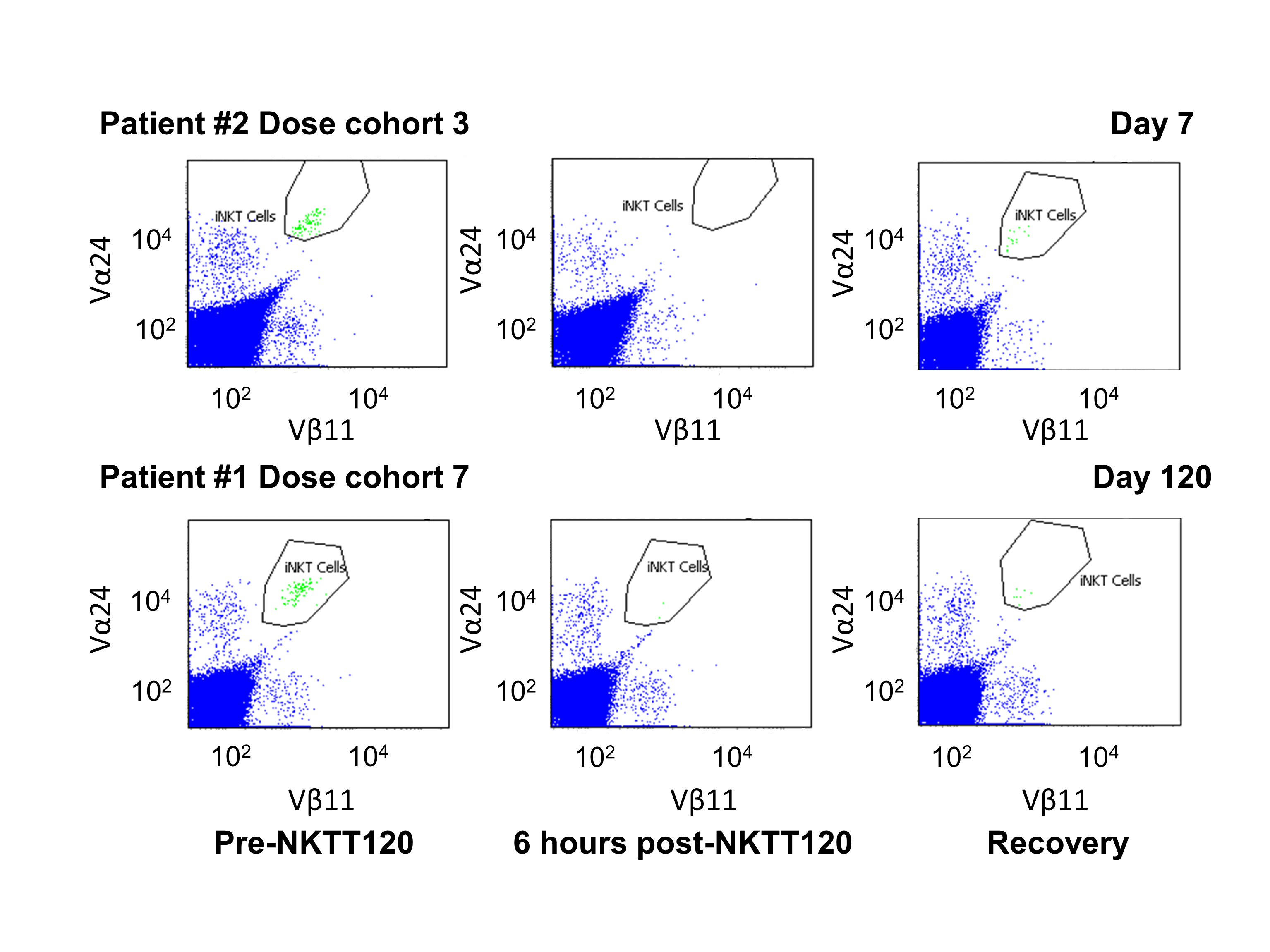

Supplement: S1 Fig — Shown are Patient #2 from dose cohort 3 (top panel) and patient #1 from dose cohort 7 (bottom panel). FACS plots for iNKT cell identification are shown pre-drug, at 6 hours post-drug, and at recovery. (TIF) [file pone.0171067.s002.tif]
