# Supplementary material for: NNKTT120, an anti-iNKT cell monoclonal antibody, produces rapid and sustained iNKT cell depletion in adults with sickle cell disease
Source: PLoS One. 2017 Feb 2;12(2):e0171067. doi: 10.1371/journal.pone.0171067 (PMC5289534; doi:10.1371/journal.pone.0171067)
Supplement: S1 Protocol — (PDF) [file pone.0171067.s004.pdf]

PROTOCOL NUMBER: 120-SCD1

A Single Ascending Dose (SAD) Phase I Study to Evaluate the Safety, Pharmacokinetics, Pharmacodynamics and Biologic Activity of Intravenous (IV) NKTT120 in Adults with Stable Sickle Cell Disease (SCD)

This is an open-label study.

Investigational product:
NKTT120

Original Protocol :
13 November 2012

Sponsor:
NKT Therapeutics Inc.
880 Winter St., Suite 350
Waltham, MA 02451

Amendment: Date:

CRO:
Novella Clinical, Inc.
4309 Emperor Boulevard, Suite 400
Durham, NC 27703

Principal Investigator:
Joshua Field, MD, MS
Blood Center of Wisconsin
8727 Watertown Plank Road
Milwaukee, WI 53226-3548
Mail to P.O. Box 2178
Milwaukee, WI 53201-2178
Tel: 414-937-3848
Fax: 414-937-6811
Email: Joshua.field@bcw.edu

Protocol Chair:
David Nathan, MD
Dana Farber Cancer Institute
450 Brookline Avenue
Boston, MA 02215-5450
Tel: 617- 632-2155
Fax: 617-632-4367
Email: David.nathan@dfci.harvard.edu

This document contains confidential information belonging to NKT Therapeutics, Inc. (NKTT). Except as otherwise agreed to in writing, by accepting or reviewing this document, you agree to hold this information in confidence and not copy or disclose it to others (except where required by applicable law) or use it for unauthorized purposes. In the event of any actual or suspected breach of this obligation, NKTT must be promptly notified.

INVESTIGATOR'S AGREEMENT

I have received and read the Investigator's Brochure for NKTT120. I have read the 120-SCD1 protocol and agree to conduct the study as outlined. I agree to maintain the confidentiality of all information received or developed in connection with this protocol.

____

Printed Name of Investigator

____

Signature of Investigator

____

Date (dd/mmm/yyyy)

PROCEDURES IN CASE OF EMERGENCY

Table 1: Emergency Contact Information

Role in Study	Name	Address and Telephone number
NKT Therapeutics responsible Physician	Rosemary Mazanet, MD, PhD	NKT Therapeutics Inc.<br>880 Winter St., Suite 350<br>Waltham, MA 02451<br>Phone: (781) 684-0290
Novella Medical Monitor	Leslie Clapp, MD	Novella Clinical, Inc.<br>4309 Emperor Blvd.,<br>Suite 400<br>Durham, NC 27703<br>Office: (919) 972-2447
Novella Clinical Lead Safety Specialist	Laura DeVantier	Novella Clinical, Inc.<br>4309 Emperor Blvd.,<br>Suite 400<br>Durham, NC 27703<br>866-758-2798
24-Hour Emergency Contact for Safety Medical Monitor<br><br>**SAFETY HOTLINE NOVELLA 24 hour Call: 866-758-2798**	Leslie Clapp, MD	Novella Clinical, Inc.<br>4309 Emperor Blvd.,<br>Suite 400<br>Durham, NC 27703<br>Office: (919) 972-2447

1. SYNOPSIS

**Name of Sponsor/Company:**<br>NKT Therapeutics Inc.<br>880 Winter St., Suite 350<br>Waltham, MA 02451<br>Phone: (781) 684-0290	
**Name of Investigational Product:**<br>NKTT120	
**Name of Active Ingredient:**<br>NKTT120	
**Title of Study:**<br>A Single Ascending Dose (SAD) Phase I Study to Evaluate the Safety, Pharmacokinetics, Pharmacodynamics and Biologic Activity of Intravenous (IV) NKTT120 in Adults with Stable Sickle Cell Disease (SCD)	
**Principal Investigator:**<br>Joshua Field, MD, MS<br>Blood Center Wisconsin, Milwaukee Wisconsin	
**Studied period (years):**<br>1.5 years<br>Estimated date first subject enrolled: 2/1/2013<br>Estimated date last subject completed: 8/1/2014	**Phase of development:**<br>1b
Objectives:<br>**Primary:**<br>To assess the safety and maximum tolerated dose (MTD) level of a SAD of IV NKTT120 in adults with stable SCD. If no MTD is recognized at the dose levels planned, to determine the recommended dose level for Phase 2 studies.<br>**Secondary:**<br>The secondary objectives of the study include determination of pharmacokinetics (PK) and pharmacodynamics (PD), the assessment of biological activity, the measurement of various biomarkers and determination of the presence or absence of anti-drug antibodies (ADAs).	
**Methodology:**<br>This first-in-human Phase 1 study will evaluate safety, tolerability, pharmacokinetic characteristics of the antibody, the pharmacodynamic profile of iNKT cell depletion and recovery, markers of SCD activity and biomarkers of inflammation, coagulation, and endothelial cell activation.<br>This is an open-label, multi-center, single ascending dose Phase 1 study aimed at determining the tolerability (maximum tolerated dose [MTD] or recommended dose level [RDL] for Phase 2) of IV NKTT120 in adult subjects with stable SCD. For the purpose of this protocol, “stable” SCD is defined as no acute painful vaso-occlusive crisis (pVOC) or acute chest syndrome (ACS) requiring	

hospitalization or emergency department (ED)/acute outpatient management within one month prior to dosing (per Exclusion Criteria). In addition to determining the safety profile of NKTT120 in subjects with stable SCD, this study will also examine clinical markers of SCD activity, daily pain, analgesic usage, airway hyperreactivity (AHR) and QoL as secondary outcomes. During the 2-week screening run-in period and throughout the follow-up, subjects will keep a daily electronic diary (eDiary) for pain, analgesic use and shortness of breath. The QoL assessments will be piloted in this Phase 1 study to determine their suitability as potential efficacy endpoints in future clinical studies.

Number of subjects (planned):

30

Diagnosis and main criteria for inclusion/exclusion:

Subjects must meet the following for inclusion in the study:

- Adults 18-50 years of age
- Willing and able to sign informed consent
- Diagnosed with SCD, either homozygous for hemoglobin (Hb) S genes (HbSS) or heterozygous for HbS and β thalassemia genes (HbS/β^0) based on hemoglobin analysis
- Have measurable baseline iNKT cells determined by recognition on FACS assay of circulating peripheral blood iNKT cells measured at screening pre-run-in visit and again at the screening-run-in visit
- Have stable SCD defined as not having experienced acute pVOC, ACS or other major or significant SCD-associated event requiring hospitalization or outpatient medical care during the month prior to enrollment.

Subjects will be excluded from the study if they meet any of the following exclusion criteria:

- SCD-related pVOC or ACS that required hospitalization or treatment in an emergency or acute care outpatient setting ≤1 month prior to enrollment
- Require a program of prescheduled regularly administered chronic blood transfusion therapy, or be expected to receive a transfusion during enrollment on the study. Subjects are not eligible if they have received a packed red blood cell (PRBC) transfusion within 4 weeks of enrollment.
- Experienced more than ten painful crises for which medical treatment was received in the twelve months prior to enrollment
- Change in medications or doses of medications prescribed for SCD (e.g. hydroxyurea, oral antibiotic prophylaxis, pain medications) or medications prescribed for concomitant medical conditions (e.g. antihypertensives, medications to control hypercholesterolemia, vitamin D, calcium supplements, birth control, short acting $beta_2$ agonist (SABA), medications to control neuropathic pain) ≤ 2 months prior to enrollment
- Moderate-to-severe asthma or taking asthma medications other than inhaled SABA (i.e., step 2 or higher level asthma medications) to relieve symptoms

- Use of corticosteroids (oral, parenteral, or inhaled) > 10 mg/day prednisone equivalent ≤ 2 months prior to enrollment
- Hematology values outside of protocol-defined ranges at screening, including:
  - Hemoglobin ≥ 6 g/dL
  - Platelets ≤ 150 x 10^9/L
  - Lymphocytes < 1.0 x 10^9/L
  - Absolute neutrophils ≤ 2 x 10^9/L
  - PT or aPTT outside of normal range
- Serum chemistry and liver function test values outside of protocol-defined ranges at screening, including:
  - ALT (SGPT) ≥ 2.5 x ULN
- Serum creatinine ≥ 1.5 mg/dL and spot urine protein/creatinine ratio ≤ 0.5 mg/mg
- Evidence of current infection requiring ongoing treatment
- Evidence of latent or active mycobacterium tuberculosis (TB) determined by positive QuantiFERON-Gold test or screening chest radiograph (CXR), or lack of documentation of adequate treatment of prior infection with mycobacterium tuberculosis (TB) or history of infection with atypical mycobacterium
- History of viral infection(s) requiring ongoing treatment (e.g., HIV, HCV, HBV, HSV type 2)
- Received any live or attenuated viral immunization within six months prior to enrollment
- Not up-to-date on CDC-recommended immunizations for adults (see section 18.1) or received immunization within one month prior to enrollment
- Major concurrent illness or medical condition that in the opinion of the investigator would preclude participation in a clinical study, including but not limited to:
  - Uncontrolled significant cardiovascular disease or cardiac arrhythmia or clinically relevant abnormal screening electrocardiogram (ECG)
  - Serious event such as stroke, transient ischemic attack (TIA), deep venous thrombosis (DVT), or pulmonary embolism (PE) ≤ 6 months prior to enrollment
  - Significant bleeding of any cause ≤ 6 months prior to enrollment
  - Psychiatric, behavioral, or addictive disorder that in the opinion of the investigator would preclude participation in a clinical study
  - Known severe central nervous system (CNS) vasculopathy
  - Interstitial lung disease requiring continuous oxygen or clinically significant abnormal screening chest x-ray (CXR), or oxygen (O_2) saturation < 90% on room air
  - Known severe pulmonary hypertension (tricuspid regurgitent jet velocity (TRV) ≥ 3.0

m/sec on 2-D echocardiogram or an estimated pulmonary artery systolic pressure ≥ 40 mmHg

- Type 1 or type 2 diabetes
- History of cancer
- Known allergy or hypersensitivity to any component of NKTT120 manufacturing or formulation or has previously experienced adverse reaction to protein or biologic therapeutic or experimental agent
- Pregnant or nursing
- Subjects of either gender who are not using highly effective method of contraception while on study
- Subject has participated in a clinical trial and received an investigational product within one month, five half-lives or twice the duration of the biochemical or biological effect (whichever is longer) of the investigational product prior to the start of screening for this study
- History of marrow or stem cell transplant

Investigational product, dosage and mode of administration:

Escalating single IV doses of NKTT120. Five dose cohorts beginning at 0.001 mg/kg will be administered. The planned doses are 0.001 mg/kg, 0.003 mg/kg, 0.01 mg/kg, 0.03 mg/kg, and 0.1 mg/kg.

As the study progresses, the current dose escalation plan of approximately half log increment may be reduced to a slower arithmetic increment without increasing the number of maximum subjects. This decision will be made based on observed iKNT cell recovery times in the lower dose cohorts. Similarly, a dose de-escalation using 3+3 enrollment will be applied if indicated by the data and recommended by the Clinical Investigators and/or Safety Review Committee.

Duration of treatment:

Subjects will be followed for 30 days after their iNKT cells have recovered to the level of detection, at which time they have their End of Study (EOS) visit.

Reference therapy, dosage and mode of administration:

There is no active comparator in this study.

Criteria for evaluation:

Pharmacokinetics: The PK objective is to determine the PK profile of NKTT120, the relationship of PK to depletion and recovery of iNKT cells, the adverse event (AE) profile and presence of ADAs.

Pharmacodynamics: The PD objective is to determine the profile of iNKT cell depletion and recovery after dosing with NKTT120 and to correlate the PK and PD profiles. It is anticipated that iNKT cells will recover after NKTT120 is eliminated from or is present at very low concentrations in the serum.

Safety:

Only one subject will be dosed on any day. Subjects will be monitored in the clinical trial center

immediately before and for at least 6 hours after each dose for acute toxicity (i.e., infusion reactions or cytokine release syndrome). Subjects will not be discharged from the clinical trials center if they are not stable at 6 hours post-dosing. Subjects will return for follow-up safety monitoring and to have blood drawn for PK, hematology and chemistry assessments per the protocol schedule of evaluations (Table 3). These PK and PD assessments require frequent monitoring until recovery of iNKT cells (Table 3). Monthly monitoring will include: assessment of AEs, change in medications, symptom-directed physical examinations, vital signs, medication review, hematology profile, lymphocyte subsets (including iNKT cells and their activation state), chemistry profile, liver function tests, prothrombin time (PT), and activated partial thromboplastin time (aPTT) (Table 3). Subjects will have blood drawn for analysis of iNKT cell numbers monthly until recovery. Adverse events will be graded for severity using the CTCAE v.4.03 with modifications for SCD and presented by body system and relationship to NKTT120.<br><br>The Safety Review Committee (SRC) will immediately review all adverse Events of Interest (EOI) that might be dose limiting toxicities (DLT) to determine if they do, in fact constitute a DLT. The SRC will also review serious adverse events (SAEs) reported for all subjects in the study, as well as all safety and laboratory data at regular intervals to assess emergent safety signals during longer-term follow-up post-dosing.
**Statistical methods:**<br><br>Primary outcome: Maximum tolerated dose (MTD) is defined as the dose level that is estimated to produce DLT in < 33% of subjects in a cohort using a 3+3 design. If no MTD is achieved, the RDL will be determined based on the overall safety profile of NKTT120 and the duration of iNKT cell depletion.<br><br>Secondary outcome: Non-compartmental pharmacokinetic parameters--peak concentration (C_{max}), minimum concentration (C_{min}), average concentration (C_{avg}), area under the time-concentration curve (AUC), half-life ($t_{1/2}$), volume of distribution (V_d)--will be calculated for all subjects. Descriptive statistics of these parameters will be provided by dose level.<br><br>Descriptive statistics of the PK/PD (peripheral iNKT cell subset/T cell (TC) ratio depletion and recovery to quantifiable levels) will be provided by dose level. PK/PD will be examined for association with AEs, pain scores and other endpoints, and changes in biomarkers.<br><br>Demographics and baseline status will be documented by dose level and/or overall, as applicable.<br><br>Descriptive statistics of change in pain score, analgesic use, peak expiratory flow, and shortness of breath will be collected by eDiary and/or during clinic visits. The Quality of Life (QoL) assessments will be collected at clinic visits and will be compared to pre-treatment screening run-in values. Trends in improvement or deterioration in these values with treatment and across the dose-level cohorts will be described.<br><br>Descriptive statistics of change from screening in serum and cellular biomarkers of inflammation, coagulation, and endothelial function will be provided.<br><br>ADAs will be described as present or absent and correlated with NKTT120 serum concentrations and iNKT cell recovery. Numbers of subjects with positive tests for ADAs per cohort and per study will be reported.

2. TABLE OF CONTENTS AND LIST OF TABLES AND FIGURES

TABLE OF CONTENTS

1. SYNOPSIS 4
2. TABLE OF CONTENTS AND LIST OF TABLES AND FIGURES 9
3. LIST OF ABBREVIATIONS AND DEFINITIONS OF TERMS 14
4. INTRODUCTION 19
4.1. NKTT120 19
4.2. Description of invariant NKT cells 19
4.3. Sickle cell disease and invariant NKT cells in vaso-occlusion in SCD 19
4.4. Nonclinical studies with NKTT120 21
4.5. Clinical Studies 22
4.6. Potential risks of NKTT120 22
4.6.1. Cytokine Release Syndrome (CRS) 22
4.6.2. Depletion of iNKT cells 24
4.6.2.1. Bacterial infections 24
4.6.2.2. Increased susceptibility to viral infections 25
4.6.2.3. Cancer 26
4.6.2.4. Enhanced autoimmunity 26
4.6.2.5. Fertility and embryogenesis 26
4.6.3. Potential risk specific to adults with SCD and risk mitigation 26
4.7. Subject population and study rationale 27
4.8. Dose Rationale 28
4.9. Choice of Dose Escalation Interval 28
4.10. Study conduct 30
5. TRIAL OBJECTIVES AND PURPOSE 31
5.1. Primary objective 31
5.2. Secondary objectives 31
5.2.1. Pharmacokinetic (PK) and pharmacodynamics (PD) objectives 31
5.3. Biologic activity and biomarker objectives 31
5.3.1. Immunogenicity objective 32
6. INVESTIGATIONAL PLAN 33

6.1. Overall Study Design 33
6.2. Study Duration and Visit Schedule 34
6.2.1. Screening 34
6.2.2. Treatment and follow-up 35
6.3. Study Endpoints 35
6.3.1. Primary Endpoint 35
6.3.2. Secondary Endpoints 35
6.3.2.1. Pharmacokinetic (PK) Profile and Pharmacodynamic (PD) Endpoints 35
6.3.2.2. Biologic activity endpoints and biomarker activity 35
6.4. Number of Subjects 36
6.5. Treatment Assignment 36
6.6. Dose Adjustment Criteria 36
6.6.1. Safety Criteria for Adjustment or Stopping Doses 36
6.7. Criteria for Study Termination 38
6.7.1. Stopping Rules 39
7. SELECTION AND WITHDRAWAL OF SUBJECTS 44
7.1. Subject Inclusion Criteria 44
7.2. Subject Exclusion Criteria 44
7.3. Subject Withdrawal Criteria 46
7.3.1. Withdrawal of Consent 46
7.3.2. Failure to Return for Follow-Up 46
8. TREATMENT OF SUBJECTS 47
8.1. Description of Study Drug 47
8.2. Concomitant Medications 47
8.2.1. Required Medications 47
8.2.2. Permitted Medications 47
8.2.3. Prohibited Medications and Treatments 48
8.3. Randomization and Blinding 48
9. STUDY DRUG MATERIALS AND MANAGEMENT 49
9.1. Study Drug 49
9.2. Study Drug Packaging and Labeling 49
9.3. Study Drug Storage 49

9.4. Study Drug Preparation 50
9.5. Administration 50
9.6. Study Drug Accountability 50
9.7. Study Drug Handling and Disposal 50
10. PHARMACOKINETIC ASSESSMENTS 52
10.1. Blood Sample Collection 52
10.2. Sample Analysis 52
11. ASSESSMENT OF SAFETY 53
11.1. Safety Parameters 53
11.1.1. Demographic/Medical History 54
11.1.2. Vital Signs 54
11.1.3. Weight 54
11.1.3.1. Physical Examination/Physical Assessment 55
11.1.4. Electrocardiogram (ECG) 55
11.1.5. Laboratory Assessments 55
11.1.5.1. Hematology 55
11.1.5.2. Blood Chemistry 55
11.1.5.3. Urinalysis and Urine Protein/Creatinine Ratio 55
11.1.5.4. Coagulation 56
11.1.5.5. Pregnancy Screen 56
11.2. Adverse and serious adverse events 56
11.2.1. Definition of adverse events 56
11.2.1.1. Adverse event (AE) 56
11.2.1.2. Serious adverse event (SAE) 56
11.2.1.3. Other adverse event (OAE) 57
11.3. Relationship to study drug 57
11.4. Recording adverse events 57
11.5. Reporting adverse events 58
12. STATISTICS 59
12.1. Pharmacokinetics and pharmacodynamics 60
12.2. Clinical measures of SCD and biomarker analysis 60
12.3. Pain, analgesic use and QoL 61

12.4. Pulmonary Function 61
12.5. Immunogenicity Analysis 61
12.6. Sample Size 61
12.7. Reporting and Exclusions 61
13. DIRECT ACCESS TO SOURCE DATA/DOCUMENTS 62
13.1. Study Monitoring 62
13.2. Audits and Inspections 62
13.3. Institutional Review Board (IRB) 63
14. QUALITY CONTROL AND QUALITY ASSURANCE 64
15. ETHICS 65
15.1. Ethics Review 65
15.2. Ethical Conduct of the Study 65
15.3. Written Informed Consent 65
16. DATA HANDLING AND RECORDKEEPING 67
16.1. Inspection of Records 67
16.2. Retention of Records 67
16.3. PUBLICATION POLICY 67
17. LIST OF REFERENCES 69
18. APPENDICES 75
18.1. CDC-recommended immunizations for adults 75
18.2. Management of infusion reactions 76
19.3 World Medical Association Declaration of Helsinki 77

LIST OF TABLES

Table 1: Emergency Contact Information 3
Table 2: List of Abbreviations and Definitions of Terms 14
Table 3: Schedule of Evaluations 41
Table 4: Investigational Product 47
Table 5: Statistical Properties of the Study Design 60

LIST OF FIGURES

Figure 1: Study Schematic 40

3. LIST OF ABBREVIATIONS AND DEFINITIONS OF TERMS

Table 2: List of Abbreviations and Definitions of Terms

Abbreviation or Specialist Term	Definition
Ab	antibody
ACS	Acute chest syndrome
ADA	Anti-drug antibody
AE	Adverse event
AHR	Airway hyperreactivity
αGalCer	Alpha-galactosyl-ceramide
ALT	Alanine aminotransferase
ANA	Anti-nuclear autoantibodies
aPTT	Activated partial thromboplastin time
ASCQ-Me	Adult sickle cell quality of life measurement information system
AST	Aspartate aminotransferase
AUC	Area under the concentration-time curve
BMI	Body mass index
°C	Degrees Celsius
C_{max}	Peak concentration
C_{min}	Minimum concentration
CBC	Complete blood count
CDC	Centers for Disease Control and Prevention
CFR	(US) Code of Federal Regulations
Cl	clearance
CMV	cytomegalovirus
CPR	Cardiopulmonary resuscitation
CRF	Case report form
CRP	C-reactive protein
CRS	Cytokine release syndrome
CHX	Chest x-ray
CXR	Chest radiograph

Abbreviation or Specialist Term	Definition
CTCAE	Common terminology criteria for adverse reactions
DLT	Dose-limiting toxicity
DVT	deep venous thrombosis
EBV	Epstein-Barr virus
E. coli	*Escherichia coli*
EC	Ethics committee
ECG	electrocardiogram
eDiary	Electronic diary
ELISA	Enzyme-linked immunosorbant assay
EOI	(adverse) Event of interest
EOS	End-of-study-visit
FACS	Fluorescence-activated cell sorting
FDA	Food and Drug Administration
FEV	Forced expiratory volume in one second
FEF_{25-75}	Forced expiratory flow rate between 25% and 75% of vital capacity
FVC	Forced expiratory vital capacity
g	gram
GCP	Good Clinical Practice
Hb	Hemoglobin
HbA	Hemoglobin A
HbS/β^0	HbS and β thalassemia genes
HbSS	Homozygous hemoglobin S gene
HBV	Hepatitis B virus
HCV	Hepatitis C virus
HIV	Human immunodeficiency virus
HSV	Herpes simplex virus
HU	hydroxyurea
IB	Investigator brochure
ICF	Informed consent form
ICH	International Conference on Harmonization
IFNγ	Interferon gamma

Abbreviation or Specialist Term	Definition
IL-1β	Interleukin-1 beta
IL-6	Interleukin-6
IL-8	Interleukin-8
iNKT	Invariant natural killer T (cell)
IRB	Institutional Review Board
IRI	Ischemia-reperfusion injury
iTCR	Invariant T cell receptor
IV	intravenous
kg	Kilogram
LDH	Lactate dehydrogenase
LFT	Liver function test
LLQ	Lower limit of quantitation
mAb	Monoclonal antibody
MedDRA	Medical dictionary for regulatory activities
μg	microgram
mg	milligram
min	minute
mL	milliliter
mM	millimolar
mmHG	Millimeters of mercury
MTD	Maximum tolerated dose
NKTT	NKT Therapeutics, Inc.
NKTT120	Recombinant, humanized anti human iTCR monoclonal antibody
NOAEL	No observed adverse effect level
NS	Normal saline
NSAID	Non-steroidal anti-inflammatory drug
NY1DD	Murine sickle cell disease model
O_2	Oxygen
OAE	Other significant adverse event
PBMC	Peripheral blood mononuclear cells
PCR	Protein/creatinine ratio (urine)

Abbreviation or Specialist Term	Definition
PE	Pulmonary embolism
PEF	Peak expiratory flow
PID	Primary immunodeficiency syndrome
PFT	Pulmonary function test
PI	Principal Investigator<br>The investigator who leads the study conduct at an individual study center. Every study center has a principal investigator.
PK	Pharmacokinetic
PPD	Purified protein derivative (test for Tuberculosis)
PRBC	Packed red blood cells
PROMIS	Patient reported outcomes measurement information system
P-sel	P-selectin is a protein found on the surface of endothelial cells and platelets
PT	Prothrombin time
pVOC	Painful vaso-occlusive crisis
QoL	Quality of life
RBC	Red blood cell (erythrocyte)
RDL	Recommended dose level
SABA	Short-acting $beta_2$ agonist
SAD	Single ascending dose
SAE	Serious adverse event
Sickle cell disease	SCD
SC_r	Serum creatinine
SD	Standard deviation
SGOT	Serum glutamic oxaloacetic transaminase
SGPT	Serum glutamic pyruvic transaminase
SOE	Schedule of evaluations
$SPLA_2$	Secretory phospholipase A_2
sP-selectin	sP-selectin - A soluble plasma/serum form of P-selectin released from platelets and endothelial cells
SRC	Safety review committee
$t_{1/2}$	Half life

Abbreviation or Specialist Term	Definition
TB	Tuberculosis (Mycobacterium tuberculosis)
TIA	Transient ischemic attack
TNFα	Tumor ecrosis factor alpha
TRV	tricuspid regurgitent jet velocity
ULN	Upper limit of normal
V_d	Volume of distribution
Vol	Volume
vWF	Von Willebrand Factor
WBC	White blood cell (leukocyte)

4. INTRODUCTION

4.1. NKTT120

NKTT is developing NKTT120, a novel, humanized, IgG1κ monoclonal antibody (mAb) that specifically targets the human invariant NKT (iNKT) cell receptor (iTCR) on a subset of T cells called iNKT cells, for the treatment of sickle cell disease. NKTT120 binds exclusively and specifically to Vα24-Jα18 gene-rearranged iTCRs on human iNKT cells, resulting in depletion of iNKT cells. Preclinical data in murine sickle cell disease (SCD) models have implicated iNKT cells in the initiation of the cascade of inflammation in SCD (Field, 2011; Lappas, 2006; Wallace, 2009) and have led to the hypothesis that depletion of proinflammatory iNKT cells in blood and tissue of SCD subjects will reduce the inflammation that leads to painful vaso-occlusive crisis (pVOC), acute chest syndrome (ACS), increased airway hyperreactivity (AHR), chronic pain and tissue damage. NKTT120 will be used in this Phase 1 SAD trial to deplete iNKT cells in subjects with stable SCD.

4.2. Description of invariant NKT cells

Invariant NKT cells comprise a very small (0.01 – 1.0% of human peripheral blood T cells), but functionally important subpopulation of T cells that are activated by lipid antigens presented by CD1d (an MHC-like molecule) and that share characteristics of both innate and adaptive immune cells (Bendelac, 2007). Similar to conventional T cells, they express an alpha-beta T cell receptor (TCR) and respond in an antigen-ligand TCR-restricted manner. However, iNKT cells are activated by lipid and not peptide antigens and exhibit characteristics typical of innate immune cells, such as expression of natural killer cell markers, lack of cytokine polarization, and immediate activation and cytokine secretion upon encounter of antigens (Bendelac, 2007). In addition, iNKT cells play a critical role in the activation of other immune cell subsets through cytokine secretion and cell contact-dependent effects, and they affect the maturation of dendritic cells, activation of and cytokine secretion from NK and T cells, and antibody production by plasma cells (Galli, 2003; Chang, 2011).

Unlike other T cells, which emerge as specific clonotypic responses to a particular pathogen challenge, and which result in a unique repertoire for each individual, iNKT cells are a continuously regenerating subset of T cells. In health there is a balance between the number and activation state of iNKT cells. However, in a variety of inflammatory disease states, the balance may be skewed. iNKT cells have been implicated in the initiation and maintenance of serious inflammatory diseases, such as SCD, asthma and chronic obstructive pulmonary disease where the number and activation state of iNKT cells in the circulation and/or tissue is increased (Wallace, 2009; Reynolds, 2009; Kim, 2008).

4.3. Sickle cell disease and invariant NKT cells in vaso-occlusion in SCD

Sickle cell disease is an autosomal recessive genetic disorder of hemoglobin that affects approximately 70,000 to 100,000 people in the United States and is common in people of African and Mediterranean descent (Grant, 2011; Yusuf, 2011; Ashley-Koch, 2000; Brousseau, 2010-1). The molecular basis for SCD is a point mutation in the sixth codon of the β-globin gene. The

resulting mutant hemoglobin (hemoglobin S) polymerizes to form an intra-erythrocyte viscous gel that induces rigid, dense, sickle-shaped erythrocytes under hypoxic conditions. In the circulation, sickled red blood cells (RBCs) can occlude the vasculature, leading to tissue ischemia and ultimately result in end-organ damage. The clinical course of SCD is punctuated by episodes of acute multi-cellular vaso-occlusion that result in sudden pVOC, that are characterized by bone pain and pulmonary occlusion, which can result in ACS. Historically, the incidence of pVOC has been estimated to be between 1 to 3.5 episodes per year per subject (Platt, 1991; Brousseau, 2010-2), but more recent data demonstrate that pVOC is an ongoing pathology that occurs daily and is not restricted to acute crisis (Smith, 2008). Intermittent episodes of ischemia that occur throughout the lives of SCD subjects lead to end organ damage (Platt, 2000; Frenette, 2007), resulting in stroke, visual impairment, pulmonary hypertension and renal disease. Not surprisingly, an increased rate of pVOC is associated with premature death and the shortened life expectancy seen in men (42 years) and women (48 years) with SCD (Platt, 1994). Although the signs and symptoms of vaso-occlusion are most pronounced during pVOC, tissue ischemia and end organ damage are chronic in many subjects with SCD as evidenced by their pro-inflammatory state and reactive endothelium (Hebbel, 2004), and may be responsible for their daily pain and disability (Smith, 2008). Most subjects also experience less intense occlusive events that do not result in hospitalization, but significantly impact the activities of daily living, such as attendance at school and employment, as well as the overall quality of life of the SCD subject.

In SCD, a multi-cellular process starts with adherence of sickled RBCs to vascular endothelial cells and the subsequent vascular inflammation is characterized by increased adhesion of leukocytes and platelets (Kaul, 2000). Prior work in a non-SCD model of liver ischemia-reperfusion injury (IRI) showed that IRI and end organ damage could be ameliorated by activation of adenosine 2A receptors ($A_{2A}R$) with an adenosine analogue administered systemically during or after ischemic injury. It was subsequently found that the protective effect of activation of $A_{2A}R$ was due to blocking of iNKT cell activation (Lappas, 2006).

Notably, in a murine model of SCD (NY1DD) and in the human disease, both the absolute number of iNKT cells and the percentage of iNKT cells expressing activation markers are increased, further suggesting that the iNKT cell might very well have a role in both the development of the underlying chronic disease and the increased inflammation and injury associated with acute crisis events (Wallace, 2009; Kaul, 2000). The NY1DD mouse replicates many features of human SCD including the production of sickled RBCs and showing organ damage at baseline, which are due to the vaso-occlusion produced by hypoxia/reoxygenation injury and the cascade of inflammatory mediators that cause acute tissue ischemia and exaggerated reperfusion injury (Wallace, 2009). In the NY1DD model and other murine models of SCD, blocking iNKT cell activation using a number of different experimental methods (iNKT cell-deficient mice, anti-CD1d-blocking antibodies and $A_{2A}R$ activation) reduced tissue damage, demonstrating the central role of iNKT cells in vaso-occlusion (Wallace, 2009; Wallace, 2010).

In addition, hyper-reactive airways are common in adults and children with SCD. A study comparing 31 adults with SCD and asthmatic symptoms to 31 matched controls found that patients with SCD had significantly lower values for forced vital capacity (FVC), forced expiratory volume in 1 second (FEV_1), forced expiratory flow rate at 25% two 75% of FVC ($FEF_{25-75\%}$), peak expiratory flow rate, total lung capacity and lung diffusion capacity, testing than controls. The cause of the AHR is currently unexplained, although some studies suggest that

iNKT cells play a substantial role in the development of asthma and sustaining the disease. Furthermore iNKT cells have been shown to be increased in number and activation state in patients with SCD, and these effects are exacerbated after allergen challenge (Akbari,2006; Matangkasombut,2009; Reynolds,2009). Though these data are intriguing the precise role of iNKT cells in the AHR of SCD, as well as in asthma remains to be elucidated in clinical trials.

Treatment options for patients with SCD are limited. Other than supportive care (analgesics, blood transfusions, avoidance of cold and dehydration), hydroxyurea (HU) which increases fetal hemoglobin production, and consequently reduces RBC sickling, is the only FDA-approved drug for maintenance use in SCD. Although HU has been demonstrated to be effective in reducing pVOC events (Charache, 1995), its benefits are mitigated by poor compliance due to the requirement for daily administration and fear of complications (Candrilli, 2011; Brandow, 2010; Brawley, 2008). A new therapy such as NKTT120, which could be administered in the clinic at relatively infrequent intervals, may be an effective strategy for reducing vaso-occlusive events and end organ damage.

4.4. Nonclinical studies with NKTT120

NKTT120 recognizes human, cynomolgus monkey and rhesus monkey iTCRs, but does not recognize iTCRs from rodents and squirrel monkeys. Therefore NKTT120 cannot be used in rodent models of human diseases to explore mechanism of action or in models evaluating the safety of depleting iNKT cells. In vitro and in vivo studies have been conducted to establish the activity of NKTT120 at the molecular, cellular and tissue levels. Two nonclinical studies, a GLP pharmacokinetic and a GLP toxicokinetic study, have been conducted with NKTT120 in cynomolgus monkeys. These studies showed no adverse events related to the administration of NKTT120 and demonstrated that, following depletion of iNKT cells by NKTT120, iNKT cells return to the peripheral circulation after the NKTT120 serum concentration has reached a pharmacologically ineffective level. The key findings of these nonclinical investigations are briefly described below. More detail is available in the current NKTT120 Investigator Brochure (IB).

In vitro evaluations have shown that NKTT120 binds potently and specifically to iNKT cells in human whole blood and in PBMCs from cynomolgus monkeys. It does not bind to other lymphocytes.

In vivo, NKTT120 has been demonstrated to rapidly and specifically deplete iNKT cells in non-human primates (cynomolgus and rhesus monkeys) and in transgenic mice that express the human (Vα24Jα18) iTCR. In preliminary, non-GLP studies in cynomolgus monkeys, very low doses of NKTT120 (0.003 mg/kg) partially depleted iNKT cells, which reappeared in the peripheral circulation in approximately four days. At moderate doses (0.03 mg/kg and 0.3 mg/kg), iNKT cells were depleted within 24 hours of administration and remained depleted for the 28-day follow-up period. In longer term studies, iNKT cells were shown to return to the peripheral circulation in a dose dependent fashion approximately 5-20 weeks following single dose administration. These studies are described in more detail below.

In a GLP pharmacodynamic study, evaluating the effect of single intravenous doses of NKTT120 on iNKT cell depletion and the relationship between NKTT120 serum concentration and iNKT cell reappearance, cynomolgus monkeys were given a single IV dose of NKTT120 and monitored

for changes in iNKT cell number as well as for changes in numbers of other lymphocytes (MPI-1854-018). The doses evaluated were 0.01, 0.03, 0.1 or 0.3 mg/kg. All doses produced complete depletion of peripheral cell iNKT cells by 24 hours after dosing without causing changes in other lymphocytes. The peripheral iNKT cell numbers showed measurable numbers at 5 weeks following the 0.01 mg/kg dose, and at 8 weeks following the 0.03 mg/kg dose. The two high dose groups (0.1 and 0.3 mg/kg) began having measurable circulating iNKT cells at week 10, but their iNKT cells have not yet reached the pre-defined criterion for recovery (to ≥0.03% of CD3+ cells in all animals of a group for two consecutive weeks). No adverse events have been observed to date. The data demonstrate that after NKTT120-mediated depletion, iNKT cell reappearance can occur in a dose- and time-dependent manner.

In the repeat dose 28-day toxicity study evaluating the administration of five total doses of NKTT120 at 0.3 mg/kg, 3 mg/kg and 10 mg/kg (MPI 1854-019), no adverse events were reported at exposure concentrations greater than 100-fold the highest exposure expected to be reached in the Phase 1 trial.

The overall conclusions of these two studies are that NKTT120 is highly specific for and effective in reducing the iNKT cell population, that these cells can recover as the effective concentration of NKTT120 diminishes, and that NKTT120 is well-tolerated at exposure concentrations that are 100-fold greater than the expected highest human exposure planned in the Phase 1 study.

A human tissue cross-reactivity study was not performed, as exhaustive attempts at developing an immunohistochemical staining method with NKTT120 were not successful. As is common with some antibodies, it appears that NKTT120 cannot be optimized for tissue staining using standard immunohistochemical techniques. However, NKTT flow cytometry data show that NKTT120 binds with high specificity to iNKT cells and does not bind to other T cell subtypes or to other hematopoietic cells. The flow cytometry results are confirmed by the fact that NKTT120 rapidly deplete iNKT cells following intravenous administration in cynomolgus monkeys, but does not affect other lymphocytes or other peripheral blood cell populations.

4.5. Clinical Studies

This is the first-in-human clinical study of NKTT120. There have been no other clinical trials of NKTT120 in humans.

4.6. Potential risks of NKTT120

4.6.1. Cytokine Release Syndrome (CRS)

As with any mAb that targets lymphocytes, NKTT120 has the theoretical potential to elicit acute infusion reactions or cytokine release symptoms. First dose infusion reactions and cytokine release symptoms have been associated with a number of anti-lymphocyte mAbs (anti-CD3, anti-CD20, anti-CD52), but have been successfully managed in the clinic. Typically, this first-dose phenomenon is due to the fact that most of the targeted cells, although cleared rapidly following the first infusion, may release cytokines prior to clearance. There is a decreased target load for subsequent doses and therefore less cytokine release. Anti-lymphocyte mAbs that induce CRS typically target relatively large lymphocyte subpopulations, such as all T cells or all B cells, and thus the intensity of symptoms is directly related to the large size of the target population and the

activation of the target cell. In contrast, iNKT cells are a very small target population (approximately 0.01 to 1.0% of all T cells). Although some investigators have seen that patients with SCD have increase numbers of iNKT and activated iNKT cells in the peripheral circulation, this is still generally less than 0.1% of CD3 cells. Thus, the risk of cytokine release syndrome following administration of NKTT120 is very likely to be much lower than that associated with administration of a mAb that clears all T cells or B cells.

In addition, NKTT's GLP toxicology data in cynomolgus monkeys have shown no adverse events, including CRS, either in the hours immediately following dosing when the iNKT cells were depleted, or in the longer-term follow-up periods (up to four months). These findings are consistent with results from clinical trials testing known glycolipid activators of iNKT cells, such as α-GalCer, in which only mild-to-moderate cytokine release symptoms were noted. These clinical studies with α-GalCer are described in more detail in the IB. Whether NKTT120 can activate iNKT cells and potentially lead to CRS prior to their depletion is unknown, however *in vitro* and *in vivo* studies in humans and monkeys (discussed in the IB) with potent glycolipid activators of iNKT cells indicate that NKTT120 is highly unlikely to activate iNKT cells or other cells prior to iNKT cell depletion (Giaccone, 2002; Schneiders, 2011; Veldt, 2007; Motohashi, 2006; Motohashi, 2009; Uchida, 2008).

To further address the possibility of CRS in response to treatment with NKTT120 in humans, the effect of NKTT120 on cytokine release in vitro in human whole blood was evaluated. Incubation of whole blood in the presence or absence of 20 µg/ml of NKTT120 (a concentration higher than the predicted exposure following a dose of 1 mg/kg) for 24 hours did not result in a cytokine response in any of 4 donor samples. In contrast, incubation with an anti-CD3 mAb resulted in substantial elevation of several cytokines (IFNγ, IL-4, IL-10, IL-17, TNFα, IL-2, IL-5, IL-8, IL-1RA and MIP-1) in at least two of the four donor samples. These data suggest that in contrast to the activation of the variable T cell receptor by anti CD3, NKTT120-mediated binding to the invariant receptor of iNKT cells has no measurable cytokine-inducing effect in an *in vitro* human whole blood setting.

Although the available in vitro and in vivo data suggests that the risk of CRS following the administration of NKTT120 is low, the theoretical risk remains and subjects will be closely monitored for signs of CRS in the clinical study. Stopping the infusion and administration of a histamine blocker is usually sufficient to manage reactions for most subjects who experience mild or moderate infusion reactions. Once symptoms subside, usually within 30 minutes, the infusion can often be restarted cautiously at the investigator's discretion, at half the initial rate and titrated as tolerated, without further symptoms (Kosits, 2000).

Delayed infusion reactions are uncommon but may occur days after the infusion. Symptoms associated with delayed infusion reactions include arthralgias, skin rash, leukocytic vasculitis, urticaria, myalgias, and fever. Symptoms may be self-limiting or may improve with administration of corticosteroids (Weber, 2004; Barbaud, 2011).

4.6.1.1. Allergic Reactions and Anaphylaxis

In contrast to symptoms associated with CRS, true allergic-type reactions typically are more severe when subjects are re-challenged. In this first in human phase I trial, all subjects will be observed for acute allergic-type reactions for at least 6 hours after the infusion of NKTT120. The clinical site and clinical staff must be prepared to handle allergic reactions, including anaphylaxis

and no subject should be discharged from the clinical site if they are unstable or have any of the signs or symptoms of a delayed allergic reaction.

Delayed allergic reactions could occur, after the subject leaves the clinic; therefore, subjects will be instructed in the signs and symptoms of a delayed reaction (including swelling, tenderness, and/or itching at the site of infusion, rhinorrhea, sneezing, coughing, shortness of breath, nausea, dizziness, wheezing, rash or faintness, pruritus, urticaria, scratchy throat, throat or tongue swelling) and will be instructed to contact the clinical site, or seek medical attention, immediately if they notice any of these signs and symptoms after leaving the clinical site.

4.6.2. Depletion of iNKT cells

Chronic suppression of iNKT cells poses potential risks, as iNKT cells are thought to play a role in the response to pathogens, in immune surveillance for cancer, and in the regulation of autoimmunity (Wu, 2011; Balato, 2009). Tools such as various knockout mice that lack iNKT cells, and the synthetic glycolipid, α-GalCer, have helped to define the role of iNKT cells in health and disease, however disease model studies in mice have been hampered by the lack of a mAb that specifically recognizes the murine iTCR. In those studies performed in mice and humans, the role of iNKT cells in infection, cancer and autoimmunity have yielded conflicting results. In addition, there are significant differences between mice and humans with respect to the CD1 repertoire, the lipid antigen-presenting MHC class I-like molecules, and iNKT cell distribution and function (Bendelac, 1997), which have made extrapolation from mouse to human extremely difficult. Thus, while the real risk of depletion of iNKT cells on the response to pathogens, immune surveillance and recognition of autoimmunity is not known, and the available data as outlined below suggests the risk may be low.

4.6.2.1. Bacterial infections

Most of the information about the role of iNKT cells in infection has been generated from studies in murine models of infection and in humans with primary immunodeficiency syndromes (PIDs) that affect CD1d and iNKT cell maturation and function. Using CD1d knockout mice (which lack both iNKT cells and non-invariant NKT cells) and Jα18 knockout mice (which lack only iNKT cells, but also have a broad defect in their T cell repertoire (Bedel, 2012), it has been demonstrated that iNKT cells respond to a wide variety of microbial organisms including bacteria, viruses, parasites, and fungi (Tupin, 2007). In most of these models, iNKT cells were shown to have a protective role; however, in some cases they were shown to have a deleterious pro-inflammatory role (Nieuwenhuis, 2002; Skold, 2003; Tupin, 2007; Vincent, 2003). In other studies, CD1d knockout mice show disease exacerbation upon infection with *Pseudomonas aeruginosa*, and *Listeria monocytogenes*, whereas Jα18 knockout mice exhibit no differences in disease course from wild type mice (Arrunategui-Correa, 2004; Ranson, 2005).

There are some data in murine models suggesting that iNKT cells interact with endothelial cells through CD1d to identify pathogens and facilitate access of leukocytes to the site of infection. In murine models of *B. burgdorferi* infection absence of iNKT cells led to tissue dissemination of *B. burgdorferi* (Lee, 2010; Harding, 2012).

In a murine pulmonary TB infection model, stimulation of iNKT cells with α–GalCer resulted in increased IFNγ production and decreased intracellular TB replication and improved survival

(Sada-Ovalle, 2010). However other data suggest that deficiencies in iNKT cells could potentially predispose subjects who are infected with *Mycobacterium tuberculosis* (TB) to active disease (Montoya, 2008; Sutherland, 2009).

While there are numerous studies in mice showing that iNKT cells can assist or modulate inflammatory responses to a given infection, there are very few studies demonstrating that iNKT cell defects predispose to infectious disease. There is also very little evidence that iNKT cells are important for microbial clearance in man. It is noteworthy that cynomolgus monkeys depleted of iNKT cells for more than four months in our GLP toxicology studies did not exhibit any signs of infection. They also exhibited a normal T cell antibody response to KLH challenge and re-challenge by mounting robust IgM and IgG responses, despite complete depletion of peripheral iNKT cells. In summary, both iNKT cells and non-invariant NKT cells can contribute to pathogen defense and it has been shown that, at least for some infections, the presence of non-invariant NKT cells would seem to suffice for immune defense against pathogens. It should be reiterated that NKTT120 does not reduce the non-invariant NKT cell population.

4.6.2.2. Increased susceptibility to viral infections

There are a number of studies in the literature that describe increased susceptibility to Herpesviridae family viruses [herpes simplex viruses (HSV1, HSV2), varicella zoster virus (VZV), cytomegalovirus (CMV), and Epstein-Barr virus (EBV)] in mice and humans with deficient iNKT cell numbers or function. CD1d knockout mice (lack iNKT cells and non-invariant NKT cells) exhibit increased susceptibility to HSV1, HSV2 and murine CMV infections (Broxmeyer ,2007; Grubor-Bauk, 2003; Ashkar, 2003). One study demonstrated an association between low peripheral iNKT cell numbers and reactivation of VZV infection (Novakova, 2011).

A number of human PIDs that affect CD1d expression and function, thymic selection and maturation of T cells (including iNKT cells), and iNKT cell function have been described, and some are associated with decreased peripheral iNKT cell numbers and function, and demonstrate increased susceptibility to EBV infection or EBV-associated lymphoproliferation and lymphoma (Astrakhan, 2009; Tupin, 2007). However, other closely-related immunodeficiencies exhibit similar phenotypic characteristics, including susceptibility to EBV infection and lymphoproliferation, but show unaltered numbers of peripheral iNKT cells (Albayrak, 2009). Importantly, individuals with abetalipoproteinemia, who exhibit selective defects in CD1 and NKT cells, did not exhibit increased susceptibility to EBV (Zeissig, 2012). These results suggest that lymphoproliferation and susceptibility to EBV might not be the sole consequence of iNKT cell dysfunction or deficiency, but rather of other cellular defects, either alone or in combination with iNKT cell deficiency. Many primary genetic NK cell defects associated with susceptibility to Herpesviridae have been described and often show unaltered NKT cell function (Orange, 2002). Furthermore, genes associated with lymphoproliferative diseases are expressed by NK cells and are critical for NK cell function, thus it is likely that NK cell defects contribute to these disorders. Similarly, in other PIDs associated with iNKT cell deficiency, immunodeficiency is likely the consequence of combined immunologic defects and not the direct result of alterations in iNKT cell function.

There is no known human condition with a specific defect in iNKT cells. Individuals with combined defects in NKT cells and NK cells have been described. They appear to have an increased susceptibility to infection with Herpesviridae family members (Orange, 2002). There

are two important things to note. First, these individuals who lack NKT cells do not appear to have increased susceptibility to other kinds of pathogens. Second, an increased number of infections with Herpesviridae family members are seen in individuals with isolated defects in only NK cells. It is difficult to draw firm conclusions from these reports as all of these conditions are quite rare.

4.6.2.3. Cancer

iNKT cell function is decreased in humans with cancer (Tahir, 2001; van der Vliet, 2008), however, it is not clear whether the changes in iNKT cell function are a cause or result of the disease. In a study of subjects with multiple myeloma, the iNKT cell defect worsened with progression from pre-malignant to malignant disease, suggesting that iNKT cell defects are a result, rather than a cause, of cancer (Fuji, 2003). Mice deficient in iNKT cells do not exhibit increased incidence of spontaneous cancer, but they do show increased susceptibility to numerous experimental cancers (Berzofsky, 2009). Increased incidence of cancer in two strains of mice with a genetic absence of iNKT cells has also been observed (Swann, 2009). Conversely, in murine models of cancer, there is a clear therapeutic benefit of iNKT cell activation by αGalCer (Chang, 2007) or adoptive transfer of iNKT cells. There is limited evidence to suggest that iNKT cell deficiency increases the risk of cancer in mice, absent a strong genetic predisposition (Renukaradhya, 2008). Finally, there are no data regarding the role of transient iNKT cell depletion and the risk of cancer.

4.6.2.4. Enhanced autoimmunity

The role of iNKT cells in regulating autoimmunity is controversial, as evidence supporting both a protective and adverse role for these cells in preclinical models of autoimmune disease can be found (Wu, 2009). Jα18 knockout mice (that lack iNKT cells and some of the T cell repertoire) do not develop overt autoimmune disease, but they do develop anti-DNA antibodies (Sireci, 2007). In many human autoimmune diseases, peripheral iNKT cell numbers are decreased, and in some cases functional defects have been identified (van der Vliet, 2001). As is the case with cancer, what is not well-understood is whether these abnormalities are a cause of the disease or a consequence of the disease or its treatment.

4.6.2.5. Fertility and embryogenesis

The role of iNKT cells in embryogenesis, reproduction, and spermatogenesis is not well understood. Mice that lack iNKT cells have normal fertility and pregnancies.

Reproductive toxicology studies have not yet been performed with NKTT120. Subjects of child-bearing potential are required to use appropriate birth control methods. Pregnant and breast-feeding women should not receive NKTT120. Women of child-bearing potential desiring to become pregnant should not receive NKTT120 or should wait for the elimination of NKTT120 from the circulation and the recovery of peripheral iNKT cells before becoming pregnant. The effect of NKTT120 on normal spermatogenesis is unknown.

4.6.3. Potential risk specific to adults with SCD and risk mitigation

The potential risks associated with administration of NKTT120 may be enhanced by the pathophysiology of SCD. Subjects with clinical or subclinical organ damage due to SCD may

have a worse outcome following an AE than healthy subjects without SCD. Subjects will undergo routine pre-screening for evidence of end organ damage and subjects with clinical evidence of significant end organ damage will be excluded from participation in the trial.

Patients with SCD are functionally asplenic and are at risk for infection with encapsulated bacterial organisms. Subjects with ongoing infections will be excluded from the study. All subjects must have received CDC-recommended immunizations for adults, including immunizations for streptococcus pneumonia (Pneumovax) and *N. meningitides* [Meningococcal conjugate vaccine, quadrivalent (MCV4)] unless contra-indicated (see Section 18.1). While prophylactic antibiotics are generally not used in adults with SCD, subjects currently taking prophylactic antibiotics will be allowed to participate without discontinuing prophylactic antibiotics. Subjects not currently taking prophylactic antibiotics will not be required to start prophylactic antibiotics. Subjects will be monitored throughout the study for infections.

Due to the potential risk of activation of latent TB infections, subjects will be screened for recent exposure to TB and for evidence of active or latent TB by QuantiFERON-Gold® test and chest X-ray (CXR). Subjects with a history of infection with atypical mycobacterium will be excluded from the study.

Due to the potential risk of viral infection exacerbations during the period when iNKT cells are depleted, subjects will be screened for recent exposure to childhood viral diseases, including varicella, or exposure to influenza prior to enrollment and must be current on all CDC-recommended viral immunizations for adults. The CDC recommendations for adult immunizations are provided in section 18.1. Subjects will be monitored clinically for viral infections (especially Herpesvirus family viruses) throughout the study.

Subjects with a history of cancer will be excluded from the study and subjects in the study will be clinically monitored for the emergence of cancers during follow-up, until one month after recovery of iNKT cells.

Subjects with a known autoimmune disease (including type 1 diabetes) will be excluded from the study. All subjects will be monitored for treatment-emergent signs of autoimmune disease. Serum for evaluation of autoantibodies will be stored for future analysis of autoantibody status. These samples will be used to confirm a diagnosis of a new autoimmune disease or drug-induced lupus.

4.7. Subject population and study rationale

The planned Phase 1 study will be performed in adults with “stable” SCD, defined as not having experienced acute pVOC, ACS or other major or significant SCD-associated event requiring hospitalization or outpatient medical care during the month prior to enrollment. The stable SCD population was specifically chosen as appropriate for the first-in-human study because the primary goal of the Phase 1 study is to evaluate the safety profile of NKTT120 and to determine the MTD or the recommended dose for future clinical trials, in a relevant population. Healthy volunteers who are frequently enrolled in Phase 1 studies, were not selected for evaluation of NKTT120 because they do not show increased numbers of iNKT cells or activated iNKT cells in the peripheral circulation. Therefore, determination of the MTD and safety profile in healthy volunteers might not be relevant to the SCD population. NKTT120, by specifically depleting only iNKT cells, has the potential to decrease the inflammatory component associated with IRI and to

decrease acute pVOC and ACS events. In the longer-term, chronic pain, AHR, and end organ damage may be improved, thus leading to an improved QoL for SCD patients.

4.8. Dose Rationale

In selecting a starting dose and planned range of doses to be studied, consideration was given to the maximum recommended safe dose (MRSD) calculated using standard approaches (FDA Guidance for Estimating the Maximum Safe Starting Dose in Initial Clinical Trials for Therapeutics in Adult Healthy Volunteers), evaluation of safety factors, and an anticipated pharmacologically active dose (PAD).

The human equivalent dose (HED) to the No Observed Adverse Effect Level (NOAEL) determined in the GLP repeat dose toxicology study in cynomolgus monkeys was 3.2 mg/kg. Using a 10-fold safety factor, the MRSD is 0.32 mg/kg. The HED of an expected PAD is 0.001 mg/kg. Therefore, the planned starting dose of 0.001 mg/kg is 1/300th of the MRSD and 1/3,000th of the NOAEL.

The highest planned study dose of 0.1 mg/kg is 1/3rd of the MRSD. Therefore, the planned dose range provides for an expected margin of safety at the lowest and highest doses. However, as described in Sections 6.1 and 6.6 of this protocol, the planned doses may be revised based on the evolving understanding of both safety and pharmacologic activity obtained throughout the study.

4.9. Choice of Dose Escalation Interval

This study incorporates a two-week interval to ensure subject safety in an existing cohort before escalating the dose in a new cohort. This time interval was chosen as a result of:

- Preclinical in vitro studies, and in vivo studies in non-human primates that show the lack of cytokine release phenomenon or adverse impact of iNKT cell depletion over a wide range of doses, repeat dosing and follow-up periods, and
- The time period when iNKT cell-related DLT events might occur, as a direct result of either the NKTT120 antibody infusion, or the consequently rapid reduction in iNKT numbers (<24 hours) and its consequent effect on SCD. Safety issues related to prolonged iNKT depletion as a result of high doses of NKTT120 are also a consideration, and are discussed in detail below. The safety monitoring plan provides for subject safety in this phase 1 SAD trial in that the SRC will be immediately aware of all SAEs and reviewing all AEs on a routine basis as subjects are followed through 30 days after iNKT cell recovery.

A wide dose range of NKTT120 has been administered to cynomolgus monkeys as the most relevant responsive species, and no obvious acute or chronic adverse clinical responses to NKTT120 treatment have been observed. Importantly, there have been no acute or chronic elevations of cytokines that would be associated with systemic iNKT cell activation with observations of 1 day to >20 weeks post dosing, conservatively supporting the safety of a 2 week dosing interval between cohorts.

To provide highest levels of subject safety, the SRC will be intensely evaluating subjects on an ongoing basis post dosing for the two types of potential toxicities:

- NKT-specific dose limiting toxicities such as cytopenias, infections, and cytokine storm syndrome
- Sickle cell-specific dose limiting toxicities such as hemolysis, acute vaso-occlusive episodes, any provider encounter (clinic, ED or hospital) for increased pain in the extremities, chest, abdomen or head lasting for at least 2 hours, requiring opioid administration that cannot be explained except by sickle cell disease.

This trial initiates dosing at 0.001 mg/kg, a dose 1/300th the maximum recommended starting dose (MRSD) based on the 10 mg/kg no observed adverse effect level (NOAEL) determined from the repeat dose GLP toxicology study, and 1/3000th the human equivalent NOAEL dose. The highest dose we propose to administer in this study, 0.1 mg/kg, is 1/3 of the MRSD (0.32 mg/kg) estimated from the NOAEL determined in the repeat dose toxicology study. The lack of significant toxicity within the 1-3 months of high exposure of NKTT120 achieved in this study supports our proposed dosing interval.

The primate data derived from the 28 day toxicology study and the single dose depletion recovery study show that NKTT120 ADCC-mediated iNKT cell depletion is maximal within one day, and suggest that the duration of the effect is related to the plasma concentration necessary to eradicate regenerating iNKT cells as well as to the extent of iNKT cell pool depletion. At the lower levels, the doses proposed for this trial should only provide peripheral depletion of iNKT cells, and are not expected to result in complete tissue depletion. The safety profile in monkeys, together with the fact that the iNKT cell is the only target for NKTT120 argue that despite the estimated human half-life of NKTT120 of ~22 days (based on the 11 days half-life observed in monkeys), one would not expect to see additional adverse effects from the decreasing levels of the antibody alone. The data from the single dose depletion recovery study suggests that iNKT cells recovery will occur in all subjects.

Through their effects on other immune cell subsets (dendritic cells, NK, T cells and B cells), iNKT cells have been implicated in a wide variety of physiological and pathophysiological processes {Matsuda, 2003; Nakai, 2004; Fujii, 2003; Fujii, 2007; Yoshimoto, 2003; Galli, 2003; Chang, 2012; King, 2012; Wu, 2009; Berzofsky, 2009; Tupin, 2007). Thus, chronic suppression of iNKT cells poses potential risks, as iNKT cells are thought to play a role in the response to pathogens, in immune surveillance for cancer, and in the regulation of autoimmunity (Wu, 2011; Balato, 2009). However, studies in mice and humans evaluating the role of iNKT cells in infection, cancer and autoimmunity have yielded conflicting results. Tools such as various knockout mice that lack iNKT cells, and the synthetic glycolipid, α-GalCer, have helped to define the role of iNKT cells in health and disease; however, disease model studies in mice have been hampered by the lack of a mAb that specifically recognizes the murine iTCR. In addition, there are significant differences between mice and humans with respect to the CD1 repertoire, the lipid antigen-presenting MHC class I-like molecules, and iNKT cell distribution and function (Bendelac, 1997), which have made extrapolation from mouse to human extremely difficult. While infection is a theoretical risk of prolonged iNKT cell depletion, and probably of the greatest concern in this patient population, there is little evidence to suggest that a lack of iNKT cells increases the risk of predisposition to infection in preclinical models (Berzins, 2011).

Even if prolonged iNKT cell depletion were to result in an increase in the risk of infection, it would likely need to be a markedly increased risk in order for it to be detected in a small, single

dose study in an at-risk population. If there is a dramatic increase in susceptibility to infection, it will likely emerge in the first two weeks of iNKT cell depletion, and further increasing the dosing interval between cohorts will neither have a material impact on our ability to detect such a DLT, nor meaningfully reduce the clinical risk to subjects in the proposed study.

4.10. Study conduct

The following characteristics of an adequate and well-conducted trial will be implemented:

1. The investigators will be well-qualified by scientific training and experience
2. Detailed electronic Case Report Forms (eCRFs) will be completed for every subject
3. Requirements for institutional review as set forth in ICH E6 and Good Clinical Practice (GCP) guidelines, and Title 21 Code of Federal Regulations (CFR), Part 56 will be followed
4. Requirements for informed consent set forth in ICH E6 (GCP) and 21 CFR, Part 50 will be followed
5. Safety data will be recorded and evaluated
6. Routine monitoring visits will be conducted by the Sponsor's representative to ensure data accuracy

This trial will be conducted according to the US Food and Drug Administration 21 CFR, Part 50, ICH Harmonized Tripartite Guidelines for Good Clinical Practice (Guidance E6), the Declaration of Helsinki (18.3) and all local laws and regulations concerning clinical studies and the protection of study subjects under the sponsorship of NKT Therapeutics Inc.

5. TRIAL OBJECTIVES AND PURPOSE

5.1. Primary objective

The primary objective is to assess the safety and MTD, or RDL for Phase 2, of a single ascending dose of IV NKTT120 in adult subjects with stable SCD. If the highest dose level is reached without determining a MTD, the RDL(s) for phase 2 will be determined by a dose that is at or below the highest dose and which is associated with iNKT cell recovery that allows dosing every 4-6 months in phase 2 studies.

5.2. Secondary objectives

1. To assess the PK profile of IV NKTT120
2. To assess the PD profile of peripheral iNKT cell depletion and recovery following IV NKTT120, including the recovery of the population of activated iNKT cells as measured by the expression of CD 69.
3. To evaluate change in clinical measures of SCD activity (e.g., leukocyte counts, Hgb, reticulocytes, platelets, LDH, hsCRP)
4. To evaluate the effect of NKTT120 on daily pain and use of analgesic medication compared to screening values (e.g., 2-week screening run-in daily pain score and analgesic use)
5. To evaluate the effect of the administration of NKTT120 on AHR comparing PFTs (including FVC, FEV_1, $FEF_{25\text{-}75\%}$) and PEF to screening values
6. To evaluate changes in QoL and pain compared to screening values (ASCQ-Me, PROMIS short-form and eSCaPe e-Diary).
7. To explore a number of serum and cellular biomarkers of inflammation, coagulation and endothelial function (e.g. IFNγ, TNFα, IL1β, IL-6, IL-8, sPLA2, sP-selectin, F1+2, D-dimers and vWF) compared to screening values
8. To evaluate anti-drug antibodies (ADAs) following single dosing

5.2.1. Pharmacokinetic (PK) and pharmacodynamics (PD) objectives

The PK objective is to determine the PK profile of NKTT120 and the relationship of PK to depletion and recovery of iNKT cells, adverse event profile and presence of ADAs.

The PD objective is to determine the profile of iNKT cell depletion and recovery post-dosing with NKTT120 and to correlate the PK and PD profiles. It is anticipated that iNKT cells will recover after NKTT120 is eliminated from or at present at very low concentrations in the serum.

5.3. Biologic activity and biomarker objectives

To investigate the biologic activity of NKTT120, a number of clinical assessments will be performed. These assessments will evaluated pre- and post-dosing at appropriate intervals and will be correlated with the PK/PD profile of NKTT120. They include:

1. Clinical measures of SCD activity (e.g., leukocyte counts, hemoglobin, reticulocytes, platelets, LDH, CRP)
2. Pain frequency and intensity and use of analgesics as recorded daily in an eDiary
3. AHR and use of SABA, both recorded daily in an eDiary, and additional assessment of AHR determined by PFTs at clinic visits
4. QoL as determined by PROMIS short form and ASCQ-Me questionnaires administered in the clinic

To explore the biologic effects of depletion of iNKT cells on biomarkers of inflammation, coagulation, endothelial activation, and a number of serum and cellular biomarkers will be assessed (e.g., IFNγ, TNFα, IL-1β, IL-6, IL-8, F1+2, D-dimers, vWF, sPLA2 and sP-selectin) pre- and post-dosing at clinic visits. Results will be correlated with the PK/PD profile of NKTT120.

5.3.1. Immunogenicity objective

To determine the immunogenicity of NKTT120, the presence or absence of ADAs will be determined in blood samples taken pre-dosing and at the EOS visit.

6. INVESTIGATIONAL PLAN

6.1. Overall Study Design

This is an open-label, multi-center, SAD Phase 1 study aimed at determining the MTD or RDL for Phase 2 of intravenously administered NKTT120 in adult subjects with stable SCD. For the purpose of this protocol, stable SCD is defined as not having experienced acute pVOC or ACS or other SCD-associated event requiring hospitalization or outpatient care within one month prior to dosing. In addition to determining the safety profile of NKTT120 in subjects with stable SCD, this study will also examine pain, analgesic use, QoL, pulmonary function, and inhaled SABA use. During the two-week screening run-in period and throughout the follow-up, subjects will keep a daily eDiary for pain, analgesic use, and inhaled SABA use. The ASCQ-Me and PROMIS QoL questionnaires will be administered at clinic visits. The screening run-in outcomes will be used as baseline comparison for values obtained post-dosing.

The study will evaluate single doses that are escalated over a range from 0.001 mg/kg to 0.1 mg/kg (0.001, 0.003, 0.01, 0.03, and 0.10 mg/kg). As the study progresses, the current dose escalation plan of approximately half log increment may be reduced to a slower arithmetic increment of dose escalation without increasing the number of maximum subjects if information about iNKT cell recovery times from the lower dose cohorts suggests it is necessary. Similarly, a dose de-escalation using 3+3 enrollment will be applied if indicated by the data and recommended by the Clinical Investigators and/or SRC.

Only one subject will be dosed per day and each subject will remain in the clinic for at least 6 hours after dosing for evaluation of any acute toxicity (i.e., infusion reactions, CRS, or allergic reactions) and for collection of post-dosing blood samples. Should CRS of any type be observed in subjects in any cohort, premedication with acetaminophen (1000 mg), histamine 2 (H2) receptor antagonists (diphenhydramine, 50 mg) and corticosteroids may be initiated prior to dosing of subsequent subjects to lessen the severity of CRS.

Dose escalation will be governed by a 3+3 design as follows:

- Dosing will begin at the lowest dose (minimally effective dose level), with 3 subjects per cohort.
- Each subject in the cohort must have completed at least two weeks of follow-up post-dosing before the first subject in the next cohort is dosed.
- If 0 of 3 subjects in the cohort experiences a DLT, escalation to the next dose level cohort may proceed.
- If 1 of 3 subjects in the cohort experiences a DLT, 3 additional subjects will be added to the cohort.
- If ≥ 2 of 3 - 6 subjects experience a DLT, the MTD has been exceeded and dose escalation will cease. Three (3) subjects will be added to the previous lower dose cohort(s). The MTD is defined as the highest dose at which no more than one of six subjects experiences a DLT in the dose cohort.

A SRC will review all safety and laboratory data on all subjects in the study at regular intervals. The SRC will also be made aware of any adverse EOIs that could be potential DLTs and any SAEs as soon as they are reported. Because of the complex nature of the disease, before any EOI is determined to meet DLT criterion, the SRC will meet, ad hoc, to review the safety data for an individual before declaring the event is a DLT or not. While the SRC is determining if a DLT has occurred, no additional subjects will be enrolled.

The SRC will also assess safety signals that may become evident in the longer-term follow-up and will make recommendations to the Sponsor regarding study conduct and risk mitigation, as well as dose de-escalation protocol changes.

Note that peripheral iNKT cells will be followed as a pharmacodynamic marker of drug activity and that depletion of iNKT cells is an expected outcome and will not be considered a DLT. Acute pVOC and ACS or other SCD-associated AEs (e.g., hemolysis, hepatic/splenic sequestration, decreased haptoglobin, hemoglobinuria, bone pain, chest wall pain, pain in the extremity, priapism in men, gall stones, leg/ankle ulcers, avascular necrosis of bone, stroke, cognitive disabilities) will not automatically be considered DLT, but will be evaluated on a case-by-case basis and may be elevated to DLT status if warranted and related to NKTT120. This decision will be made by the SRC.

The primary endpoint (MTD or RDL) will be completed when the last subject dosed completes dosing plus two weeks of follow-up (time at which next cohort would have started). However, each subject will be followed until 1 month after their iNKT cells have returned to detectable levels. If the highest dose level is reached without determining a MTD, the RDL(s) for phase 2 will be determined by a dose that is at or below the highest dose and which is associated with iNKT cell recovery that allows dosing every 4-6 months in phase 2 studies.

6.2. Study Duration and Visit Schedule

Enrollment through follow-up is estimated to require 18 months. The study continues until MTD has been determined or the upper dose limit of this protocol has been reached without determining the MTD. The dose-level cohort that represents the RDL will be expanded to 6 subjects to confirm the RDL.

All study assessments by visit are defined in the Table 3 and safety assessments in Section 11 .

6.2.1. Screening

A two-tiered screening will be conducted in the one-month period prior to dosing. The first tier is designated the pre-run-in screening (Table 3) and will take place one month prior to dosing. At this screening, the subject will be guided through the informed consent process and a preliminary evaluation of suitability for the study, including a medical history and a blood draw for assessment of peripheral iNKT cell numbers. It is important to note that if a subject does not have measurable peripheral iNKT cell numbers, he/she cannot participate in the trial.

If a subject meets the criteria defined in the first tier of screening, the subject will take part in the second tier of screening two-weeks prior to dosing (Table 3). If the subject meets the criteria defined in the second tier of screening, the subject will be instructed in the use of the eDiary and scheduled for dosing in two weeks.

Note that due to the schedule of events, subjects may only be dosed on Mondays or Tuesdays so that all subsequent, in-clinic evaluations may occur Monday through Friday.

6.2.2. Treatment and follow-up

Treatment consists of a single IV dose of NKTT120 administered slowly over ten (10) minutes.

Each subject will be followed until one month after iNKT cells have recovered to detectable levels. Total duration of follow-up post-dosing will vary based on the dose level received. Currently it is anticipated that iNKT cells will return to detectable levels within 1 month after NKTT120 is no longer detectable in the serum; in the lower dose cohorts; at the highest dose (0.1 mg/kg) this could be ≥ 6 months based on preliminary PK in non-human primate).

6.3. Study Endpoints

6.3.1. Primary Endpoint

The primary endpoint is the MTD or RDL. The MTD is determined by a cohort in which 2 of 6 subjects experience a DLT. The study continues until MTD has been determined, or until the upper dose limit of this protocol has been reached without reaching the MTD. The dose-level cohort that represents the MTD will be expanded to 6 subjects to confirm MTD.

If the highest dose level is reached without determining a MTD, the RDL(s) for phase 2 will be determined by a dose that is at or below the highest dose and which is associated with iNKT cell recovery that allows dosing every 4-6 months in phase 2 studies.

6.3.2. Secondary Endpoints

6.3.2.1. Pharmacokinetic (PK) Profile and Pharmacodynamic (PD) Endpoints

Calculated PK parameters for NKTT120 will be determined, including C_{max}, C_{avg}, AUC, $t_{½}$. and V_d.

The PD endpoint consists of iNKT cell depletion and recovery after dosing with NKTT120. Recovery is defined as the detectable presence of iNKT cells.

6.3.2.2. Biologic activity endpoints and biomarker activity

The following endpoints will be used to determine the biologic activity of NKTT120:

- Changes in clinical measures of SCD activity, including changes from baseline in leukocyte counts, hemoglobin, reticulocytes (number and percentage of RBCs), platelet count, LDH, and CRP
- Change in pain frequency and intensity and use of analgesics compared to screening run-in from daily eDiary
- Changes in pulmonary function assessed at clinic visits as follows: forced vital capacity (FVC), forced expiratory volume in one second, forced expiratory flow rate at 25% to 75% of FVC ($FEF_{25–75}$), peak expiratory flow rate, total lung capacity compared to screening run-in values

- Changes in QoL domains defined by PROMIS short-form and ASCQ-Me domains, compared to screening run-in

The effect of iNKT cell depletion on biomarkers of inflammation, coagulation and endothelial activation will be explored by assessing changes from baseline in serum IFNγ, TNFα, IL-1β, IL-6, IL-8, F1+2, D-dimers, vWF, sPLA2 and sP-selectin.

6.3.2.2.1. Immunogenicity endpoint

Immunogenicity of NKTT120 will be assessed by the presence or absence of anti-NKTT120 antibodies at EOS compared to baseline.

6.4. Number of Subjects

Between 2 and 30 subjects are possible for enrollment under the 3+3 design.

6.5. Treatment Assignment

There is no randomization in this trial. Eligible subjects will be enrolled into a phase I dose cohort determined by the time of their enrollment and the cohort that is still open for enrollment.

6.6. Dose Adjustment Criteria

The lowest dose is based on the lowest dose of NKTT120 in the non-GLP cynomolgus monkey studies that resulted in 50% depletion of peripheral iNKT cells within 24 hours of dosing and their return to 80% of baseline by day four post-dosing. This dose represents the lowest pharmacologically active dose in cynomolgus monkeys. The highest proposed dose level (0.1 mg/kg,) is a dose level that might deplete iNKT cells in tissue for up to four months or longer; administering this dose to subjects would be contingent on the predicted exposure being less than that associated with the no adverse effect level (NOAEL) dose determined in the nonclinical GLP toxicology studies.

As the study progresses, the current dose escalation plan of approximately half log increment may be reduced to a slower arithmetic increment of dose escalation without increasing the number of maximum subjects if required due to information based on iNKT recovery times from the lower dose cohorts. Similarly, a dose de-escalation using 3+3 enrollment will be applied if indicated by the data and recommended by the Clinical Investigators and/or SRC.

6.6.1. Safety Criteria for Adjustment or Stopping Doses

DLT will be evaluated for each subject during the two-week interval following dosing. This interval will allow identification of any acute, dose-limiting events that will determine dose-escalation or expansion of dose-level cohorts. All EOI will be reviewed by the SRC to determine if they are a DLT.

Subjects who experience a DLT will remain in the study to complete the safety evaluations and follow-up until resolution or stabilization of the DLT. All AEs and SAEs will be determined and graded for intensity based on CTCAE criteria. AEs and SAEs will be determined to be

dose-limiting if they occur during the first two weeks post-dosing and meet the criteria described below.

All safety data, including adverse events and laboratory abnormalities, will be reviewed by the SRC periodically in order to assist the sponsor in evaluating potential safety signals during the follow-up until iNKT cells recover.

Definition of DLT: a DLT is defined only for adverse events that occur within 2 weeks of dosing (with the exception of any acute vaso-occlusive episode).

Any adverse event grade 3 or higher on NCI CTCAE version 4.03 will be considered an EOI and a potential dose limiting toxicity.

All adverse EOI below that could be dose-limiting toxicities will be reviewed immediately by the SRC to determine if they constitute a DLT. All serious adverse events at any time will be reviewed by the SRC so that the SRC can make recommendations to the Sponsor regarding dosing cohorts.

Definition of EOI: an EOI is defined only for adverse events that occur within 2 weeks of dosing (with the exception of any acute vaso-occlusive episode). Any adverse event grade 3 or higher on NCI CTCAE version 4.03 will be considered an EOI.

Additionally, the following study and disease-specific toxicities will be considered an EOI and evaluated to determine if a DLT has been experienced:

iNKT cell-specific dose limiting toxicities:

- Cytopenias. Defined as CTCAE version 4.0 grade 3 or 4 leukopenia, neutropenia, lymphopenia or thrombocytopenia. Specific grade 3 definitions in CTCAE version 4.03 are:
  - Leukopenia: <2000 cells/cubic milliliter
  - Neutropenia: <1000 cells/cubic milliliter
  - Lymphopenia: <500 cells/cubic milliliter
  - Thrombocytopenia: < 50/microliter
- Infections. Defined as CTCAE version 4.03 grade 3 or 4. Specific infections are detailed in the CTCAE. Broadly the definition is a severe infection that requires IV antibiotic, antifungal or antiviral therapy, or otherwise severe and medically significant, but not necessarily life-threatening.
- Cytokine storm syndrome. Defined as CTCAE version 4.03 grade 3 or 4. The grade 3 definition reads: Prolonged (eg, not rapidly responsive to symptomatic medication and/or brief interruption of infusion); recurrence of symptoms following initial improvement; hospitalization indicated for clinical sequelae (eg, renal impairment or pulmonary infiltrates)

Sickle cell-specific dose limiting toxicities:

- Hemolysis:
  - Anemia: >25% decrease in hemoglobin from baseline or ≤ 5.0 g/dL; or

  - Lactate dehydrogenase increase 3-fold from baseline; or
  - Reticulocyte count increase 3-fold from baseline; or
  - AST increase 3-fold from baseline; or
  - Total bilirubin increase 3-fold from baseline
- Acute vaso-occlusive episode: within the 6 hour the monitoring window following infusion, an increase in pain over baseline (≥ 4 points on a 10 point pain scale) in the extremities, chest, abdomen or head lasting for at least 2 hours that could not be explained except by sickle cell disease; or
- Within 72 hours following infusion, a provider encounter (clinic, ED or hospital) for increased pain in the extremities, chest, abdomen or head lasting for at least 2 hours, requiring opioid administration and could not be explained except by sickle cell disease.

Definition of events not considered as a DLT:

The following AEs of any grade will not be considered a DLT:

- Chelitis
- Dry Skin
- Nail changes
- Hot flashes/flushes
- SCD associated pain

Decreases in the numbers of iNKT cells is an expected pharmacodynamic response to NKTT120 and will not be considered a DLT.

Acute pVOC and ACS or other SCD-associated AEs (e.g., hemolysis, hepatic/splenic sequestration, decreased haptoglobin, hemoglobinuria, bone pain, chest wall pain, pain in the extremity, priapism in men, gall stones, leg/ankle ulcers, avascular necrosis of bone, stroke, cognitive disabilities) are not unexpected disease-specific events and will not automatically be considered DLT, but will be evaluated on a case-by-case basis and may be elevated to DLT status if warranted and determined to be related to NKTT120.

Episodes of pVOC defined as “crisis” and self-treated by the subject will be recorded in the eDiary and will be recorded as an AE on the CRF, but will not be defined as DLT.

6.7. Criteria for Study Termination

NKTT and the Investigator reserve the right to terminate this study at any time. In terminating the study, NKTT and Investigator will assure that adequate consideration is given to the protection of the subjects’ safety and interests. This is an open-label study and review of subject safety outcomes will be made on an ongoing basis.

6.7.1. Stopping Rules

Enrollment in the study will be stopped and administration of NKTT120 will be halted for all newly enrolled subjects if the SRC detects a potential life-threatening safety signal during the study, including during the long-term follow-up of the subjects, and notifies the sponsor that they recommend stopping of dosing of any additional subjects. Adverse events will be evaluated to determine if they are unanticipated effects of the treatment. These events include, but are not limited to:

1. Serious, medically important or opportunistic infections requiring treatment with parenteral antibiotics (and/or empirical use of antibiotics when subject is admitted to hospital or emergency department for pVOC or other event)
2. Cancers of type or rate unexpected in the adult SCD population.

This is a single dose study, and once enrolled and dosed, subjects cannot be considered removed from study, and all attempts must be made to follow the subject for safety reasons.

Figure 1: Study Schematic

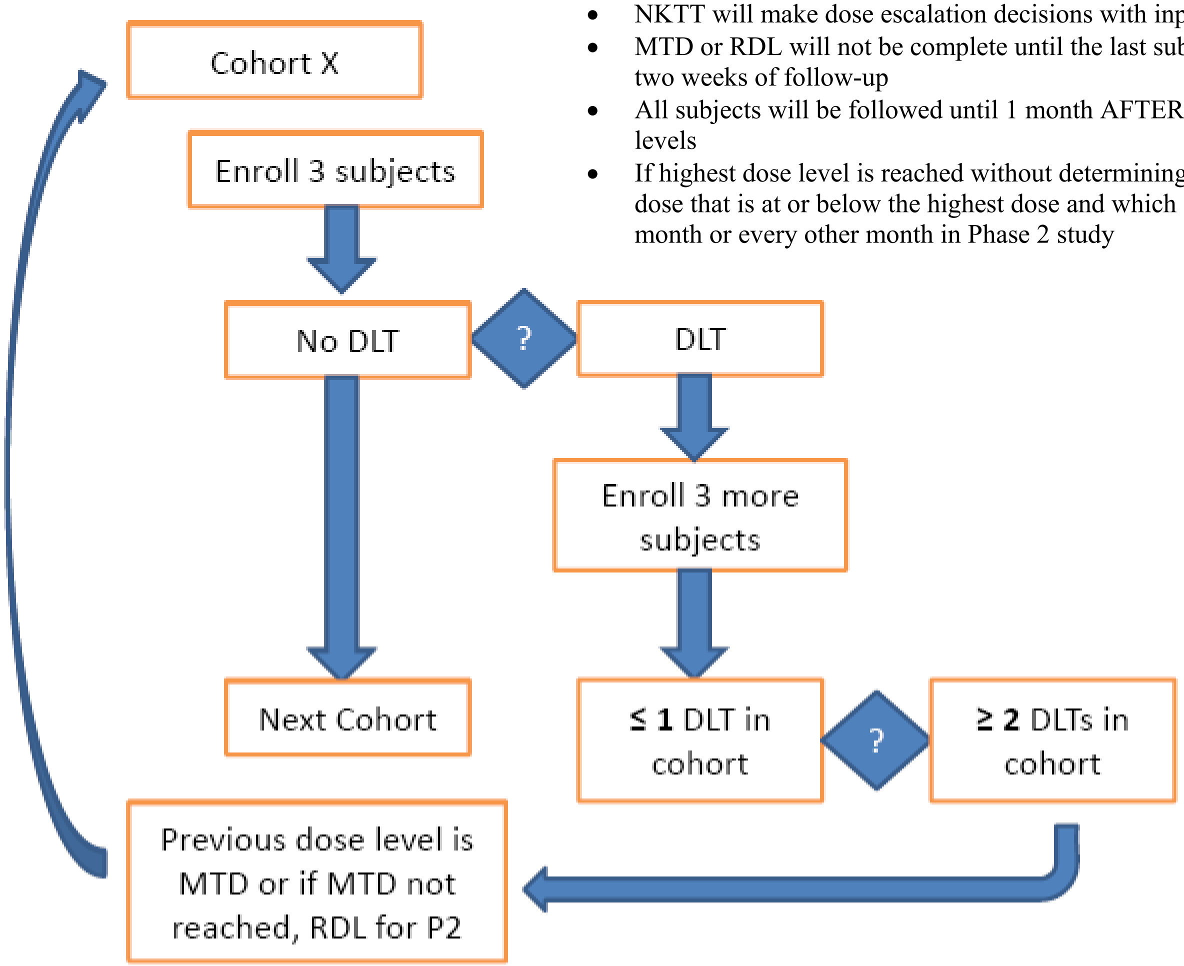

Study Design Notes

- 2-30 subjects
- NKTT will make dose escalation decisions with input from Scientific Review Committee
- MTD or RDL will not be complete until the last subject dosed in a cohort completes dosing plus two weeks of follow-up
- All subjects will be followed until 1 month AFTER their iNKT cells have returned to detectable levels
- If highest dose level is reached without determining a MTD, then the RDL for Phase 2 will be the dose that is at or below the highest dose and which iNKT cell recovery will allow dosing every month or every other month in Phase 2 study

Table 3: Schedule of Evaluations

Evaluations	Screening pre-run-in (4 weeks prior to dosing)	Screening run-in (2 weeks prior to dosing)	Day 0 (Dosing)		Follow-up		
			Pre-dose	Time Post-dose	Days 1,2,3,7,14	Months 1,2,3, etc.	EOS 1 month post iNKT cell recovery
Informed Consent	X						
Medical/Surgical History[abc]	X						
Assessment of pre-existing signs and symptoms and AEs		X		X t = 6 hrs	X 7,14	X 1,2,3, etc.	X
Concomitant Medications		X	X	X t = 6 hrs	X 7,14	X 1,2,3, etc.	X
Vital Signs[d]	X		X	X	X 7,14	X 1,2,3, etc.	X
Weight		X					
Physical Examination	X		X				X
Physical Assessment				X t = 6 hrs	X 7,14	X 1,2,3, etc.	
12-lead ECG		X					X
QuantiFERON-Gold (TB test)	X						
Chest X-ray[e]	X						X
Hematology and Coagulation	X			X t= 6 hrs	X 14	X 1,2,3, etc.	X
Serum chemistry and liver function tests[f]	X			X t= 6 hrs	X 14	X 1,2,3, etc.	X

**Evaluations**	**Screening pre-run-in (4 weeks prior to dosing)**	**Screening run-in (2 weeks prior to dosing)**	**Day 0 (Dosing)**		**Follow-up**		
			Pre-dose	Time Post-dose	Days 1,2,3,7,14	Months 1,2,3, etc.	EOS 1 month post iNKT cell recovery
Blood for peripheral lymphocytes by flow cytometry (FACS) assay[g]	X	X	X	X t= 6 hr	X 1,2, 7, 14	X 1,2,3, etc.	X
Blood for serum and cellular biomarkers[h]			X	X t = 6 hrs	X 7, 14	X 1,2,3, etc.	X
Serum for PK[i]			X	X t= 15 min, 30 min, 1 hr, 3 hr, 6 hr	X 1,2, 3, 7, 14	X 1,2,3, etc.	X
Serum for ADA			X				X
Urine (clean catch) for UA/micro and PCR[j]		X			X 14		X
Pregnancy Test[k]	X		X (just prior to dosing)				
QoL Assessments[l]		X				X 1,2,3, etc.	X
In-clinic PFTs		X		X		X 1,2,3, etc.	X
Daily eDiary		X[m]	X[n]	X	X[o]	X 1,2,3,4, etc.	X compliance

Evaluations	Screening pre-run-in (4 weeks prior to dosing)	Screening run-in (2 weeks prior to dosing)	Day 0 (Dosing)		Follow-up		
			Pre-dose	Time Post-dose	Days 1,2,3,7,14	Months 1,2,3, etc.	EOS 1 month post iNKT cell recovery
Administration of study drug				X<br>t = 0			

[a] Should ensure subject meets all inclusion criteria and no exclusion criteria
[b] SCD history includes frequency of vaso-occlusive crisis, history of acute chest syndrome, other known organ involvement/damage, history of transfusions, recent transfusions
[c] Immunization history, see Section 18.1
[d] Just prior to dosing, on Day 0, then every 15 minutes post dose for 1 hour or until stable, then every 30 minutes post infusion.
[e] PA and lateral to rule out TB or other pulmonary disease process
[f] Blood glucose, creatinine, uric acid, blood urea nitrogen (BUN), sodium potassium chloride, calcium, phosphorus, total protein, albumin, globulin, total bilirubin, direct bilirubin, alkaline phosphatase, lactic acid dehydrogenase (LDH), aspartate aminotransferase (AST or SGOT), alanine aminotransferase (ALT or SGPT) and CRP
[g] iNKT cell subsets, NK cells, total T lymphocytes, B cells, iNKT cell activation marker (CD69)
[h] INFγ, F1+2, D-dimers, vWF and cytokines including INFγ
[i] Two aliquots for PK should be prepared (one for current analysis and one for retained). Retained samples may be used to re-test PK at end of study or for other serum test such as autoantibodies, serum cytokines or other tests required to evaluate AEs or mechanism of action of NKTT120.
[j] If dip is abnormal, proceed to microanalysis.
[k] Serum pregnancy test will be performed at screening pre-run-in and urine pregnancy test at Day 0 just prior to dosing.
[l] Will be administered in clinic prior to any other assessments. Instruments include PROMIS and ASCQ-Me
[m] Provide subject with eDiary demonstration
[n] Assess compliance with daily eDiary
[o] Restart daily eDiary on Day 2 post-dosing (assess pain, medication use, hospital/medical utilization for previous day)

7. SELECTION AND WITHDRAWAL OF SUBJECTS

The inclusion and exclusion criteria will be confirmed by the Investigator or an appropriate designee of his/her study staff. Subjects enrolled into the study must meet all of the following inclusion criteria and will be excluded from participation if they meet any of the exclusion criteria. “Stable” disease is defined by exclusion criteria in Section 7.2 .

7.1. Subject Inclusion Criteria

1. Adults 18-50 years of age
2. Willing and able to sign informed consent
3. Diagnosed with SCD, either HbSS or HbS/B^0 thalassemia, based on hemoglobin analysis
4. Have measurable baseline iNKT cells determined by recognition on FACS assay of circulating peripheral blood iNKT cells measured at screening pre-run-in visit and again at the screening-run-in visit
5. Have stable SCD defined as not having experienced acute pVOC, ACS or other major or significant SCD-associated event requiring hospitalization or outpatient medical care during the month prior to enrollment.

7.2. Subject Exclusion Criteria

1. SCD-related pVOC or ACS that required hospitalization or treatment in an emergency or acute care outpatient setting within one month prior to enrollment. Painful crises are defined as occurrence of pain in the extremities, back, abdomen, chest or head that lasted at least two hours, required medical treatment and could not be explained except by SCD or as pain of the type the subject usually associates with a painful crisis. Definition of painful crisis excludes episodes of pain treated entirely at home.
2. Require a program of prescheduled regularly administered chronic blood transfusion therapy, or be expected to receive a transfusion during enrollment on the study. Subjects are not eligible if they have received a packed red blood cell (PRBC) transfusion within 4 weeks of enrollment.
3. Experienced more than ten painful crises for which medical treatment was received in the twelve months prior to enrollment.
4. Change in medications or doses of medications prescribed for SCD (e.g., hydroxyurea, oral antibiotic prophylaxis, pain medications) or medications prescribed for concomitant medical conditions (e.g., antihypertensives, medications to control hypercholesterolemia, vitamin D, calcium supplements, birth control, SABA, medications to control neuropathic pain)) ≤ 2 months prior to enrollment
5. Moderate-to-severe asthma or taking asthma medications other than inhaled SABA (i.e., step 2 or higher level asthma medications) to relieve symptoms
6. Use of corticosteroids (oral, parenteral, or inhaled) > 10 mg/day prednisone equivalent ≤ 2 months prior to enrollment

7. Hematology values outside of protocol-defined ranges at screening, including:
    - Hemoglobin ≥ 6 g/dL
    - Platelets ≤ 150 x 10^9/L
    - Lymphocytes < 1.0 x 10^9/L
    - Absolute neutrophils ≤ 2 x 10^9/L
    - PT or aPTT outside of normal range
8. Serum chemistry and liver function test values outside of protocol-defined ranges at screening, including:
    - ALT (SGPT) ≥ 2.5 x ULN
9. Serum creatinine ≥ 1.5 mg/dL and spot urine protein/creatinine ratio ≤ 0.5 mg/mg
10. Evidence of current infection requiring ongoing treatment
    - Evidence of latent or active mycobacterium TB determined by positive QuantiFERON-Gold test or screening CXR, or lack of documentation of adequate treatment of prior infection with mycobacterium TB or history of infection with atypical mycobacterium
    - History of viral infection(s) requiring ongoing treatment (e.g., HIV, HCV, HBV, HSV type 2)
    - Received any live or attenuated viral immunization within six months prior to enrollment
11. Subject is not up-to-date on CDC-recommended immunizations for adults (see 18.1) or received immunization within one month prior to enrollment
12. Major concurrent illness or medical condition that, in the opinion of the investigator, would preclude participation in a clinical study, including but not limited to:
    - Uncontrolled significant cardiovascular disease or cardiac arrhythmia or clinically relevant abnormal screening ECG
    - Serious event such as stroke, TIA, DVT or PE ≤ 6 months prior to enrollment
    - Significant bleeding of any cause ≤ 6 months prior to enrollment
    - Psychiatric, behavioral or addictive disorder that, in the opinion of the investigator, would preclude participation in a clinical study
    - Known severe pulmonary hypertension (tricuspid regurgitent jet velocity (TRV) ≥ 3.0 m/sec on 2-D echocardiogram or an estimated pulmonary artery systolic pressure ≥ 40 mmHg
    - Known severe CNS vasculopathy
    - Interstitial lung disease requiring continuous oxygen or clinically significant abnormal screening CXR or O_2 saturation < 90% on room air
13. Type 1 or type 2 diabetes

14. History of cancer
15. Known allergy or hypersensitivity to any component of NKTT120 manufacturing or formulation or has previously experienced adverse reaction to protein or biologic therapeutic or experimental agent
16. Pregnant or nursing
17. Subjects of either gender who are not using highly effective method of contraception while on study [Defined as established use of oral, injected or implanted hormonal methods of contraception or placement of an intrauterine device or intrauterine system or barrier methods of contraception such as condom or occlusive cap (diaphragm or cervical/vault caps) with spermicidal foam/gel/cream/suppository* by subject and/or partner of subject. Also acceptable are males with appropriate post-vasectomy documentation of the absence of sperm and women who are post-menopausal or permanently sterilized (e.g., tubal occlusion, hysterectomy or bilateral salpingectomy)]

    *The use of barrier contraceptives should always be supplemented with the use of a spermicide. Spermicides are not a barrier method of contraception and should not be used alone.
18. Subject has participated in a clinical trial and received an investigational product within one month, five half-lives or twice the duration of the biochemical or biological effect (whichever is longer) of the investigational product prior to the start of screening for this study
19. History of marrow or stem cell transplant

7.3. Subject Withdrawal Criteria

7.3.1. Withdrawal of Consent

Subjects are free to withdraw consent and discontinue participation in the study at any time without prejudice to further treatment. Withdrawal of consent should be in writing. Subjects who withdraw consent immediately after treatment will be asked to remain in the study for safety follow-up for at least 30 days after dosing. These subjects and other subjects who withdraw consent during the follow-up period will be asked to complete the end-of-study (last visit) assessment prior to discharge from the study. Subjects who are enrolled, sign informed consent, but do not receive NKTT120, will be replaced.

7.3.2. Failure to Return for Follow-Up

Subjects who do not return for follow-up will be considered lost to follow-up. The appropriate clinical site personnel should attempt to contact these subjects by telephone, mail or email in order to obtain information about the reason(s) for discontinuation and information about potential AEs.

The Investigator will provide a written report, collect all EOS data and will complete the Subject Completion/Discontinuation Section of the eCRF.

8. TREATMENT OF SUBJECTS

8.1. Description of Study Drug

Table 4: Investigational Product

	**Investigational Product Description**
**Product Name:**	NKTT120
**Dosage Form:**	10/mg/mL in 20 mM sodium acetate buffer, pH 5.0, containing 6% trehalose and 0.02% Tween-80
**Route of Administration**	IV
**Physical Description**	Clear, colorless solution, essentially free of particulates
**Manufacturer**	DSM Biologics B.V.

8.2. Concomitant Medications

8.2.1. Required Medications

The following medications are required for participation in the clinical study:

1. Immunizations recommended by CDC for adults, unless contraindicated for subject (18.1)
2. Birth control: Barrier (condom or diaphragm) plus spermicide, intrauterine device (hormonal or copper); or oral (combination or single agent) or injectable/implanted hormonal contraceptive

8.2.2. Permitted Medications

All concurrent medications prescribed for SCD or other medical conditions must be at stable doses for at least three months prior to enrollment the study. The following medications are permitted but not required during the study:

- Hydroxyurea (stable dose for two months prior to dosing)
- Antibiotic prophylaxis
- Vitamin D
- Calcium supplement
- Medications for treatment of hypercholesterolemia or hypertriglyceridemia (i.e., statins)

- Pain medications including: NSAID, non-narcotic analgesics, short-acting opioids, long-acting opioids
- Anti-hypertensives: e.g., angiotensin converting enzyme inhibitors, aldosteronereceptor blockers
- Inhaled SABA
- Corticosteroid (oral or inhaled), prednisone (or prednisone equivalent), 10 mg/day.
- Therapies to treat neuropathic pain such as: antidepressants (amitriptyline and nortriptiline), anticonvulsants (gabapentin (Neurontin®) pregabalin (Lyrica®), carbamazepine, felbamate, valproic acid, clonazepam and phenytoin)
- Medications to treat study-emergent adverse events

8.2.3. Prohibited Medications and Treatments

The following medications and treatments are prohibited for the duration of the trial:

- Routinely scheduled blood transfusions as part of chronic blood transfusion therapy
- Erythropoietin
- Corticosteroids (oral, parenteral or inhaled), greater than 10 mg/day prednisone or prednisone equivalent)
- Any experimental agent or drug
- Any broadly immunosuppressive drug or biologic
- Homeopathic medications, unless approved by the investigator

8.3. Randomization and Blinding

This is an open-label study. No randomization or blinding will be required.

9. STUDY DRUG MATERIALS AND MANAGEMENT

9.1. Study Drug

NKTT120 is a novel recombinant, humanized, IgG1κ mAb that selectively binds to the human iTCR on a subset of T cells called iNKT cells. NKTT120 binds exclusively and specifically to Vα24-Jα18 gene-rearranged iTCRs on human iNKT cells, resulting in depletion of iNKT cells.

NKTT120 is formulated as a clear colorless solution, essentially free of particulates, at a concentration of approximately 10 mg/mL in 20 mM sodium acetate buffer, pH 5.0, containing 6% trehalose and 0.02% Tween-80. All excipients are generally recognized as safe (GRAS) and found in other FDA-approved IV products at equivalent or higher concentrations.

NKTT120 is aseptically filled into USP Type 1 clear glass vials, each closed with a polymer-coated butyl rubber stopper and capped with aluminum caps. Each 3 mL vial of NKTT120 contains 1 mL drug.

This product is for single-use only and will not contain a preservative. It should be stored frozen at -20° C and thawed just prior to use or thawed in refrigerator overnight. The thawed vial should be used for preparation of dose within two hours if kept at room temperature and within 24 hours if refrigerated. Prepared doses must be infused immediately after preparation. Refer to the Pharmacy Manual for further drug preparation and dosing information.

9.2. Study Drug Packaging and Labeling

The packaging and labeling will be performed by the Sponsor's packaging and labeling Vendor according to all local legal and regulatory requirements.

Each box will contain between six and nine vials. The number of vials in each box will be identified on the box label. The box label will also indicate the Retest Date of drug contained within the box. The Retest Date will be revised annually based on data gathered from an ongoing stability study.

Refer to the Pharmacy Manual for a description of the drug vial and carton labels.

9.3. Study Drug Storage

Study drug will be shipped directly to each clinical site from the NKT Therapeutics Inc. distribution center.

NKTT120 must arrive frozen at the clinical trial site. Upon receipt of a shipment of NKTT120, the site staff will immediately inspect the condition of the shipping containers and the vials to ensure the shipping container has no damage, the gel packs are frozen, the vials are not damaged and TempTale®4 is not alarmed during shipping. If any of the vials are damaged or have thawed during shipment, the site will document the damage on the drug accountability record and notify NKTT immediately of the problem. **Damaged or defrosted vials cannot be used for the study. Authorization and instructions for destruction and/or return of the damaged Study Drug will be provided in writing by NKTT.** Refer to the Pharmacy Manual for detailed instructions.

Upon receipt, the vials of NKTT120 should be stored upright in a monitored -20°C freezer until required for use.

9.4. Study Drug Preparation

NKTT120 is a liquid formulation and reconstitution is not required. In preparation for use as an IV infusion, NKTT120 will be diluted in sterile normal saline (NS). Preparation of NKTT120 for IV infusion should be performed by the clinical study pharmacist using the Pharmacy Manual.

9.5. Administration

Study drug should be administered according to the study specific instructions detailed in the Pharmacy Manual.

Should the subject experience any adverse event during the infusion, the infusion rate should be slowed or stopped according to the discretion of the investigator until the symptom has resolved or the Subject stabilized (see Pharmacy Manual for details). If restarted, the infusion should be slowed according to the discretion of the investigator (see Pharmacy Manual for details).

Should CRS of any type be observed in subjects in any cohort, premedication with acetaminophen (1000 mg), histamine 2 receptor antagonists (diphenhydramine, 50 mg) and corticosteroids may be initiated prior to dosing of subsequent subjects to lessen severity of CRS.

9.6. Study Drug Accountability

Drug accountability records will be maintained for all clinical trial supplies. The study center will complete the drug accountability records to document the receipt, dispensing and return or destruction of study drug (NKTT120 vials) throughout the study. NKTT personnel or representatives will monitor the completeness and accuracy of all accountability records throughout the course of the study. NKTT or designee will verify product accountability against site documentation and all study product, used and unused. Unused materials will be destroyed at the site according to the site's procedure or returned after reconciliation by the study monitor according to the written instructions provided by NKTT.

9.7. Study Drug Handling and Disposal

10.7.1 Handling Instructions

NKTT120 is provided as a 1 mL sterile solution in a single use, glass vial at a nominal concentration of 10 mg/mL and is formulated as described in the section above (Section 9.1).

NKTT120 must be stored upright and frozen at temperature between -20° C (-14 and -26 °C or 7 and 15 °F). It must be stored in the original carton until time of use.

10.7.2 Disposal Instructions

At the end of the study any unused drug will be destroyed at the site according to the site's standard operating procedures. Study drug will not be returned unless instructed to do so by NKTT.

If the study drug is to be destroyed at the site, it is the investigator's responsibility to ensure that arrangements have been made for the disposal and procedures for proper destruction have been

established according to applicable regulations, and institutional procedures. Appropriate records of the destruction must be documented and maintained by the site.

The unused or damaged study drug may only be destroyed after inspection and reconciliation by the responsible NKTT CRA or representative. A copy of the destruction record will be kept in the study files at the site and copies will be maintained in the study master files.

If study drug (NKTT120) is to be returned to the NKTT, it must be accompanied by the appropriate documentation and be clearly identified by the protocol number, study site number and Principal Investigator name. Returned drug should be in the original container. Detailed instructions and contact information are provided in the Pharmacy Manual.

10. PHARMACOKINETIC ASSESSMENTS

10.1. Blood Sample Collection

Blood for PK sampling will be collected at the following time points (see Table 3): day 0 pre-dosing and post-dose 15 minutes, 30 minutes, 1 hour, 3 hours and 6 hours after end of infusion; day 1, day 2, day 3, day 7, day 14; and at each monthly visit, including the EOS visit. Times of start of infusion and end of infusion will be recorded and the actual time each PK sample was drawn will be recorded, using a 24 hour clock. Samples will be processed in the serum, stored frozen at -70ºC. Samples will be analyzed after completion of each cohort. Sites that cannot store samples at -70ºC will store samples at -20ºC and will ship samples to the Blood Center of Wisconsin within 14 days of collection.

10.2. Sample Analysis

Serum samples for PK analysis will be shipped to the Blood Center of Wisconsin laboratory for analysis. Samples will be analyzed using a validated ELISA for detection of NKTT120 in human serum. Refer to the laboratory manual for shipping details.

11. ASSESSMENT OF SAFETY

11.1. Safety Parameters

All adverse Events of Interest (EOI, below) that could be dose-limiting toxicities will be reviewed immediately by the SRC to determine if they constitute a DLT. All serious adverse events at any time will be reviewed by the SRC so that the SRC can make recommendations to the Sponsor regarding dosing cohorts. All safety data, including adverse events and laboratory abnormalities, will also be reviewed by the SRC periodically in order to assist the sponsor in evaluating potential safety signals during the follow-up until iNKT cells recover.

Event of interest (EOI) Criteria: an EOI is defined only for adverse events that occur within 2 weeks of dosing (with the exception of any acute vaso-occlusive episode). Any adverse event grade 3 or higher on NCI CTCAE version 4.03 will be considered an EOI. Additionally, the following study and disease-specific toxicities will be considered an EOI.

- **Cytopenias:** Defined as CTCAE version 4.03 Grade 3 or 4 leukopenia, neutropenia, lymphopenia or thrombocytopenia.
- Specific grade 3 definitions in CTCAE version 4.03 are:
  - Leukopenia: <2000 cells/cubic milliliter
  - Neutropenia: <1000 cells/cubic milliliter
  - Lymphopenia: <500 cells/cubic milliliter
  - Thrombocytopenia: < 50/microliter
- **Infections:** Defined as CTCAE version 4.03 grade 3 or 4. Specific infections are detailed in the CTCAE. Broadly the definition is a severe infection that requires IV antibiotic, antifungal or antiviral therapy, or otherwise severe and medically significant, but not necessarily life-threatening.
- **Cytokine storm syndrome:** Defined as CTCAE version 4.03 grade 3 or 4. The grade 3 definition reads: Prolonged (eg, not rapidly responsive to symptomatic medication and/or brief interruption of infusion); recurrence of symptoms following initial improvement; hospitalization indicated for clinical sequelae (eg, renal impairment or pulmonary infiltrates)
- **Sickle cell-specific dose limiting toxicities:**
  - Hemolysis:
  - Anemia: >25% decrease in hemoglobin from baseline or ≤ 5.0 g/dL
  - Lactate dehydrogenase increase 3-fold from baseline
  - Reticulocyte count increase 3-fold from baseline
  - AST increase 3-fold from baseline
  - Total bilirubin increase 3-fold from baseline

- **Acute vaso-occlusive episode:** within the 6-hour monitoring window following infusion, an increase in pain over baseline (≥ 4 points on a 10 point pain scale) in the extremities, chest, abdomen or head lasting for at least 2 hours that could not be explained except by sickle cell disease.
- **Increased pain in the extremities, chest, abdomen or head lasting for at least 2 hours:** Within 72 hours following infusion, a provider encounter (clinic, ED or hospital), requiring opioid administration and could not be explained except by sickle cell disease.

11.1.1. Demographic/Medical History

Medical history will be obtained at screening pre-run-in to determine if there are any clinically significant diseases or medical problems, other than SCD, that would affect the subject's ability to comply with study visits or procedures. This history must include documentation of the subject's sickle cell disease, including dates of diagnosis and history of treatments, as well as all previous surgeries, immunizations and medications, with dates of diagnosis and treatment. A complete allergy history, including diagnosis and treatment, must also be documented.

11.1.2. Vital Signs

Vital signs, including blood pressure, heart rate and oral body temperature (°C) will be measured at screening pre-run-in, immediately prior to dosing, at regular frequent intervals on day 0, and at each follow-up visit, as outlined below.

- On Day 0 every 15 minutes for 1 hour or until stable, then every 30 minutes post-infusion
- On days 7 and 14, and at monthly follow-up visits or at any unscheduled visit using the standardized process:
  - The subject should sit for five minutes with feet flat on the floor and his/her measurement arm supported so that the midpoint of the manometer cuff is at heart level
  - A mercury sphygmomanometer or automatic blood pressure device with an appropriately-sized cuff with the bladder centered over the brachial artery should be used
- Blood pressure and heart rate should be measured and recorded

Blood pressure should be recorded to the nearest 2 mm Hg mark on the manometer or to the nearest whole number on an automatic device.

11.1.3. Weight

Weight (kg) will be measured at screening run-in. Subjects should be weighed wearing indoor, daytime clothing with no shoes. Before being weighed, subjects should empty their bladder.

11.1.3.1. Physical Examination/Physical Assessment

A physical examination directed toward subject medical history and current disease (consisting of head, eyes, ears, nose, throat, respiratory, gastrointestinal, extremities, musculoskeletal, cardiovascular, nervous system, lymph nodes, abdomen, dermatologic, and other physical conditions of note) will be performed at screening pre-run-in, prior to dosing at Day 0, and at the EOS visit (Table 3).

A physical assessment is a physical exam performed by someone other than the Investigator or sub-Investigator (this may be a Registered Nurse or Physician's Assistant), the purpose of which is to determine general physical health of the subject and any changes from prior visits. All other physical examinations will be symptom-based or system-directed to evaluate adverse events or subject status. Physical assessments will be performed 6 hours after the infusion, on days 7 and 14 and monthly until EOS visit.

11.1.4. Electrocardiogram (ECG)

A 12-lead ECG will be performed at screening run-in and at the EOS visit, after the subject has been in the supine position for at least 10 minutes. The ECG will include all 12 standard leads and will be recorded at a paper speed of 25 mm/sec. Standard ECG parameters will be measured, including RR, PR, QTc interval, and QRS duration. All ECGs must be evaluated by a qualified physician for the presence of abnormalities.

11.1.5. Laboratory Assessments

Unexpected abnormal laboratory results will be defined as AEs and graded per CTCAE v. 4.03 criteria. All abnormal laboratory values will be reviewed by the investigator, who will determine clinical significance and relationship to NKTT120. Significantly out-of-range values will be flagged for rapid review by the investigator.

11.1.5.1. Hematology

Hematology laboratory assessments will be performed at screening pre-run in, 6 hours post dose on Day 0, on day 14 and at each monthly follow-up visit, including the EOS visit. Hematology lab assessments include WBC, absolute and percent differential WBC, platelet count, RBC, hemoglobin, reticulocyte number and percent.

11.1.5.2. Blood Chemistry

Chemistry laboratory tests will be performed at screening pre-run in, 6 hours post dose on Day 0, on day 14, and at each monthly follow-up visit, including the EOS visit. Each test will include blood glucose, creatinine, uric acid, blood urea nitrogen (BUN), sodium, potassium, chloride, calcium, phosphorus, total protein, albumin, globulin, total bilirubin, direct bilirubin, alkaline phosphatase, lactate dehydrogenase (LDH), aspartate aminotransferase (AST or SGOT), alanine aminotransferase (ALT or SGPT), and C-reactive protein (using a high-sensitivity assay).

11.1.5.3. Urinalysis and Urine Protein/Creatinine Ratio

Urine will be collected as clean-catch at screening run-in, day 14 and EOS. Urinalysis will be performed by dip test and, if abnormal, microanalysis will be performed.

11.1.5.4. Coagulation

Coagulation assessments as a safety measure will be measured by PT and aPTT and will be performed according to Table 3 and at the same time points as Hematology (11.1.5.1). Platelet measurements will be done routinely for safety as part of a complete blood count.

Additional measurements coagulation assessments and measures of endothelial activation will be performed, but these will be exploratory analysis only, and not be analyzed as part of the safety evaluation.

11.1.5.5. Pregnancy Screen

Women of child-bearing potential will be screened for pregnancy at screening pre-run-in (blood test) and prior to administration of study drug (urine test). If the results of the serum pregnancy test at screening pre-run-in are positive, the subject will be excluded. Prior to dosing on Day 0, a urine pregnancy test will be performed to confirm the female subject is not pregnant. If the urine pregnancy test is positive, then the subject will be withdrawn from the study. Results of the pregnancy tests must be recorded in the subject's source documents (medical chart).

11.2. Adverse and serious adverse events

11.2.1. Definition of adverse events

11.2.1.1. Adverse event (AE)

An AE is the development of an undesirable medical condition or the deterioration of a pre-existing medical condition following or during exposure to a pharmaceutical product, whether or not considered casually related to the product. In clinical studies, an AE can include an undesirable medical condition occurring at any time, including baseline or washout periods, even if no study treatment has been administered.

Event of interest (EOI) criteria: an EOI is defined only for adverse events that occur within 2 weeks of dosing (with the exception of any acute vaso-occlusive episode). Any adverse event grade 3 or higher on NCI CTCAE version 4.03 will be considered an EOI, and a determination of whether it constitutes a DLT will be made by the SRC.

All baseline signs and symptoms and AEs that occur after any subject has been enrolled, before treatment, during treatment, or for 30 days following the recovery of iNKT cells, whether or not they are related to the study, must be recorded on forms provided by NKTT.

11.2.1.2. Serious adverse event (SAE)

A serious adverse event is an AE occurring during any study phase (i.e., baseline, treatment, washout, or follow-up), and at any dose of the investigational product, comparator or placebo, that fulfills one or more of the following:

- Results in death
- It is immediately life-threatening
- It requires in-subject hospitalization or prolongation of existing hospitalization
- It results in persistent or significant disability or incapacity

- Results in a congenital abnormality or birth defect
- It is an important medical event that may jeopardize the subject or may require medical intervention to prevent one of the outcomes listed above.

All SAEs that occur after any subject has been enrolled, before treatment, during treatment, or within 30 days following the recovery of iNKT cells, whether or not they are related to the study, must be recorded on forms provided by NKT Therapeutics Inc.

11.2.1.3. Other adverse event (OAE)

OAEs will be identified by the Drug Safety Physician during the evaluation of safety data for the Clinical Study Report. Significant adverse events of particular clinical importance, other than SAEs and those AEs leading to discontinuation of the subject from the study, will be classified as OAEs. For each OAE, a narrative may be written and included in the Clinical Study Report.

11.3. Relationship to study drug

An Investigator who is qualified in medicine must make the determination of relationship to the investigational product for each AE (Unrelated, Possibly Related or Probably Related). The Investigator should decide whether, in his or her medical judgment, there is a reasonable possibility that the event may have been caused by the investigational product. If no valid reason exists for suggesting a relationship, then the AE should be classified as “unrelated.” If there is any valid reason, even if undetermined, for suspecting a possible cause-and-effect relationship between the investigational product and the occurrence of the AE, then the AE should be considered “related.”

If the relationship between the AE/SAE and the investigational product is determined to be “possible” or “probable” the event will be considered to be related to the investigational product for the purposes of expedited regulatory reporting.

For the purposes of this study, a disease-specific adverse event is an adverse event or outcome that is associated with SCD. Examples include, but may not be limited to: hemolysis, hepatic/splenic sequestration, haptoglobin decrease, hemoglobinuria, pain crisis, bone pain, chest wall pain, pain in extremity, priapism in men, acute chest syndrome, gall-stones, leg ulcers, avascular necrosis of bone, stroke, cognitive disabilities. These events will be captured as AE and/or SAEs in the CRF. Disease specific AEs not thought to be related to NKTT120 will not be considered dose-limiting events.

11.4. Recording adverse events

Adverse events spontaneously reported by the subject and/or in response to an open question from the study personnel or revealed by observation will be recorded during the study at the investigational site. Clinically significant changes in laboratory values, blood pressure, and pulse need not be reported as AEs. However, abnormal values that constitute an SAE must be reported and recorded as an AE. Information about pre-existing signs and symptoms will be collected from the time the subject signs an informed consent form. AEs will be collected from the first administration of study drug until the end of the study. SAE information will be collected from administration of study drug until their EOS visit, which is 30 days following the recovery of iNKT cells in the peripheral circulation. The AE term should be reported in standard medical

terminology when possible. For each AE, the investigator will evaluate and report the onset (date and time), resolution (date and time), intensity, causality, action taken, serious outcome (if applicable), and whether or not it caused the subject to discontinue the study.

Intensity will be assessed according to the CTCAE grading scale version 4.03.

It is important to distinguish between serious and severe AEs. Severity is a measure of intensity whereas seriousness is defined by the criteria in Section 11.2.1.2. An AE of severe intensity may not be considered serious.

In principle, pregnancy and the lactation period are exclusion criteria for clinical studies involving investigational drugs that are not directly related to the respective conditions. Should a pregnancy occur during this study, it must be reported and recorded on the pregnancy form and the subject should be followed in the study until one month after recovery of iNKT cells and during the entire course of the pregnancy and postpartum period. All recommendations described in the Investigator Brochure pertaining to pregnancy and lactation must be carefully considered. Pregnancy in itself is not regarded as an AE unless there is a suspicion that an investigational product may have interfered with the effectiveness of a contraceptive medication.

The outcome of all pregnancies (spontaneous miscarriage, elective termination, normal birth or congenital abnormality) must be followed up and documented even if the subject was discontinued from the study.

All reports of congenital abnormalities and birth defects are SAEs. Spontaneous miscarriages should also be reported and handled as SAEs. Elective abortions without complications should not be handled as AEs.

11.5. Reporting adverse events

All SAEs (related and unrelated) will be recorded from the or first administration of study drug until the EOS visit. Any SAEs considered possibly or probably related to the investigational product and discovered by the Investigator at any time after the study should be reported. All SAEs must be reported to the CRO within one business day of the first awareness of the event.

Additional follow-up information, if required or available, should all be faxed to the CRO within one business day of receipt and this should be completed on a follow-up SAE form and placed with the original SAE information and kept with the appropriate section of the CRF and/or study file.

NKTT is responsible for notifying the relevant regulatory authorities of reportable events. It is the Principal Investigator's responsibility to notify the IRB of all SAEs that occur at his or her site. Investigators will also be notified of all unexpected, serious, drug-related events (7/15 Day Safety Reports) that occur during the clinical trial. Each site is responsible for notifying its IRB of these additional SAEs.

12. STATISTICS

The primary endpoint of this study is the identification of a safe and tolerable IV dose of NKTT120 in subjects with SCD to be taken forward in phase 2 studies of SCD subjects.

Secondary endpoints of this study are laboratory assessments based on peripheral blood to evaluate the PK of NKTT120 and to identify iNKT cell numbers and markers of activation. Pulmonary function, immunogenicity profile, QoL domains, use of analgesics, and pain score profile in subjects receiving NKTT120 will also be examined. For all studies, serial changes from baseline within subjects will be evaluated.

Subjects who sign informed consent, completed all pre-screening requirements and were determined to be eligible, but are not dosed, will be replaced.

Subjects who are enrolled and dosed will define the Safety Population.

Subjects, who are enrolled, dosed, and complete the two week evaluability period for DLTs, will define the DLT-evaluable population.

Completed subjects are defined as those subjects who continue to return to the clinic for visits until their iNKT cells recover and return for their EOS visit.

Escalation scheme

Five dose levels of NKTT120 will be assessed. Each dose level will be administered as a single ascending IV dose. The dose levels to be investigated are 0.001, 0.003, 0.01, 0.03 and 0.1 mg/kg.

A standard dose escalation Fibonacci 3+3 design will be employed. Initially, three subjects will be treated. If no subject experiences DLT, dosing will escalate. If one subject experiences DLT, 3 additional subjects will be treated at the current dose and escalation will occur only if no more than 1 of 6 subjects experiences DLT. If ≥ 2 of 3 - 6 subjects experiences DLT at the initial dose level of 0.001 mg/kg, then it may be de-escalated. The study continues until MTD has been determined, or the highest dose cohort is filled. If no MTD is reached, RDL will be determined based on the overall safety profile of NKTT120 and the duration of iNKT cell depletion. The RDL(s) for phase 2 will be determined by a dose that is at or below the highest dose tested which is associated with timing of iNKT cell recovery that allows dosing every 4-6 months in phase 2 studies. The dose-level cohort that represents the RDL will be expanded to 6 subjects to confirm the RDL. Table 5 below provides the probability of dose escalation.

Table 5: Statistical Properties of the Study Design

	DLT	Pr(escalate)
True but unknown probability of DLT	0.05	0.973
	0.10	0.906
	0.15	0.814
	0.20	0.709
	0.25	0.600
	0.30	0.494
	0.40	0.309
	0.50	0.172
	0.80	0.009
	0.09	0.001

DLTs are defined as in 6.6.1. Subjects will be observed for 2 weeks following dosing to fully assess DLT (with the exception of any acute vaso-occlusive episode).

Analysis of Secondary Endpoints

Demographics and baseline status will be documented by dose level and/or overall, as applicable.

12.1. Pharmacokinetics and pharmacodynamics

Non-compartmental pharmacokinetic parameters (C_{max}, C_{min}, C_{avg}, AUC, $t_{½}$, V_d) will be calculated for all subjects. Descriptive statistics of these parameters will be provided by dose level.

Descriptive statistics of the PK/PD (peripheral iNKT cell:T cell ratio, depletion and recovery to quantifiable levels) will be provided by dose level. PK/PD will be correlated/associated with AEs, pain scores and other diary endpoints, and changes in biomarkers.

Binary criteria have been established for activity for the secondary endpoints. Decrease in iNKT cell number and/or markers of activation by 75% will be examined to assess the impact of infusional NKTT120 on these endpoints. These results will be presented as point estimates of proportion of subjects achieving target reduction, with 90% exact binomial confidence intervals, for subjects within each of the dose level cohorts of the study. Mixed models will also be explored to elucidate the tempo of changes in iNKT cell number and markers of activation, controlling for subject and for cohort of study as well as other potential covariates.

12.2. Clinical measures of SCD and biomarker analysis

Changes in leukocyte counts, hemoglobin, reticulocytes, platelets, LDH, and CRP for each subject will be compared to baseline values and changes will be presented as change tables and described.

Changes from baseline in serum concentrations of IFNγ, TNFα, IL-1β, IL-6, IL-8 , F1+2, D-dimers, vWF, $sPLA_2$ and sP-selectin will be presented as change score.

12.3. Pain, analgesic use and QoL

Pain frequency and intensity, and use of analgesic medications to treat SCD pain will be recorded by the subject in a daily eDiary. The duration of pain and number of subject reported "pain crisis" days will be determined from the eDiary. Post-dosing values will be compared to run-in values. If the subject does not have daily access to such a phone, s/he will be provided with a paper version of the application and encouraged to record his/her daily ratings on paper.

For questionnaires for which the study developers have provided guidelines for scoring in the presence of missing data, those guidelines will be followed. For other instruments, the number of questionnaires will be tallied for which a particular data item is missing.

12.4. Pulmonary Function

PFTs in clinic include forced vital capacity (FVC), Forced expiratory volume in 1 second (FEV_1), Forced expiratory flow 25%-75%, (FEF_{25-75}) and peak expiratory flow (PEF) and will be performed in the clinic at screening run-in, after dosing on Day 0, monthly and EOS. Run-in and post dosing values will be correlated/associated with PK/PD for each dose cohort.

An improvement in FEV_1or FEV_1/FVC by 20% will be examined to assess the impact of infusional NKTT120 on these endpoints and these results will be presented as point estimates of proportion of subjects achieving target reduction, with 90% confidence intervals, for subjects within each of the dose level cohorts of the study. Mixed models will be explored to elucidate the tempo of changes in pulmonary function, controlling for subject and for cohort of study, as well as other potential covariates.

12.5. Immunogenicity Analysis

The presence of ADA will be described as present or absent at each test. The percentage of positive tests will be reported for each dose cohort and the study as a whole.

12.6. Sample Size

Sample size is determined by the 3+3 study design and the number of dose-level cohorts (up to 5 cohorts planned). Approximately 30 subjects will be dosed in the trial, with three to six subjects per cohort. The minimum sample size is 2 subjects. Enrollment through follow-up is estimated to require 18 months. Subjects who are enrolled, sign informed consent but do not receive NKTT120, will be replaced.

12.7. Reporting and Exclusions

Evaluation of toxicity will be as follows:

All subjects who initiate infusion with NKTT120 will be evaluable for adverse events from the time of enrollment.

13. DIRECT ACCESS TO SOURCE DATA/DOCUMENTS

13.1. Study Monitoring

Before an investigational site can enter a subject into the study, a representative of NKTT will visit the investigational study site to:

- Determine the adequacy of the facilities
- Discuss with the investigator(s) and other personnel their responsibilities with regard to protocol adherence, and the responsibilities of NKTT or its representatives. This will be documented in a Clinical Study Agreement between NKTT and the investigator.

During the study, a monitor from NKTT or sponsor representative will have regular contacts with the investigational site, for the following:

- Provide information and support to the investigator(s)
- Confirm that facilities remain acceptable
- Confirm that the investigational team is adhering to the protocol, that data are being accurately recorded in the electronic case report forms, and that investigational product accountability checks are being performed
- Perform source data verification. This includes a comparison of the data in the case report forms with the subject's medical records at the hospital or practice, and other records relevant to the study. This will require direct access to all original records for each subject (e.g., clinic charts)
- Record and report any protocol deviations not previously sent to NKTT
- Confirm AEs and SAEs have been properly documented on eCRFs and confirm any SAEs have been forwarded to NKTT and those SAEs that met criteria for reporting have been forwarded to the IRB

The monitor will be available between visits if the investigator(s) or other staff needs information or advice.

13.2. Audits and Inspections

Authorized representatives of NKTT, a regulatory authority, an Independent Ethics Committee or an Institutional Review Board may visit the site to perform audits or inspections, including source data verification. The purpose of an NKTT audit or inspection is to systematically and independently examine all study-related activities and documents to determine whether these activities were conducted, and data were recorded, analyzed, and accurately reported according to the protocol, Good Clinical Practice guidelines of the International Conference on Harmonization, and any applicable regulatory requirements. The investigator should contact NKTT immediately if contacted by a regulatory agency about an inspection.

13.3. Institutional Review Board (IRB)

The Principal Investigator must obtain IRB approval for the investigation. Initial and annual IRB approval, protocol amendment approval and all materials approved by the IRB for this study including the subject informed consent form and recruitment materials must be maintained by the Investigator and made available for inspection.

14. QUALITY CONTROL AND QUALITY ASSURANCE

To ensure compliance with Good Clinical Practices and all applicable regulatory requirements, NKTT or representative may conduct a quality assurance audit. Please see Section 13.2 for more details regarding the audit process.

15. ETHICS

15.1. Ethics Review

The final study protocol, including the final version of the Informed Consent Form, must be approved or given a favorable opinion in writing by an IRB as appropriate. The investigator must submit written approval to NKTT before he or she can enroll any subject into the study.

The Principal Investigator is responsible for informing the IRB of any amendment to the protocol in accordance with local requirements. In addition, the IRB must approve all advertising used to recruit subjects for the study. The protocol must be re-approved by the IRB upon receipt of amendments and annually, as local regulations require.

The Principal Investigator is also responsible for providing the IRB with reports of any reportable serious adverse drug reactions from all studies conducted with the investigational product. NKTT will provide this information to the Principal Investigator.

Progress reports and notifications of serious adverse drug reactions will be provided to the IRB according to local regulations and guidelines.

15.2. Ethical Conduct of the Study

The study will be performed in accordance with ethical principles that have their origin in the Declaration of Helsinki (Section 18.3) and are consistent with ICH/Good Clinical Practice, applicable regulatory requirements.

15.3. Written Informed Consent

The Principal Investigator(s) at each center will ensure that the subject is given full and adequate oral and written information about the nature, purpose, possible risk and benefit of the study. Subjects must also be notified that they are free to discontinue from the study at any time. The subject should be given the opportunity to ask questions and allowed time to consider the information provided.

All subjects (or their legally acceptable representative) must read, sign, and date a consent form before any assessments for participating in the study, taking the study drug, and/or undergoing any study-specific procedures. If a participant does not speak and read English, the consent materials must be translated into the appropriate language. A copy of the informed consent form will be given to the subject and the original will be placed in the subject's medical record if in accordance with the institution's standard operating procedures. An entry must also be made in the subject's chart to confirm that informed consent was obtained prior to any study-related procedures and that the subject received a signed copy.

The informed consent form must be updated or revised whenever important new safety information is available, whenever the protocol is amended, and/or whenever any new information becomes available that may affect participation in the trial.

A copy of the informed consent will be given to a prospective participant for review. The investigator, in the presence of a witness, will review the consent and answer questions. The

participant will be informed that participation is voluntary and that he/she may withdraw from the study at any time, for any reason.

The Principal Investigator(s) must maintain the original, signed Informed Consent Form. A copy of the signed Informed Consent Form must be given to the subject.

15.4. Confidentiality of Subject Records

Subject information collected in this study will comply with the standards for protection of privacy of individually identifiable health information as promulgated by applicable local/regional/national requirements for subject confidentiality (e.g., Health Insurance Portability and Accountability Act as mandated in Title 45 CFR, Parts 160 and 164). All records will be kept confidential and the subject's name will not be released at any time. Subject records will not be released to anyone other than NKTT or its designee(s), and responsible government agencies, when requested. In all cases, caution will be exercised to assure the subject's confidentiality. Datasets for each subject will be identified by a unique number.

15.5. Financial Disclosure

In compliance with 21 CFR Part 54, any listed or identified investigator or subinvestigator (including the spouse and any dependent children of said individuals) directly involved in the treatment or evaluation of research subjects will disclose the following information for the time period during which the investigator is participating in the study and for one year following completion of the study:

1. Any financial arrangement entered into between the Sponsors and the investigator, whereby the value of the compensation to the investigator for conducting the study could be influenced by the outcome of the study;

2. Any other significant payments totaling > $25,000, exclusive of the costs of conducting this or other clinical studies, by the Sponsors, such as a grant to fund ongoing research, compensation in the form of equipment, retainer for ongoing consultation, or honoraria;

3. Any proprietary interest in the product being evaluated;

4. Any significant equity interest, including ownership interest, stock options, or other financial interest whose value cannot be determined through reference to public prices or any equity interest in NKTT that exceeds $50,000.

16. DATA HANDLING AND RECORDKEEPING

16.1. Inspection of Records

NKTT or representative will be allowed to conduct site visits to the investigation facilities for the purpose of monitoring any aspect of the study. The Investigator agrees to allow the monitor to inspect the drug storage area, study drug stocks, drug accountability records, subject charts and study source documents, and other records relative to study conduct.

16.2. Retention of Records

The Principal Investigator must maintain all documentation relating to the study for a period of 2 years after the last marketing application approval, or if not approved 2 years following the discontinuance of the test article for investigation. If it becomes necessary for NKTT or the Regulatory Authority to review any documentation relating to the study, the Investigator must permit access to such records.

16.3. PUBLICATION POLICY

The information obtained during the conduct of this clinical study is confidential, and disclosure to third parties other than those noted below is prohibited. All information concerning the product as well as any matter concerning the operation of the Sponsor, such as clinical indications for the drug, its formula, methods of manufacture and other scientific data relating to it, that have been provided by the Sponsor and are unpublished, are confidential and must remain the sole property of the Sponsor. The investigator will agree to use the information only for the purposes of carrying out this study and for no other purpose unless prior written permission from the Sponsor is obtained.

Information obtained during the conduct of this study will be used by NKTT in connection with the development of the study drug. The study Investigator is obliged to provide NKTT with complete test results and all data developed in this study. The Sponsor has full ownership of the original case report forms completed as part of the study. This information may be disclosed to other physicians who are conducting similar studies and to the FDA as deemed necessary by the Sponsor. Subject specific information may be provided to other appropriate medical personnel related to the care of that subject only with subject's prior consent.

To ensure compliance with current Federal Regulations and the ICH guidelines, data generated by this study must be available for inspection upon request by representatives of the FDA and other regulatory agencies, national and local health authorities, NKTT and the IRB for each study site.

The Publication Committee (PC) will be responsible for preparing and submitting the primary clinical publication arising from this study. The membership for this committee consists of the Principal Investigator(s) and co-investigators, and NKTT designee. Any additional presentations and publications arising from the study must also be approved by the PC, which will undertake to review such publications within 30 days of receipt. Authorship for all presentations and publications will be decided by the PC, based on contributions to study concept, design and

execution. Subject names and other identifiers, such as photographs, audio or videotapes, may not be disclosed in any publication without prior written authorization from the subject.

17. LIST OF REFERENCES

Akbari, O., J. L. Faul, E. G. Hoyte, G. J. Berry, J. Wahlstrom, M. Kronenberg, R. H. DeKruyff, and D. T. Umetsu. 2006. CD4+ invariant T-cell-receptor+ natural killer T cells in bronchial asthma. N Engl J Med 354:1117-1129.

Albayrak, M., Z. Kaya, E. Yilmaz-Keskin, U. Z. Stadt, U. Kocak, and T. Gursel. 2009. Fatal Epstein-Barr virus infection in a case of familial hemophagocytic lymphohistiocytosis with syntaxin-11 mutation. The Turkish journal of pediatrics 51:371-374.

Arrunategui-Correa, V., and H. S. Kim. 2004. The role of CD1d in the immune response against Listeria infection. Cell Immunol 227:109-120.

Ashkar, A. A., and K. L. Rosenthal. 2003. Interleukin-15 and natural killer and NKT cells play a critical role in innate protection against genital herpes simplex virus type 2 infection. J Virol 77:10168-10171.

Ashley-Koch, A., Q. Yang, and R. S. Olney. 2000. Sickle hemoglobin (HbS) allele and sickle cell disease: a HuGE review. American journal of epidemiology 151:839-845.

Astrakhan, A., H. D. Ochs, and D. J. Rawlings. 2009. Wiskott-Aldrich syndrome protein is required for homeostasis and function of invariant NKT cells. J Immunol 182:7370-7380.

Balato, A., D. Unutmaz, and A. A. Gaspari. 2009. Natural killer T cells: an unconventional T-cell subset with diverse effector and regulatory functions. The Journal of investigative dermatology 129:1628-1642.

Barbaud, A., F. Granel, J. Waton, and C. Poreaux. 2011. How to manage hypersensitivity reactions to biological agents? European journal of dermatology : EJD 21:667-674.

Berzins, S. P., M. J. Smyth, and A. G. Baxter. 2011. Presumed guilty: natural killer T cell defects and human disease. Nat Rev Immunol 11:131-142.

Bedel, R., J. L. Matsuda, M. Brigl, J. White, J. Kappler, P. Marrack, and L. Gapin. 2012. Lower TCR repertoire diversity in Traj18-deficient mice. Nat Immunol 13:705-706.

Bendelac, A., M. N. Rivera, S. H. Park, and J. H. Roark. 1997. Mouse CD1-specific NK1 T cells: development, specificity, and function. Annual review of immunology 15:535-562.

Bendelac, A., P. B. Savage, and L. Teyton. 2007. The biology of NKT cells. Annual review of immunology 25:297-336.

Berzofsky, J. A., and M. Terabe. 2009. The contrasting roles of NKT cells in tumor immunity. Curr Mol Med 9:667-672.

Brandow, A. M., D. L. Jirovec, and J. A. Panepinto. 2010. Hydroxyurea in children with sickle cell disease: practice patterns and barriers to utilization. American journal of hematology 85:611-613.

Brawley, O. W., L. J. Cornelius, L. R. Edwards, V. N. Gamble, B. L. Green, C. Inturrisi, A. H. James, D. Laraque, M. Mendez, C. J. Montoya, B. H. Pollock, L. Robinson, A. P. Scholnik, and M. Schori. 2008. National Institutes of Health Consensus Development Conference statement: hydroxyurea treatment for sickle cell disease. Ann Intern Med 148:932-938.

Brousseau, D. C., P. L. Owens, A. L. Mosso, J. A. Panepinto, and C. A. Steiner. 2010. Acute care utilization and rehospitalizations for sickle cell disease. JAMA : the journal of the American Medical Association 303:1288-1294.

Brousseau, D. C., J. A. Panepinto, M. Nimmer, and R. G. Hoffmann. 2010. The number of people with sickle-cell disease in the United States: national and state estimates. American journal of hematology 85:77-78.

Broxmeyer, H. E., A. Dent, S. Cooper, G. Hangoc, Z. Y. Wang, W. Du, J. Gervay-Haque, V. Sriram, G. J. Renukaradhya, and R. R. Brutkiewicz. 2007. A role for natural killer T cells and CD1d molecules in counteracting suppression of hematopoiesis in mice induced by infection with murine cytomegalovirus. Exp Hematol 35:87-93.

Candrilli, S. D., S. H. O'Brien, R. E. Ware, M. C. Nahata, E. E. Seiber, and R. Balkrishnan. 2011. Hydroxyurea adherence and associated outcomes among Medicaid enrollees with sickle cell disease. American journal of hematology 86:273-277.

Chang, M. O., T. Suzuki, H. Suzuki, and H. Takaku. 2012. HIV-1 Gag-virus-like particles induce natural killer cell immune responses via activation and maturation of dendritic cells. Journal of innate immunity 4:187-200.

Chang, P. P., P. Barral, J. Fitch, A. Pratama, C. S. Ma, A. Kallies, J. J. Hogan, V. Cerundolo, S. G. Tangye, R. Bittman, S. L. Nutt, R. Brink, D. I. Godfrey, F. D. Batista, and C. G. Vinuesa. 2011. Identification of Bcl-6-dependent follicular helper NKT cells that provide cognate help for B cell responses. Nat Immunol 13:35-43.

Chang, Y. J., J. R. Huang, Y. C. Tsai, J. T. Hung, D. Wu, M. Fujio, C. H. Wong, and A. L. Yu. 2007. Potent immune-modulating and anticancer effects of NKT cell stimulatory glycolipids. Proc Natl Acad Sci U S A 104:10299-10304.

Charache, S., M. L. Terrin, R. D. Moore, G. J. Dover, F. B. Barton, S. V. Eckert, R. P. McMahon, and D. R. Bonds. 1995. Effect of hydroxyurea on the frequency of painful crises in sickle cell anemia. Investigators of the Multicenter Study of Hydroxyurea in Sickle Cell Anemia. N Engl J Med 332:1317-1322.

Field, J. J., D. G. Nathan, and J. Linden. 2011. Targeting iNKT cells for the treatment of sickle cell disease. Clin Immunol 140:177-183.

Frenette, P. S., and G. F. Atweh. 2007. Sickle cell disease: old discoveries, new concepts, and future promise. The Journal of clinical investigation 117:850-858.

Fujii, S., K. Shimizu, H. Hemmi, and R. M. Steinman. 2007. Innate Valpha14(+) natural killer T cells mature dendritic cells, leading to strong adaptive immunity. Immunol Rev 220:183-198.

Fujii, S., K. Shimizu, V. Klimek, M. D. Geller, S. D. Nimer, and M. V. Dhodapkar. 2003. Severe and selective deficiency of interferon-gamma-producing invariant natural killer T cells in patients with myelodysplastic syndromes. British journal of haematology 122:617-622.

Galli, G., S. Nuti, S. Tavarini, L. Galli-Stampino, C. De Lalla, G. Casorati, P. Dellabona, and S. Abrignani. 2003. CD1d-restricted help to B cells by human invariant natural killer T lymphocytes. The Journal of experimental medicine 197:1051-1057.

Giaccone, G., C. J. Punt, Y. Ando, R. Ruijter, N. Nishi, M. Peters, B. M. von Blomberg, R. J. Scheper, H. J. van der Vliet, A. J. van den Eertwegh, M. Roelvink, J. Beijnen, H. Zwierzina, and H. M. Pinedo. 2002. A phase I study of the natural killer T-cell ligand alpha-galactosylceramide (KRN7000) in patients with solid tumors. Clinical cancer research : an official journal of the American Association for Cancer Research 8:3702-3709.

Grant, A. M., C. S. Parker, L. B. Jordan, M. M. Hulihan, M. S. Creary, M. A. Lloyd-Puryear, J. C. Goldsmith, and H. K. Atrash. 2011. Public health implications of sickle cell trait: a report of the CDC meeting. American journal of preventive medicine 41:S435-439.

Grubor-Bauk, B., A. Simmons, G. Mayrhofer, and P. G. Speck. 2003. Impaired clearance of herpes simplex virus type 1 from mice lacking CD1d or NKT cells expressing the semivariant V alpha 14-J alpha 281 TCR. J Immunol 170:1430-1434.

Harding, M., and P. Kubes. 2012. Innate immunity in the vasculature: interactions with pathogenic bacteria. Current opinion in microbiology 15:85-91.

Hebbel, R. P., R. Osarogiagbon, and D. Kaul. 2004. The endothelial biology of sickle cell disease: inflammation and a chronic vasculopathy. Microcirculation 11:129-151.

Kaul, D. K., and R. P. Hebbel. 2000. Hypoxia/reoxygenation causes inflammatory response in transgenic sickle mice but not in normal mice. The Journal of clinical investigation 106:411-420.

Kim, E. Y., J. T. Battaile, A. C. Patel, Y. You, E. Agapov, M. H. Grayson, L. A. Benoit, D. E. Byers, Y. Alevy, J. Tucker, S. Swanson, R. Tidwell, J. W. Tyner, J. D. Morton, M. Castro, D. Polineni, G. A. Patterson, R. A. Schwendener, J. D. Allard, G. Peltz, and M. J. Holtzman. 2008. Persistent activation of an innate immune response translates respiratory viral infection into chronic lung disease. Nat Med 14:633-640.

King, I. L., A. Fortier, M. Tighe, J. Dibble, G. F. Watts, N. Veerapen, A. M. Haberman, G. S. Besra, M. Mohrs, M. B. Brenner, and E. A. Leadbetter. 2012. Invariant natural killer T cells direct B cell responses to cognate lipid antigen in an IL-21-dependent manner. Nat Immunol 13:44-50.

Kosits, C., and M. Callaghan. 2000. Rituximab: a new monoclonal antibody therapy for non-Hodgkin's lymphoma. Oncology nursing forum 27:51-59.

Lappas, C. M., Y. J. Day, M. A. Marshall, V. H. Engelhard, and J. Linden. 2006. Adenosine A2A receptor activation reduces hepatic ischemia reperfusion injury by inhibiting CD1d-dependent NKT cell activation. The Journal of experimental medicine 203:2639-2648.

Lee, W. Y., T. J. Moriarty, C. H. Wong, H. Zhou, R. M. Strieter, N. van Rooijen, G. Chaconas, and P. Kubes. 2010. An intravascular immune response to Borrelia burgdorferi involves Kupffer cells and iNKT cells. Nat Immunol 11:295-302.

Matangkasombut, P., G. Marigowda, A. Ervine, L. Idris, M. Pichavant, H. Y. Kim, T. Yasumi, S. B. Wilson, R. H. DeKruyff, J. L. Faul, E. Israel, O. Akbari, and D. T. Umetsu. 2009. Natural killer T cells in the lungs of patients with asthma. J Allergy Clin Immunol 123:1181-1185.

Matsuda, J. L., L. Gapin, J. L. Baron, S. Sidobre, D. B. Stetson, M. Mohrs, R. M. Locksley, and M. Kronenberg. 2003. Mouse V alpha 14i natural killer T cells are resistant to cytokine polarization in vivo. Proc Natl Acad Sci U S A 100:8395-8400.

Montoya, C. J., J. C. Catano, Z. Ramirez, M. T. Rugeles, S. B. Wilson, and A. L. Landay. 2008. Invariant NKT cells from HIV-1 or Mycobacterium tuberculosis-infected patients express an activated phenotype. Clin Immunol 127:1-6.

Motohashi, S., A. Ishikawa, E. Ishikawa, M. Otsuji, T. Iizasa, H. Hanaoka, N. Shimizu, S. Horiguchi, Y. Okamoto, S. Fujii, M. Taniguchi, T. Fujisawa, and T. Nakayama. 2006. A phase I study of in vitro expanded natural killer T cells in patients with advanced and recurrent non-small cell lung cancer. Clinical cancer research : an official journal of the American Association for Cancer Research 12:6079-6086.

Motohashi, S., K. Nagato, N. Kunii, H. Yamamoto, K. Yamasaki, K. Okita, H. Hanaoka, N. Shimizu, M. Suzuki, I. Yoshino, M. Taniguchi, T. Fujisawa, and T. Nakayama. 2009. A phase I-II study of alpha-galactosylceramide-pulsed IL-2/GM-CSF-cultured peripheral blood mononuclear cells in patients with advanced and recurrent non-small cell lung cancer. J Immunol 182:2492-2501.

Nakai, Y., K. Iwabuchi, S. Fujii, N. Ishimori, N. Dashtsoodol, K. Watano, T. Mishima, C. Iwabuchi, S. Tanaka, J. S. Bezbradica, T. Nakayama, M. Taniguchi, S. Miyake, T. Yamamura, A. Kitabatake, S. Joyce, L. Van Kaer, and K. Onoe. 2004. Natural killer T cells accelerate atherogenesis in mice. Blood 104:2051-2059.

Nieuwenhuis, E. E., T. Matsumoto, M. Exley, R. A. Schleipman, J. Glickman, D. T. Bailey, N. Corazza, S. P. Colgan, A. B. Onderdonk, and R. S. Blumberg. 2002. CD1d-dependent macrophage-mediated clearance of Pseudomonas aeruginosa from lung. Nat Med 8:588-593.

Novakova, L., A. Lehuen, and J. Novak. 2011. Low numbers and altered phenotype of invariant natural killer T cells in recurrent varicella zoster virus infection. Cell Immunol 269:78-81.

Orange, J. S. 2002. Human natural killer cell deficiencies and susceptibility to infection. Microbes Infect 4:1545-1558.

Platt, O. S. 2000. Sickle cell anemia as an inflammatory disease. The Journal of clinical investigation 106:337-338.

Platt, O. S., D. J. Brambilla, W. F. Rosse, P. F. Milner, O. Castro, M. H. Steinberg, and P. P. Klug. 1994. Mortality in sickle cell disease. Life expectancy and risk factors for early death. N Engl J Med 330:1639-1644.

Platt, O. S., B. D. Thorington, D. J. Brambilla, P. F. Milner, W. F. Rosse, E. Vichinsky, and T. R. Kinney. 1991. Pain in sickle cell disease. Rates and risk factors. N Engl J Med 325:11-16.

Ranson, T., S. Bregenholt, A. Lehuen, O. Gaillot, M. C. Leite-de-Moraes, A. Herbelin, P. Berche, and J. P. Di Santo. 2005. Invariant V alpha 14+ NKT cells participate in the early response to enteric Listeria monocytogenes infection. J Immunol 175:1137-1144.

Renukaradhya, G. J., M. A. Khan, M. Vieira, W. Du, J. Gervay-Hague, and R. R. Brutkiewicz. 2008. Type I NKT cells protect (and type II NKT cells suppress) the host's innate antitumor immune response to a B-cell lymphoma. Blood 111:5637-5645.

Reynolds, C., J. Barkans, P. Clark, H. Kariyawasam, D. Altmann, B. Kay, and R. Boyton. 2009. Natural killer T cells in bronchial biopsies from human allergen challenge model of allergic asthma. J Allergy Clin Immunol 124:860-862; author reply 862.

Sada-Ovalle, I., M. Skold, T. Tian, G. S. Besra, and S. M. Behar. 2010. Alpha-galactosylceramide as a therapeutic agent for pulmonary Mycobacterium tuberculosis infection. Am J Respir Crit Care Med 182:841-847.

Schneiders, F. L., R. J. Scheper, B. M. von Blomberg, A. M. Woltman, H. L. Janssen, A. J. van den Eertwegh, H. M. Verheul, T. D. de Gruijl, and H. J. van der Vliet. 2011. Clinical experience with alpha-galactosylceramide (KRN7000) in patients with advanced cancer and chronic hepatitis B/C infection. Clin Immunol 140:130-141.

Sireci, G., D. Russo, F. Dieli, S. A. Porcelli, M. Taniguchi, M. P. La Manna, D. Di Liberto, F. Scarpa, and A. Salerno. 2007. Immunoregulatory role of Jalpha281 T cells in aged mice developing lupus-like nephritis. Eur J Immunol 37:425-433.

Skold, M., and S. M. Behar. 2003. Role of CD1d-restricted NKT cells in microbial immunity. Infect Immun 71:5447-5455.

Smith, W. R., L. T. Penberthy, V. E. Bovbjerg, D. K. McClish, J. D. Roberts, B. Dahman, I. P. Aisiku, J. L. Levenson, and S. D. Roseff. 2008. Daily assessment of pain in adults with sickle cell disease. Ann Intern Med 148:94-101.

Sutherland, J. S., D. J. Jeffries, S. Donkor, B. Walther, P. C. Hill, I. M. Adetifa, R. A. Adegbola, and M. O. Ota. 2009. High granulocyte/lymphocyte ratio and paucity of NKT cells defines TB disease in a TB-endemic setting. Tuberculosis (Edinb) 89:398-404.

Swann, J. B., A. P. Uldrich, S. van Dommelen, J. Sharkey, W. K. Murray, D. I. Godfrey, and M. J. Smyth. 2009. Type I natural killer T cells suppress tumors caused by p53 loss in mice. Blood 113:6382-6385.

Tahir, S. M., O. Cheng, A. Shaulov, Y. Koezuka, G. J. Bubley, S. B. Wilson, S. P. Balk, and M. A. Exley. 2001. Loss of IFN-gamma production by invariant NK T cells in advanced cancer. J Immunol 167:4046-4050.

Tupin, E., Y. Kinjo, and M. Kronenberg. 2007. The unique role of natural killer T cells in the response to microorganisms. Nat Rev Microbiol 5:405-417.

Uchida, T., S. Horiguchi, Y. Tanaka, H. Yamamoto, N. Kunii, S. Motohashi, M. Taniguchi, T. Nakayama, and Y. Okamoto. 2008. Phase I study of alpha-galactosylceramide-pulsed antigen presenting cells administration to the nasal submucosa in unresectable or recurrent head and neck cancer. Cancer immunology, immunotherapy : CII 57:337-345.

van der Vliet, H. J., B. M. von Blomberg, N. Nishi, M. Reijm, A. E. Voskuyl, A. A. van Bodegraven, C. H. Polman, T. Rustemeyer, P. Lips, A. J. van den Eertwegh, G. Giaccone, R. J. Scheper, and H. M. Pinedo. 2001. Circulating V(alpha24+) Vbeta11+ NKT cell numbers are decreased in a wide variety of diseases that are characterized by autoreactive tissue damage. Clin Immunol 100:144-148.

van der Vliet, H. J., R. Wang, S. C. Yue, H. B. Koon, S. P. Balk, and M. A. Exley. 2008. Circulating myeloid dendritic cells of advanced cancer patients result in reduced activation and a biased cytokine profile in invariant NKT cells. J Immunol 180:7287-7293.

Veldt, B. J., H. J. van der Vliet, B. M. von Blomberg, H. van Vlierberghe, G. Gerken, N. Nishi, K. Hayashi, R. J. Scheper, R. J. de Knegt, A. J. van den Eertwegh, H. L. Janssen, and C. M. van

Nieuwkerk. 2007. Randomized placebo controlled phase I/II trial of alpha-galactosylceramide for the treatment of chronic hepatitis C. Journal of hepatology 47:356-365.

Vincent, M. S., J. E. Gumperz, and M. B. Brenner. 2003. Understanding the function of CD1-restricted T cells. Nat Immunol 4:517-523.

Wallace, K. L., and J. Linden. 2010. Adenosine A2A receptors induced on iNKT and NK cells reduce pulmonary inflammation and injury in mice with sickle cell disease. Blood 116:5010-5020.

Wallace, K. L., M. A. Marshall, S. I. Ramos, J. A. Lannigan, J. J. Field, R. M. Strieter, and J. Linden. 2009. NKT cells mediate pulmonary inflammation and dysfunction in murine sickle cell disease through production of IFN-gamma and CXCR3 chemokines. Blood 114:667-676.

Weber, R. W. 2004. Adverse reactions to biological modifiers. Current opinion in allergy and clinical immunology 4:277-283.

Wu, L., and L. Van Kaer. 2009. Natural killer T cells and autoimmune disease. Curr Mol Med 9:4-14.

Wu, L., and L. Van Kaer. 2011. Natural killer T cells in health and disease. Front Biosci (Schol Ed) 3:236-251.

Yoshimoto, T., B. Min, T. Sugimoto, N. Hayashi, Y. Ishikawa, Y. Sasaki, H. Hata, K. Takeda, K. Okumura, L. Van Kaer, W. E. Paul, and K. Nakanishi. 2003. Nonredundant roles for CD1d-restricted natural killer T cells and conventional CD4+ T cells in the induction of immunoglobulin E antibodies in response to interleukin 18 treatment of mice. The Journal of experimental medicine 197:997-1005.

Yusuf, H. R., M. A. Lloyd-Puryear, A. M. Grant, C. S. Parker, M. S. Creary, and H. K. Atrash. 2011. Sickle cell disease: the need for a public health agenda. American journal of preventive medicine 41:S376-383.

Zeissig, S., and R. S. Blumberg. 2012. Primary immunodeficiency associated with defects in CD1 and CD1-restricted T cells. Ann N Y Acad Sci 1250:14-24.

18. APPENDICES

18.1. CDC-recommended immunizations for adults

Recommended Immunizations for Adults

If you are this age,

Then you should get these vaccines:	19 - 21 years	22 - 26 years	27 - 49 years	50 - 59 years	60 - 64 years	65+ years
Influenza (Flu)	Get a flu vaccine every year					
Tetanus, diphtheria, pertussis (Td/Tdap)	Get a Tdap vaccine once, then a Td booster vaccine every 10 years					
Varicella (Chickenpox)	2 doses					
HPV Vaccine for Women	3 doses					
HPV Vaccine for Men	3 doses	3 doses				
Zoster (Shingles)					1 dose	
Measles, mumps, rubella (MMR)	1 or 2 doses			1 or 2 doses		
Pneumococcal (pneumonia)	1 or 2 doses					1 dose
Meningococcal	1 or more doses					
Hepatitis A	2 doses					
Hepatitis B	3 doses					

- (Light blue) Boxes this color show that the vaccine is recommended for all adults unless your doctor or nurse tells you that you cannot safely receive the vaccine.
- (Bright blue) Boxes this color show when the vaccine is recommended for adults with certain risks related to their health, job or lifestyle that put them at higher risk for serious diseases. Talk to your doctor or nurse to see if you are at higher risk.
- (White) No recommendation

FOOTNOTES:

(Influenza vaccine) [1]There are four different flu vaccines available—talk to your doctor or nurse about which flu vaccine is right for you.

(HPV vaccine for men) [2]There are two different kinds of HPV vaccine but only one HPV vaccine (Gardasil®) can be given to men. Gay men or men who have sex with men who are 22 through 26 years old should get HPV vaccine if they haven't already started or completed the series.

(MMR vaccine) [3]If you were born in 1957 or after, you should have already gotten MMR vaccine. Talk to your doctor or nurse about how many doses you may need.

If you are traveling outside of the United States, you may need additional vaccines. Ask your doctor or nurse which vaccines you may need.

For more information, call toll free 1-800-CDC-INFO (1-800-232-4636) or visit http://www.cdc.gov/vaccines

U.S. Department of Health and Human Services
Centers for Disease Control and Prevention

18.2. Management of infusion reactions

Should CRS of any type be observed in subjects in any cohort, premedication with acetaminophen (1000 mg), histamine 2 receptor antagonists (diphenhydramine, 50mg) and corticosteroids may be initiated prior to dosing of subsequent subjects to lessen severity of CRS.

Should the subject experience any local discomfort or adverse event during the infusion, the infusion should be stopped until the discomfort has resolved or the subject stabilized. Stopping the infusion and administration a histamine blocker is usually sufficient to manage reactions for most subjects who experience mild or moderate infusion reactions.

Once symptoms subside, usually within 30 minutes, the infusion can often be restarted cautiously at the investigator's discretion, at half the rate and titrated as tolerated without further symptoms

If restarted, the infusion should be slowed to deliver 2.5 mL/min.

If there is no further reaction, continue at 50% rate of infusion **or** resume original rate of infusion.

The infusion should not be restarted if the subject's symptoms have not resolved within one hour.

The infusion should not be restarted if the subject experienced a severe reaction.

18.3. World Medical Association Declaration of Helsinki

Ethical Principles for Medical Research Involving Human Subjects

Adopted by the 18th WMA General Assembly, Helsinki, Finland, June 1964, and amended by the:

29th WMA General Assembly, Tokyo, Japan, October 1975
35th WMA General Assembly, Venice, Italy, October 1983
41st WMA General Assembly, Hong Kong, September 1989
48th WMA General Assembly, Somerset West, Republic of South Africa, October 1996
52nd WMA General Assembly, Edinburgh, Scotland, October 2000
53rd WMA General Assembly, Washington 2002 (Note of Clarification on paragraph 29 added)
55th WMA General Assembly, Tokyo 2004 (Note of Clarification on Paragraph 30 added)
59th WMA General Assembly, Seoul, October 2008

A. INTRODUCTION

1. The World Medical Association (WMA) has developed the Declaration of Helsinki as a statement of ethical principles for medical research involving human subjects, including research on identifiable human material and data. The Declaration is intended to be read as a whole and each of its constituent paragraphs should not be applied without consideration of all other relevant paragraphs.
2. Although the Declaration is addressed primarily to physicians, the WMA encourages other participants in medical research involving human subjects to adopt these principles.
3. It is the duty of the physician to promote and safeguard the health of patients, including those who are involved in medical research. The physician's knowledge and conscience are dedicated to the fulfilment of this duty.
4. The Declaration of Geneva of the WMA binds the physician with the words, “The health of my patient will be my first consideration,” and the International Code of Medical Ethics declares that, “A physician shall act in the patient's best interest when providing medical care.”
5. Medical progress is based on research that ultimately must include studies involving human subjects. Populations that are underrepresented in medical research should be provided appropriate access to participation in research.
6. In medical research involving human subjects, the well-being of the individual research subject must take precedence over all other interests.
7. The primary purpose of medical research involving human subjects is to understand the causes, development and effects of diseases and improve preventive, diagnostic and therapeutic interventions (methods, procedures and treatments). Even the best current interventions must be evaluated continually through research for their safety, effectiveness, efficiency, accessibility and quality.
8. In medical practice and in medical research, most interventions involve risks and burdens.

9. Medical research is subject to ethical standards that promote respect for all human subjects and protect their health and rights. Some research populations are particularly vulnerable and need special protection. These include those who cannot give or refuse consent for themselves and those who may be vulnerable to coercion or undue influence.
10. Physicians should consider the ethical, legal and regulatory norms and standards for research involving human subjects in their own countries as well as applicable international norms and standards. No national or international ethical, legal or regulatory requirement should reduce or eliminate any of the protections for research subjects set forth in this Declaration.

B. PRINCIPLES FOR ALL MEDICAL RESEARCH

11. It is the duty of physicians who participate in medical research to protect the life, health, dignity, integrity, right to self-determination, privacy, and confidentiality of personal information of research subjects.
12. Medical research involving human subjects must conform to generally accepted scientific principles, be based on a thorough knowledge of the scientific literature, other relevant sources of information, and adequate laboratory and, as appropriate, animal experimentation. The welfare of animals used for research must be respected.
13. Appropriate caution must be exercised in the conduct of medical research that may harm the environment.
14. The design and performance of each research study involving human subjects must be clearly described in a research protocol. The protocol should contain a statement of the ethical considerations involved and should indicate how the principles in this Declaration have been addressed. The protocol should include information regarding funding, sponsors, institutional affiliations, other potential conflicts of interest, incentives for subjects and provisions for treating and/or compensating subjects who are harmed as a consequence of participation in the research study. The protocol should describe arrangements for post-study access by study subjects to interventions identified as beneficial in the study or access to other appropriate care or benefits.
15. The research protocol must be submitted for consideration, comment, guidance and approval to a research ethics committee before the study begins. This committee must be independent of the researcher, the sponsor and any other undue influence. It must take into consideration the laws and regulations of the country or countries in which the research is to be performed as well as applicable international norms and standards but these must not be allowed to reduce or eliminate any of the protections for research subjects set forth in this Declaration. The committee must have the right to monitor ongoing studies. The researcher must provide monitoring information to the committee, especially information about any serious adverse events. No change to the protocol may be made without consideration and approval by the committee.
16. Medical research involving human subjects must be conducted only by individuals with the appropriate scientific training and qualifications. Research on patients or healthy volunteers requires the supervision of a competent and appropriately qualified physician or other health care professional. The responsibility for the protection of research subjects

must always rest with the physician or other health care professional and never the research subjects, even though they have given consent.

17. Medical research involving a disadvantaged or vulnerable population or community isonly justified if the research is responsive to the health needs and priorities of this population or community and if there is a reasonable likelihood that this population or community stands to benefit from the results of the research.
18. Every medical research study involving human subjects must be preceded by careful assessment of predictable risks and burdens to the individuals and communities involved in the research in comparison with foreseeable benefits to them and to other individuals or communities affected by the condition under investigation.
19. Every clinical trial must be registered in a publicly accessible database before recruitment of the first subject.
20. Physicians may not participate in a research study involving human subjects unless they are confident that the risks involved have been adequately assessed and can be satisfactorily managed. Physicians must immediately stop a study when the risks are found to outweigh the potential benefits or when there is conclusive proof of positive and beneficial results.
21. Medical research involving human subjects may only be conducted if the importance of the objective outweighs the inherent risks and burdens to the research subjects.
22. Participation by competent individuals as subjects in medical research must be voluntary. Although it may be appropriate to consult family members or community leaders, no competent individual may be enrolled in a research study unless he or she freely agrees.
23. Every precaution must be taken to protect the privacy of research subjects and the confidentiality of their personal information and to minimize the impact of the study on their physical, mental and social integrity.
24. In medical research involving competent human subjects, each potential subject must be adequately informed of the aims, methods, sources of funding, any possible conflicts of interest, institutional affiliations of the researcher, the anticipated benefits and potential risks of the study and the discomfort it may entail, and any other relevant aspects of the study. The potential subject must be informed of the right to refuse to participate in the study or to withdraw consent to participate at any time without reprisal. Special attention should be given to the specific information needs of individual potential subjects as well as to the methods used to deliver the information. After ensuring that the potential subject has understood the information, the physician or another appropriately qualified individual must then seek the potential subject's freely-given informed consent, preferably in writing. If the consent cannot be expressed in writing, the non-written consent must be formally documented and witnessed.
25. For medical research using identifiable human material or data, physicians must normally seek consent for the collection, analysis, storage and/or reuse. There may be situations where consent would be impossible or impractical to obtain for such research or would pose a threat to the validity of the research. In such situations the research may be done only after consideration and approval of a research ethics committee.

26. When seeking informed consent for participation in a research study the physician should be particularly cautious if the potential subject is in a dependent relationship with the physician or may consent under duress. In such situations the informed consent should be sought by an appropriately qualified individual who is completely independent of this relationship.
27. For a potential research subject who is incompetent, the physician must seek informed consent from the legally authorized representative. These individuals must not be included in a research study that has no likelihood of benefit for them unless it is intended to promote the health of the population represented by the potential subject, the research cannot instead be performed with competent persons, and the research entails only minimal risk and minimal burden.
28. When a potential research subject who is deemed incompetent is able to give assent to decisions about participation in research, the physician must seek that assent in addition to the consent of the legally authorized representative. The potential subject's dissent should be respected.
29. Research involving subjects who are physically or mentally incapable of giving consent, for example, unconscious patients, may be done only if the physical or mental condition that prevents giving informed consent is a necessary characteristic of the research population. In such circumstances the physician should seek informed consent from the legally authorized representative. If no such representative is available and if the research cannot be delayed, the study may proceed without informed consent provided that the specific reasons for involving subjects with a condition that renders them unable to give informed consent have been stated in the research protocol and the study has been approved by a research ethics committee. Consent to remain in the research should be obtained as soon as possible from the subject or a legally authorized representative.
30. Authors, editors and publishers all have ethical obligations with regard to the publication of the results of research. Authors have a duty to make publicly available the results of their research on human subjects and are accountable for the completeness and accuracy of their reports. They should adhere to accepted guidelines for ethical reporting. Negative and inconclusive as well as positive results should be published or otherwise made publicly available. Sources of funding, institutional affiliations and conflicts of interest should be declared in the publication. Reports of research not in accordance with the principles of this Declaration should not be accepted for publication.

C. ADDITIONAL PRINCIPLES FOR MEDICAL RESEARCH COMBINED WITH MEDICAL CARE

31. The physician may combine medical research with medical care only to the extent that the research is justified by its potential preventive, diagnostic or therapeutic value and if the physician has good reason to believe that participation in the research study will not adversely affect the health of the patients who serve as research subjects.
32. The benefits, risks, burdens and effectiveness of a new intervention must be tested against those of the best current proven intervention, except in the following circumstances:

- The use of placebo, or no treatment, is acceptable in studies where no current proven intervention exists; or
- Where for compelling and scientifically sound methodological reasons the use of placebo is necessary to determine the efficacy or safety of an intervention and the patients who receive placebo or no treatment will not be subject to any risk of serious or irreversible harm. Extreme care must be taken to avoid abuse of this option.

33. At the conclusion of the study, patients entered into the study are entitled to be informed about the outcome of the study and to share any benefits that result from it, for example, access to interventions identified as beneficial in the study or to other appropriate care or benefits.
34. The physician must fully inform the patient which aspects of the care are related to the research. The refusal of a patient to participate in a study or the patient's decision to withdraw from the study must never interfere with the patient-physician relationship.
35. In the treatment of a patient, where proven interventions do not exist or have been ineffective, the physician, after seeking expert advice, with informed consent from the patient or a legally authorized representative, may use an unproven intervention if in the physician's judgment it offers hope of saving life, re-establishing health or alleviating suffering. Where possible, this intervention should be made the object of research, designed to evaluate its safety and efficacy. In all cases, new information should be recorded and, where appropriate, made publicly available.
